# Montagnulans A–D with Anti-Osteoclastogenic Activity from the Marine Fungus *Montagnula* sp. GXIMD 02514

**DOI:** 10.3390/md23110416

**Published:** 2025-10-25

**Authors:** Miaoping Lin, Humu Lu, Jiaxi Wang, Huangxue Qin, Xinya Xu, Chenghai Gao, Yonghong Liu, Yanhui Tan, Xiaowei Luo

**Affiliations:** 1 Guangxi Key Laboratory of Marine Drugs, University Engineering Research Center of High-Efficient Utilization of Marine Traditional Chinese Medicine Resources, Guangxi, Institute of Marine Drugs, Guangxi University of Chinese Medicine, Nanning 530200, China; 2 State Key Laboratory for Chemistry and Molecular Engineering of Medicinal Resources, School of Chemistry and Pharmaceutical Sciences, Guangxi Normal University, Guilin 541004, China

**Keywords:** marine fungi, *Montagnula* sp., tetramic acids, osteoclast differentiation

## Abstract

Four novel tetramic acid compounds, montagnulans A–D (**1**–**4**), were obtained from the Beibu Gulf coral-associated fungus *Montagnula* sp. GXIMD 02514. Their structures were determined by comprehensive physicochemical and spectroscopic data interpretation. The absolute configurations were accomplished by ECD calculations. Structurally, compounds **1**–**4** were rare leucine-derived tetramic acids bearing an ethyl (**1**–**3**) or hexylenic alcohol (**4**) side chain and a pyranone ring at C-3 of the 2,4-pyrrolidinedione core. Compound **1** exhibited inhibition of lipopolysaccharide (LPS)-induced NF-*κ*B in RAW 264.7 macrophages at 20 μM, which further inhibited RANKL-induced osteoclast differentiation without cytotoxicity in bone marrow macrophages cells (BMMs). This is the first report of osteoclastogenesis inhibitions for tetramic acids, which sheds light on their development as potential osteoclast differentiation inhibitors.

## 1. Introduction

Osteoporosis is known as a common bone disease characterized by the systemic impairment of bone mass and microstructural degradation of bone tissue that leads to bone fragility and susceptibility to fractures [1], which poses a severe public health threat owing to the increased ageing population, particularly for postmenopausal women and the elderly [2]. Bone metabolic homeostasis is generally regulated by the bone remodeling balance between bone-resorbing osteoclasts and bone-forming osteoblasts [3]. Excessive activation of osteoclasts dysregulates osteoclast formation and function and results in bone loss in osteolytic diseases, including osteoporosis, Paget’s disease, rheumatoid arthritis, and metastatic cancers [4]. Targeting osteoclast differentiation has been an effective therapeutic strategy for osteolytic diseases. However, the two clinically representative therapeutic agents for targeting osteoclasts, bisphosphonate zoledronic acid and denosumab, were found to have serious complications and intolerable side effects such as osteonecrosis of the jaw that could cause disability [5]. Therefore, novel osteoclast differentiation inhibitors with distinctive modes of action are urgently needed.

Tetramic acid (TA, pyrrolidine-2,4-dione) compounds with mixed biosynthetic origin are obtained from a variety of terrestrial and marine organisms, especially marine fungi [6,7]. They display a wide variety of biological activities, including anti-bacterial, anti-fungal, anti-viral, and anti-cancer activities, which have continuously attracted considerable attention from biologists and chemists [8,9]. Notably, tenuazonic acid was very recently characterized as a novel simple TA synthesized from the threonine precursor by *Alternaria alternata* and three other filamentous fungi, which showed significant herbicidal activity [10]. Lecanicilliumins A–G, 3-acyl TA derivatives, were obtained with nuclear factor-*κ*B (NF-*κ*B) inhibition from the deep-sea-derived fungus *Lecanicillium fusisporum* GXIMD00542 in our recent study [11].

**Figure 1:**
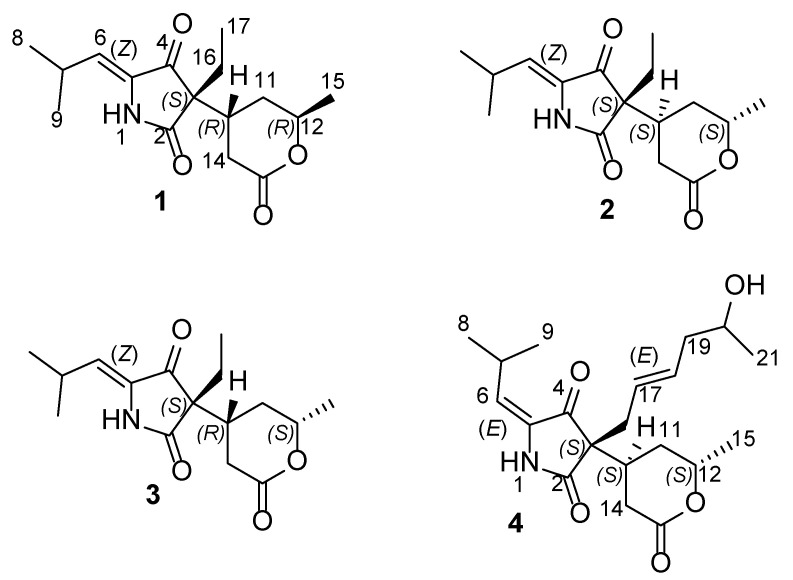
Chemical structures of compounds **1**–**4**.

During the course of our continuing endeavors searching for naturally occurring potent osteoclast differentiation inhibitors from marine fungi, a network of structurally diversified compounds was recently characterized, including indole diterpenoids [12,13], azaphilones [14], tanzawaic acids [15], depsidones [16], and nitrobenzoyl sesquiterpenoids [4]. The Beibu Gulf coral-associated fungus *Montagnula* sp. GXIMD 02514 caught our attention owing to rare studies on its secondary metabolites together with its interesting HPLC-UV profiles of the extract. Further chemical investigation enabled us to discover four rare leucine-derived TA compounds (Figure 1). Details of the isolation, structural elucidation, and anti-osteoclastogenic properties of these TAs are reported herein.

## 2. Results and Discussion

The fermented products of *Montagnula* sp. GXIMD 02514 on solid rice medium were extracted with EtOAc repeatedly. The whole extract was then subjected to various column chromatography, involving silica gel, reversed-phase silica gel C18, and semipreparative HPLC. The HPLC-DAD-guided isolation led to the discovery of four novel leucine-derived TA compounds.

Compound **1** was isolated as a white powder with the molecular formula C_16_H_23_NO_4_ (6 degrees of unsaturation, DOU) based on the HR-ESIMS data at *m*/*z* 316.1526 [M + Na]^+^ (calculated for C_16_H_23_NO_4_Na, 316.1525). An analysis of the ^1^H and ^13^C NMR data (Table 1 and Table 2) in conjunction with HSQC data of **1** displayed resonances corresponding to four methyls [*δ*_H/C_ 1.12 (3H, d, *J* = 7.5 Hz, H_3_-8)/22.2 (CH_3_, C-8), 1.10 (3H, d, *J* = 7.5 Hz, H_3_-9)/22.2 (CH_3_, C-9), 1.34 (3H, d, *J* = 6.0 Hz, H_3_-15)/20.8 (CH_3_, C-15), 0.84 (3H, t, *J* = 7.5 Hz, H_3_-17)/8.7 (CH_3_, C-17)], three methylenes [*δ*_H/C_ 1.84 (2H, q, *J* = 7.5 Hz, H_2_-16)/26.0 (CH_2_, C-16); 2.52, 2.62 (2H, m, H_2_-14)/30.5 (CH_2_, C-14); *δ*_H_ 1.98 (1H, m, H-11a), 1.75 (1H, m, H-11b)/30.3 (CH_2_, C-11)], and four methines [*δ*_H/C_ 2.45 (1H, m, H-7)/27.1 (CH, C-7), 5.61 (1H, d, *J* = 10.0 Hz, H-6)/119.1 (CH, C-6), 2.55 (1H, m, H-10)/33.2 (CH, C-10), 4.46 (1H, m, H-12)/73.7 (CH, C-12)]. Aside from the above 11 corresponding proton-bearing carbons, five quaternary carbon resonances remained in the ^13^C NMR data (Table 2), attributed to three carbonyls (*δ*_C_ 174.7, 200.0, and 171.7), one olefinic (*δ*_C_ 132.0), and one sp^3^ carbon (*δ*_C_ 57.1). The aforementioned spectral characteristics revealed a TA derivative, which were similar to those of lecanicilliumin A except for the substituent groups at C-3 and C-5 [11].

**Figure 2:**
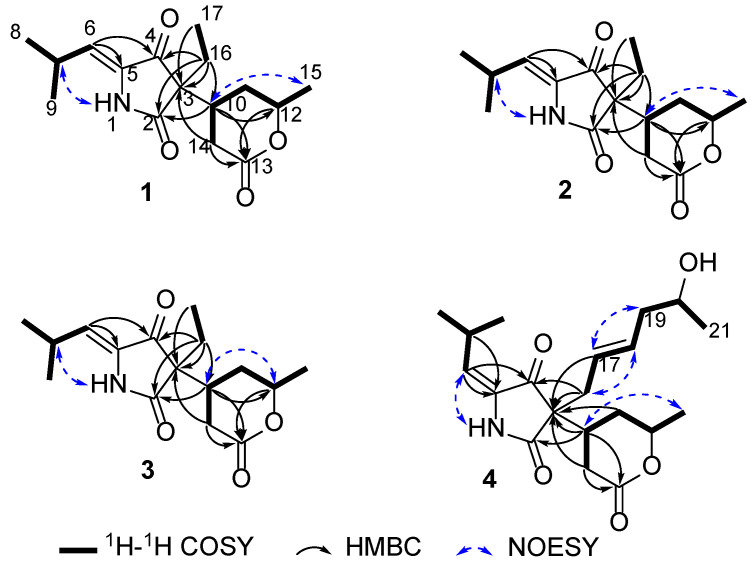
Key ^1^H−^1^H COSY, HMBC, and NOESY correlations of **1**–**4**.

The sequential ^1^H-^1^H COSY correlations (Figure 2) of H_3_-15/H-12/H_2_-11/H-10/H_2_-14 and H_2_-16/H_3_-17, along with HMBC correlations of H-10, H-12, H_2_-14/C-13, H-10/C-2, C-4, and H_3_-17/C-3, permitted the presence of a 6-methyltetrahydro-2*H*-pyran-2-one unit and an ethyl group attached at C-3 in **1**. Additionally, the ^1^H-^1^H COSY correlations of H_3_-8/H-7/H_3_-9 and HMBC correlations of H-7, H-6/C-5 allowed the existence of an isopropyl group at C-6 linked by the Δ^5^ double bond. Further interpretations of 2D NMR correlations confirmed the planar structure of **1**, which gave the trivial name montagnulan A.

**Figure 3:**
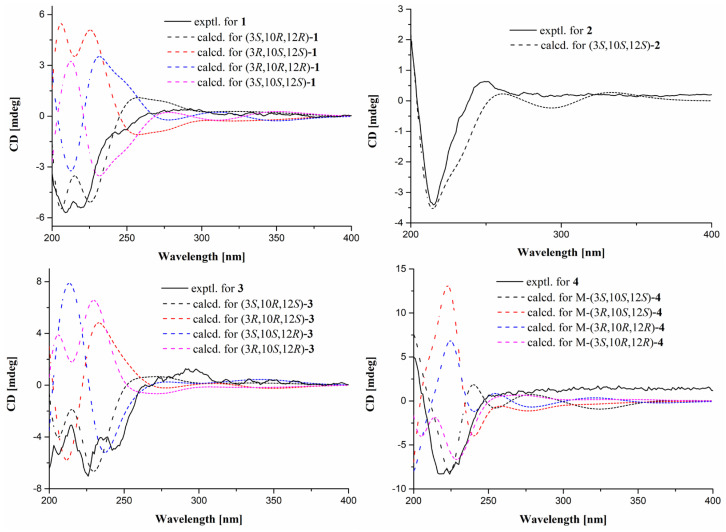
The experimental and calculated ECD spectra of **1**–**4**.

The relative configurations of **1** were assigned by NOESY correlations (Figure 2). The NOESY correlation of H-10/H_3_-15 suggested that both were located on the same side of the pyranone ring. The Δ^5^ double bond was determined as a *Z* configuration referring to the NOESY correlation of H-7/1-NH. Despite many attempts at developing a single crystal ending in failure, its absolute configurations were alternatively determined by ECD calculations. Based on the above discussion, there were four remaining isomers (3*S*, 10*R*, 12*R*; 3*R*, 10*S*, 12*S*; 3*R*, 10*R*, 12*R*; 3*S*, 10*S*, 12*S*) (Supplementary Figure S37) of **1**, which were further subjected to ECD calculations according to our previously reported approaches [17,18]. The experimental ECD curve of **1** matched well with the calculated curve of (3*S*, 10*R*, 12*R*)-**1** (Figure 3). Thus, the absolute configurations of **1** were assigned as 3*S*, 10*R*, 12*R*.

Montagnulan B (**2**) was found with the same molecular formula as was used for **1**, which relied on the HR-ESIMS data at *m*/*z* 316.1522 [M + Na] ^+^ (calculated for C_16_H_23_NO_4_Na 316.1525). Moreover, compound **2** shared nearly identical spectroscopic characteristics with those of **1**, except for slight differences in the partial chemical shifts of the pyranone ring, suggesting a pair of stereoisomers. A further analysis of NOESY correlations (Figure 2) revealed that compounds **2** and **1** also shared the same relative configurations. The ECD spectra of the 3-acyl TAs bearing an alcohol side chain and a six-membered lactone ring at C-3 are governed mainly by the C-3 chiral center according to these reported analogs, cladosporiumins A, B [7], I, and J [19]. Compounds **2** and **1** shared the prominent negative cotton effects at around 225 nm, which suggested it also shared the same 3*S* configuration but opposite configurations in the pyranone ring with that of **1** based on the above discussions. Further ECD calculations showed that the calculated ECD curve of (3*S*, 10*S*, 12*S*)-**2** was consistent with the experimental one (Figure 3), suggesting the absolute configurations of 3*S*, 10*S*, 12*S* in **2**.

The spectroscopic characteristics (Figure 2 and Table 1 and Table 2) of montagnulan C (**3**) indicated it also shared the same planar structure as **1** and **2**. The Δ^5^ double bond was determined as a *Z* configuration supported by the NOESY correlation of H-7/1-NH in **3**. However, the NOESY correlation of H-10/H-12 demonstrated that both were located on the same face of the pyranone ring. As in the case of **1**, four possible isomers of **3** were further subjected to ECD calculations. The experimental ECD curve of **3** showed good agreement with the calculated one of (3*S*, 10*R*, 12*S*)-**3** (Figure 3). Thus, compound **3** with (3*S*, 10*R*, 12*S*) configurations was a stereoisomer of **1** and **2**.

Montagnulan D (**4**) was isolated as a white powder with the molecular formula C_20_H_29_NO_5_ (7 DOU), as determined from the HR-ESIMS data at *m*/*z* 386.1944 [M + Na] ^+^ (calculated for C_20_H_29_NO_5_Na, 386.1943). The 1D NMR (Table 1 and Table 2) and HSQC data of **4** underscored typical TA signals indicative of four methyls, four methylenes, and seven methines, along with five hydrogen-lacking carbons ascribe to three carbonyls, one olefinic, and one tertiary carbon. The above-mentioned characteristics of **4** showed great similarity with those of **1**–**3**, except for significant differences in the side chain at C-3. The sequential ^1^H-^1^H COSY correlations of H_3_-2/H-20/H_2_-19/H-18/H-17/H_2_-16, HMBC correlations of C-3/H_2_-16, H-17, and the deshielded chemical shift of CH-20 (*δ*_H/C_ 3.76/67.0) allowed the establishment of a 2-hexenol moiety attached at C-3 in **4** instead of an ethyl group in **1**–**3**.

Both Δ^5^ and Δ^16^ double bonds were determined as *E* configurations on the basis of NOESY correlations of 1-NH/H-6, H_2_-16/H-18, and H-17/H_2_-19. Additionally, the NOESY correlation of H-10/H_3_-15 illustrated that both were co-facial in the pyranone ring. Many initial attempts such as using Mosher’s method to assign the C-20 absolute configuration of **4** failed, meanwhile more exhaustive efforts were hampered by the limited quantity. With this in mind, four possible isomers of **4** were alternatively performed on ECD calculations. Considering the free-rotational side chain away from the main chromophore of the pyrrolidine-2,4-dione moiety, truncated structures of **4** were applied for conformational searches (Supplementary Figure S38). ECD calculations showed that the calculated ECD curve of (3*S*, 10*S*, 12*S*)-**4** was in good accordance with the experimental one, leading to the assignment of 3*S*, 10*S*, 12*S* configurations in **4**.

The relative and absolute configurations of these compounds were proposed based on a comparison of the experimental and calculated ECD spectra, along with NOESY correlations. However, since the relative configuration was not independently established through complementary methods (e.g., a detailed NMR-based conformational analysis or X-ray crystallography), the stereochemical assignments presented here should be considered tentative for both the relative and absolute configurations. To our knowledge, compounds **1**–**4** were obtained as rare leucine-derived TAs bearing an ethyl (**1**–**3**) or hexylenic alcohol (**4**) side chain and a pyranone ring at C-3 in the 2,4-pyrrolidinedione core.

**Figure 4:**
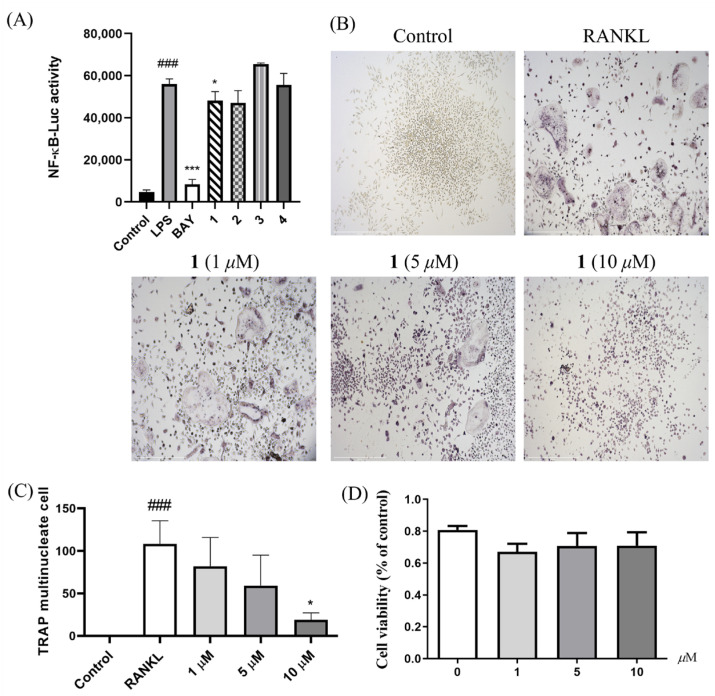
Effects of **1** on RANKL-induced osteoclastogenesis. The inhibitory effects of compounds **1**–**4** on LPS-induced NF-*κ*B activation in RAW264.7 cells at 20 μM (**A**); *n* = 3; ^###^ *p* < 0.001 vs. control group (untreated); *** *p* < 0.001, * *p* < 0.05 vs. LPS-induced group. BAY (BAY11-7082, positive control). Representative images of osteoclasts treated with **1** (1, 5, and 10 μM) for 3 days (magnification = 100×; scale bar = 500 μm) (**B**) and quantified (**C**); ^###^ *p* < 0.001 vs. control group; * *p* < 0.05 vs. RANKL group. Cell viability of **1** (1, 5, and 10 μM) in BMMs for 72 h as shown by the MTT assay (**D**).

Compounds **1**–**4** were firstly tested for their inhibitory activities of LPS-induced NF-*κ*B activation in RAW264.7 cells. Compound **1** (20 μM) showed inhibition of LPS-induced NF-*κ*B activation in RAW264.7 macrophages (*p* < 0.05) (Figure 4A). The effects of **1** on osteoclast differentiation in bone marrow macrophage cells (BMMs) and cytotoxicity were further evaluated using tartrate-resistant acid phosphatase (TRAP) and methyl thiazolyl tetrazolium (MTT) assays, respectively (Figure 4B,C). Compound **1** could suppress receptor activator of nuclear factor-*κ*B ligand (RANKL)-induced osteoclastogenisis in BMMs without obvious cytotoxicity at 10 μM (Figure 4D). To our knowledge, this work is the first example revealing TAs as potential inhibitors of osteoclast differentiation. The preliminary structure–activity relationship was discussed. Compound **1** exhibited inhibition on LPS-induced NF-*κ*B luciferase, while **2** and **3** were inactive, which revealed that chiral carbons C-10 and C-12 in the pyranone ring play eminent roles in the above bioactivity.

## 3. Materials and Methods

### 3.1. General Experimental Procedures

UV and ECD spectra were measured on a JASCO J-1500 polarimeter (JASCO Corporation, Tokyo, Japan). The NMR spectra were obtained on a Bruker Avance spectrometer (Bruker BioSpin, Fällanden, Switzerland) operating at 500 MHz for ^1^H NMR and 125 MHz for ^13^C NMR, using TMS as an internal standard. HR-ESIMS spectra were collected on a Waters Xevo G2-S TOF mass spectrometer (Waters Corporation, MA, USA). TLC and column chromatography (CC) were performed on plates precoated with silica gel GF_254_ (10–40 μm) and over silica gel (200–300 mesh) (Qingdao Marine Chemical Factory, Qingdao, China), respectively. All solvents employed were of analytical grade (Shanghai Titan Scientific Co., Ltd., Shanghai, China). Semi-preparative high-performance liquid chromatography (semi-pre HPLC) was performed on a Shimadzu SCL-10VAP system (Shimadzu, Tokyo, Japan), equipped with an ODS column (YMC-pack ODS-A, 10 mm × 250 mm, 5 μm, YMC Co., Ltd., Tokyo, Japan,) and a *π*NAP column (10 mm × 250 mm, 5 µm, COSMOSIL Co., Ltd., Tokyo, Japan,). The artificial sea salt was a commercial product (Guangzhou Haili Aquarium Technology Company, Guangzhou, China).

### 3.2. Fungal Strain and Fermentation

The strain GXIMD 02514 was isolated from the Weizhou Islands-derived coral *Pocillopora damicornis* collected in the Guangxi Zhuang autonomous region, China. It was taxonomically identified as *Montagnula* sp. GXIMD 02514 via a sequence analysis of the internal spacer (ITS) region of the rDNA (GenBank accession no. PQ349724). The voucher specimen was deposited in Guangdong Microbial Culture Collection Center (GDMCC No. 65210). The strain GXIMD 02514 was cultured on Müller–Hinton broth (MB) agar plates (15 g of malt extract, 15 g of artificial sea salt, and 20 g of agar) at 25 °C for 7 days. Then, it was inoculated in the seed medium (15 g of malt extract and 15 g of artificial sea salt in 1.0 L of tap-distilled H_2_O, at pH 7.4–7.8) at 25 °C on a rotary platform shaker at 180 rpm for 3 days. Subsequently, a large-scale fermentation of *Montagnula* sp. GXIMD 02514 was carried out in modified rice solid medium (60 g of rice, 1.4 g of artificial sea salt, 0.14 g of corn steep liquor, and 70 mL of H_2_O) employing 250 mL × 155 tissue culture bottles at room temperature for 60 days. The whole fermented cultures were extracted with EtOAc three times to provide a brown extract (197 g).

### 3.3. Extraction and Isolation

The EtOAc crude extract was fractionated by medium pressure liquid chromatography (MPLC) using a step gradient elution with petroleum ether/CH_2_Cl_2_/MeOH (petroleum ether/CH_2_Cl_2_, 1:0–0:1; CH_2_Cl_2_/methanol, 1:0–1:1, *v*/*v*), which afforded 9 fractions (Frs.1~9) based on TLC (GF_254_) properties. The HPLC-DAD analysis of these fractions revealed a series of interesting peaks in Fr.7, which were further divided into 12 subfractions (Frs.7-1~7-12) via reversed-phase MPLC with MeOH/H_2_O (10~100%). Additionally, Fr.7-8 was further separated by silica gel column chromatography using a step gradient elution with CH_2_Cl_2_/MeOH (1:0–1:1) to afford 9 subfractions (Frs.7-8-1~7-8-9). Fr.7-8-6 was purified by semipreparative high-performance liquid chromatography (HPLC) with CH_3_CN/H_2_O (45:55, *v*/*v*, 2.0 mL/min) to yield compounds **2** (*t*_R_ = 32 min, 13 mg), **4** (*t*_R_ = 53 min, 3.3 mg), and a subfraction (Fr.7-8-6-6, (*t*_R_ = 42 min). Fr. 7-8-6-6 was purified by semipreparative HPLC on a *π*NAP column with CH_3_CN/H_2_O (38:62, *v*/*v*, 2.0 mL/min) to yield compounds **1** (*t*_R_ = 38 min, 6.1 mg) and **3** (*t*_R_ = 40 min, 5.1 mg).

Montagnulan A (**1**): white powder; [α${]}_{D}^{25}$−20.8 (*c* 0.1, MeOH); UV (CH_3_OH) λmax (log *ε*) 226 (3.81), 284 (3.69) nm; ECD (0.25 mg/mL, methanol) λ_max_(∆ε) 209 (−14.0), 219 (−13.3), 265 (+0.47) nm. ^1^H NMR and ^13^C NMR data, see Table 1 and Table 2; HR-ESIMS *m*/*z* 316.1526 [M + Na]^+^ (calculated for C_16_H_23_NO_4_Na 316.1525).

Montagnulan B (**2**): white powder; [α${]}_{D}^{25}$−23.3 (*c* 0.2, MeOH); UV (CH_3_OH) λmax (log *ε*) 226 (3.82), 284 (3.68) nm; ECD (0.25 mg/mL, methanol) λ_max_(∆ε) 200 (+13.50), 215 (−25.81), 249 (+3.05), 296 (−0.42) nm. ^1^H NMR and ^13^C NMR data, see Table 1 and Table 2; HR-ESIMS *m*/*z* 316.1522 [M + Na] ^+^ (calculated for C_16_H_23_NO_4_Na 316.1525).

Montagnulan C (**3**): white powder; [α${]}_{D}^{25}$−27.8 (*c* 0.09, MeOH); UV (CH_3_OH) λmax (log *ε*) 226 (3.88), 284 (3.74) nm; ECD (0.25 mg/mL, methanol) λ_max_(∆ε) 200 (−8.07), 215 (−3.09), 226 (−8.09), 242 (−6.33), 298 (+1.44) nm. ^1^H NMR and ^13^C NMR data, see Table 1 and Table 2; HR-ESIMS *m*/*z* 316.1527 [M + Na] ^+^ (calculated for C_16_H_23_NO_4_Na 316.1525).

Montagnulan D (**4**): white powder; [α${]}_{D}^{25}$−12.0 (*c* 0.2, MeOH); UV (CH_3_OH) λmax (log *ε*) 225 (3.82), 289 (3.55) nm; ECD (0.25 mg/mL, methanol) λ_max_(∆ε) 200 (+7.99), 217 (−21.18), 290 (+0.23) nm. ^1^H NMR and ^13^C NMR data, see Table 1 and Table 2; HR-ESIMS *m*/*z* 386.1944 [M + Na] ^+^ (calculated for C_20_H_29_NO_5_Na 386.1943).

### 3.4. ECD Calculations

The theoretical ECD spectra of **1**–**4** were calculated using Gaussian 16 software according to our previously reported method [12,17,18]. Briefly, conformational searches were performed using Spartan’14 software employing a Merck molecular force field (MMFF) [20,21]. Low-energy conformers with a Boltzmann distribution over 1% were chosen for ECD calculations at the B3LYP/6-311+G (d, p) level in methanol by adopting 50 excited states. The ECD curves were generated by SpecDis 1.71 using a half band width of 0.3 eV and shifted by +9 nm (**1**, **3**, and **4**) or −8 nm (**2**) to facilitate comparison with the experimental data.

### 3.5. Anti-Osteoclastogenic Assay

The inhibition of LPS-induced NF-*κ*B activation in RAW264.7 cells of compounds **1**–**4** (20 μM) and further inhibition of osteoclastogenesis of BMMs by **1** (1, 5, and 10 μM) were carried out as described previously [12,15,16]. BAY11-7082 (5 μM, Sigma-Aldrich), a known NF-*κ*B inhibitor, was used as the positive control. Meanwhile, the cytotoxicity of **1** on BMMs was evaluated using a CCK-8 kit. Statistical differences among groups were assessed using a one-way analysis of variance (ANOVA) with a Bonferroni post hoc test. A *p*-value of < 0.05 was considered statistically significant.

## 4. Conclusions

Four novel TA derivatives were obtained from the Beibu Gulf coral-associated fungus *Montagnula* sp. GXIMD 02514. To our knowledge, compounds **1**–**4** were characterized as rare leucine-derived TAs harboring an ethyl (**1**–**3**) or hexylenic alcohol (**4**) side chain and a pyranone ring at C-3 in the 2,4-pyrrolidinedione core. Montagnulan A (**1**) exhibited inhibition of LPS-induced NF-*κ*B in RAW 264.7 macrophages at 20 μM, which further suppressed RANKL-induced osteoclast differentiation without cytotoxicity in BMMs. This is the first report of osteoclastogenesis inhibitory activity for TAs. Collectively, our findings expand the chemical space and biological diversity of TAs, and highlight the need for further drug development of montagnulan A (**1**) as a novel potential inhibitor of osteoclast differentiation.

## Figures and Tables

**Table 1:** ^1^H NMR (500 MHz) data of compounds **1**–**4** in CDCl_3_ (*δ*_H_ in ppm, *J* in Hz).

No.	1	2	3	4
1	8.66, s	9.56, s	8.51, s	8.50, s
6	5.61, d (10.0)	5.60, d (10.0)	5.62, d (10.0)	5.31, d (10.2)
7	2.45, m	2.52, m	2.45, m	3.56, m
8	1.12, d (7.5)	1.09, d (7.5)	1.11, d (7.5)	1.04, d (7.0)
9	1.10, d (7.5)	1.09, d (7.5)	1.11, d (7.5)	1.02, d (7.0)
10	2.55, m	2.54, m	2.43, m	2.54, m
11	1.98, ddd (14.0, 6.5, 4.5) 1.75, dt (14.0, 8.5)	1.94, ddd (14.0, 7.0, 4.5) 1.69, dt (14.0, 8.0)	1.78, q (7.5) 1.50, q (12.5)	1.68, dt (14.4, 8.5) 1.84, ddd (14.4, 6.8, 4.3)
12	4.46, m	4.46, m	4.34, m	4.47, m
14	2.62, m 2.52, m	2.68, dd (15.5, 12.5) 2.61, dd (15.5, 10.0)	2.70, m	2.62, dd (15.6, 5.5) 2.80, dd (15.6, 12.5)
15	1.34, d (6.0)	1.34, d (7.5)	1.36, d (6.0)	1.34, d (6.3)
16	1.84, q (7.5)	1.83, q (7.5)	1.84, q (7.5)	2.48, m
17	0.84, t (7.5)	0.83, t (7.5)	0.84, t (7.5)	5.32, m
18				5.55, m
19				2.16, dt (14.0, 5.0) 2.02, dt (14.0, 8.0)
20				3.76, m
21				1.14, (6.2)

**Table 2:** ^13^C NMR (125 MHz) data of compounds **1**–**4** in CDCl_3_ (*δ*_H_ in ppm, type).

No.	1	2	3	4
2	174.7, C	175.6, C	174.5, C	173.5, C
3	57.1, C	56.9, C	56.9, C	57.9, C
4	200.0, C	200.0, C	199.9, C	200.4, C
5	132.0, C	132.3, C	132.1, C	130.8, C
6	119.1, CH	119.7, CH	119.0, CH	126.5, CH
7	27.1, CH	27.0, CH	27.8, CH	25.6, CH
8	22.2, CH_3_	22.2, CH_3_	22.2, CH_3_	23.2, CH_3_
9	22.2, CH_3_	22.2, CH_3_	22.2, CH_3_	23.1, CH_3_
10	33.2, CH	32.9, CH	36.4, CH	32.6, CH
11	30.3, CH_2_	30.2, CH_2_	31.9, CH_2_	30.5, CH_2_
12	73.7, CH	73.8, CH	76.6, CH	73.6, CH
13	171.7, C	171.9, C	170.0, C	171.9, C
14	30.5, CH_2_	30.5, CH_2_	30.8, CH_2_	30.3, CH_2_
15	20.8, CH_3_	20.9, CH_3_	21.9, CH_3_	20.9, CH_3_
16	26.0, CH_2_	25.9, CH_2_	25.5, CH_2_	36.0, CH_2_
17	8.7, CH_3_	8.7, CH_3_	8.7, CH_3_	125.2, CH
18				133.2, CH
19				42.5, CH_2_
20				67.0, CH
21				22.8, CH_3_

## Data Availability

The original data presented in the study are included in the article and Supplementary Material; further inquiries can be directed to the corresponding author.

## Supplementary Materials

The following supporting information can be downloaded at https://www.mdpi.com/article/10.3390/md23110416/s1, The ^1^D and ^2^D NMR, HR-ESIMS, and UV spectra of compounds **1**–**4** (Supplementary Figures S1–S33), the calculated ECD data of **1**–**4** (Supplementary Figure S34–S39 and Tables S1–S8), and *Montagnula* sp. GXIMD 02514.
