# Supplementary material for: Montagnulans A–D with Anti-Osteoclastogenic Activity from the Marine Fungus Montagnula sp. GXIMD 02514
Source: Mar Drugs. 2025 Oct 25;23(11):416. doi: 10.3390/md23110416 (PMC12654181; doi:10.3390/md23110416)
Supplement: Supplementary file 1 [file marinedrugs-23-00416-s001.zip › marinedrugs-3922979-supplementary.pdf]

Supplementary Materials

Contents

Figure S1. ^{1}H NMR spectrum of montagnulan A (**1**) ($CDCl_3$, 500 MHz)..3
Figure S2. ^{13}C NMR spectrum of montagnulan A (**1**) ($CDCl_3$, 125 MHz)......................................4
Figure S3. HSQC spectrum of montagnulan A (**1**) ($CDCl_3$)...5
Figure S4. HMBC spectrum of montagnulan A (**1**) ($CDCl_3$). 6
Figure S5. ^{1}H-^{1}H COSY spectrum of montagnulan A (**1**) ($CDCl_3$)..8
Figure S6. NOESY spectrum of montagnulan A (**1**) ($CDCl_3$). 9
Figure S7. HR-ESIMS spectrum of montagnulan A (**1**)...9
Figure S8. UV spectrum of montagnulan A (**1**)...10
Figure S9. ^{1}H NMR spectrum of montagnulan B (**2**) ($CDCl_3$, 500 MHz). 11
Figure S10. ^{13}C NMR spectrum of montagnulan B (**2**) ($CDCl_3$, 125 MHz). 12
Figure S11. DEPT135 NMR spectrum of montagnulan B (**2**) (CDCl3). 13
Figure S12. HSQC spectrum of montagnulan B (**2**) ($CDCl_3$). 14
Figure S13. HMBC spectrum of montagnulan B (**2**) ($CDCl_3$). 15

Figure S14. ^{1}H-^{1}H COSY spectrum of montagnulan B (**2**) ($CDCl_3$)....16
Figure S15. NOESY spectrum of montagnulan B (**2**) ($CDCl_3$)....17
Figure S16. HR-ESIMS spectrum of montagnulan B (**2**)....18
Figure S17. UV spectrum of montagnulan B (**2**). 19
Figure S18. ^{1}H NMR spectrum of montagnulan C (**3**) ($CDCl_3$, 500 MHz). 20
Figure S19. ^{13}C NMR spectrum of montagnulan C (**3**) ($CDCl_3$, 125 MHz). 21
Figure S20. HSQC spectrum of montagnulan C (**3**) ($CDCl_3$). 22
Figure S21. HMBC spectrum of montagnulan C (**3**) ($CDCl_3$). 23
Figure S22. ^{1}H-^{1}H COSY spectrum of montagnulan C (**3**) ($CDCl_3$)....24
Figure S23. NOESY spectrum of montagnulan C (**3**) ($CDCl_3$)....25
Figure S24. HR-ESIMS spectrum of montagnulan C (**3**)....26
Figure S25. UV spectrum of montagnulan C (**3**). 27
Figure S26. ^{1}H NMR spectrum of montagnulan D (**4**). ($CDCl_3$, 500 MHz). 28
Figure S27. ^{13}C NMR spectrum of montagnulan D (**4**) ($CDCl_3$, 125 MHz). 29
Figure S28. HSQC spectrum of montagnulan D (**4**) ($CDCl_3$). 30
Figure S29. HMBC spectrum of montagnulan D (**4**) ($CDCl_3$). 31
Figure S30. ^{1}H-^{1}H COSY spectrum of montagnulan D (**4**) ($CDCl_3$). 32
Figure S31. NOESY spectrum of montagnulan D (**4**) ($CDCl_3$)....33
Figure S32. HR-ESIMS spectrum of montagnulan D (**4**). 34
Figure S33. UV spectrum of montagnulan D (**4**). 34
Table S1. Energies of **1** at MMFF94 force field. 35
Table S2. Energies of **1** at B3LYP/6–31+g(d) level in methanol....35
Figure S34. The optimized conformers and equilibrium populations of **1**. 40
Table S3. Energies of **2** at MMFF94 force field. 40
Table S4. Energies of **2** at B3LYP/6–31+g(d) level in methanol....41
Figure S35. The optimized conformers and equilibrium populations of **2**. 42
Table S5. Energies of **3** at MMFF94 force field. 42
Table S6. Energies of **3** at B3LYP/6–31+g(d) level in methanol....43
Figure S36. The optimized conformers and equilibrium populations of **3**. 49
Figure S37. Structures of four possible isomers of **1** for ECD calculations. 50
Figure S38. Truncated structures of four possible isomers of **4** for ECD calculations....50
Table S7. Energies of model-**4** at MMFF94 force field....50
Table S8. Energies of model-**4** at B3LYP/6–31+g(d) level in methanol. 52
Figure S39. The optimized conformers and equilibrium populations of model-**4**....58
The ITS sequence of *Montagnula* sp. GXIMD 02514 58

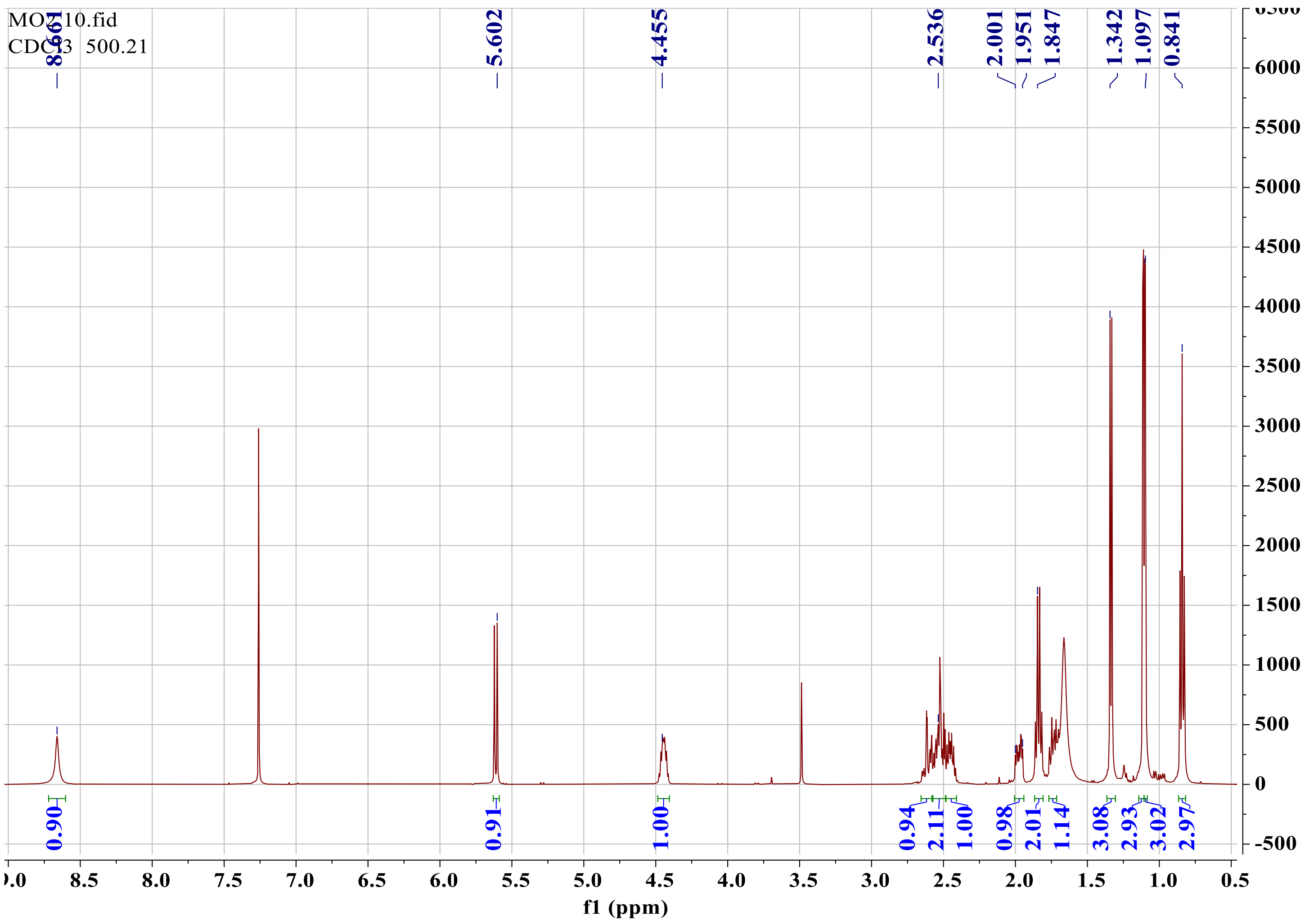

Figure S1. ^{1}H NMR spectrum of montagnulan A (**1**) ($CDCl_3$, 500 MHz).

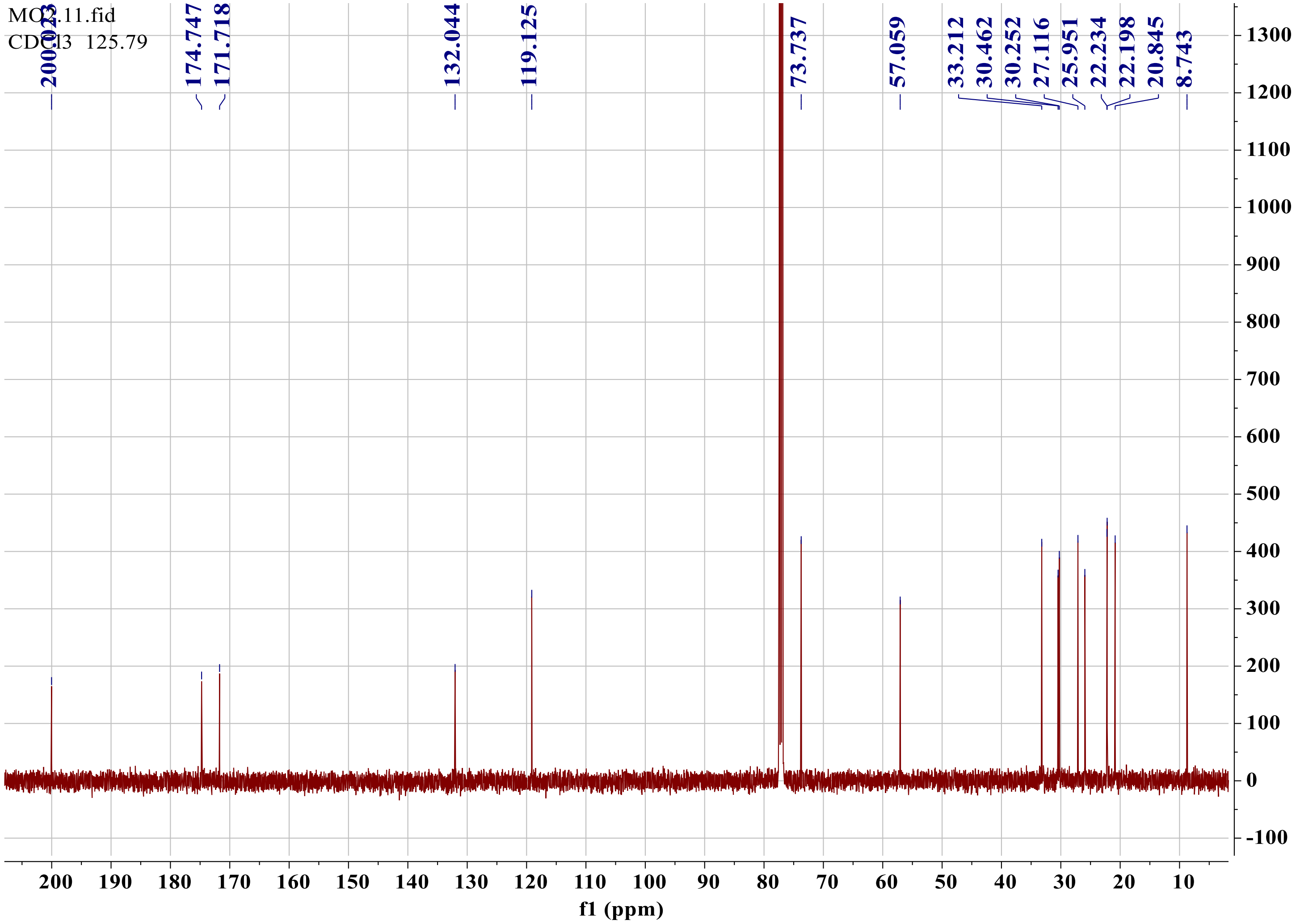

Figure S2. ^{13}C NMR spectrum of montagnulan A (**1**) ($CDCl_3$, 125 MHz).

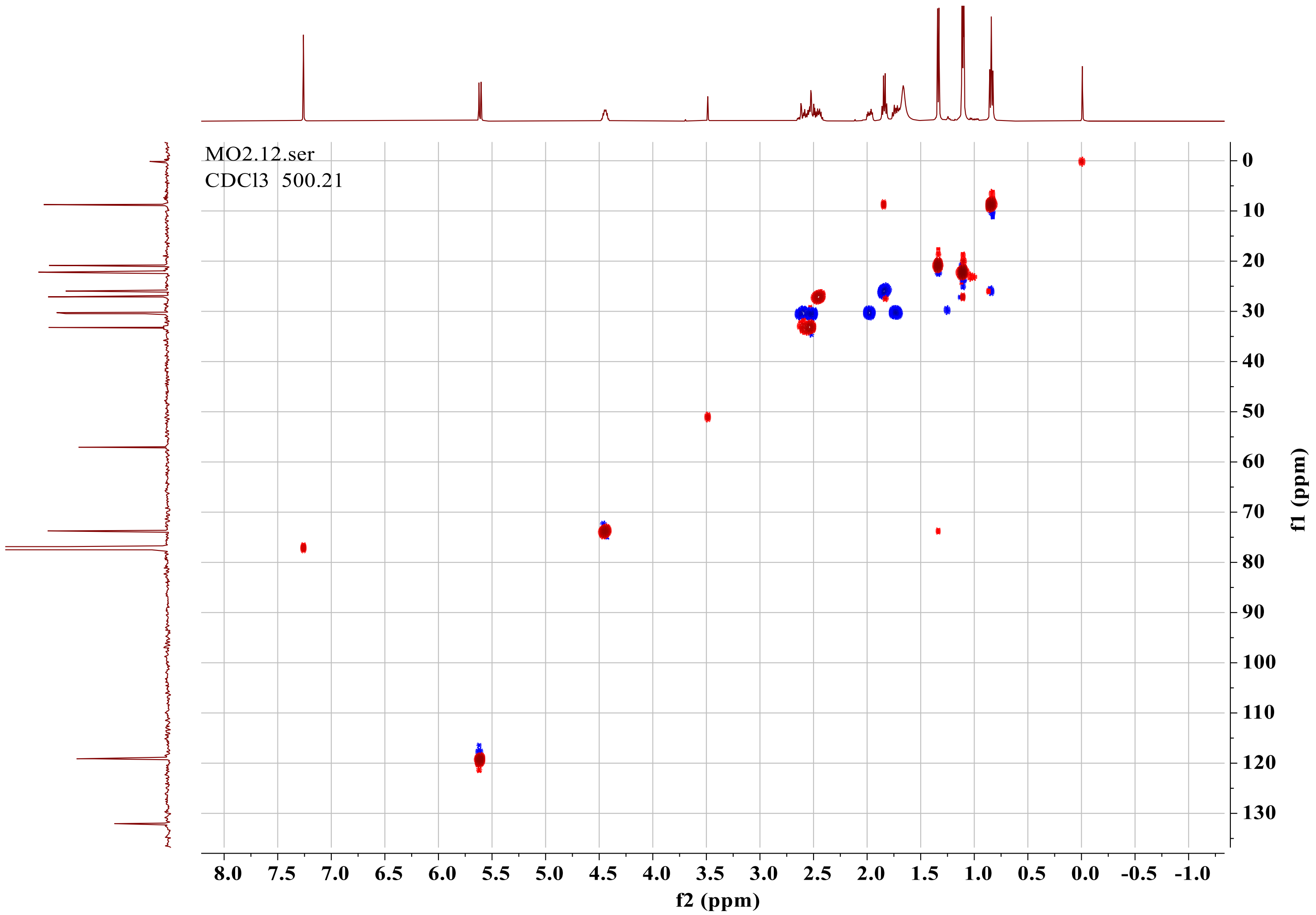

Figure S3. HSQC spectrum of montagnulan A (**1**) ($CDCl_3$).

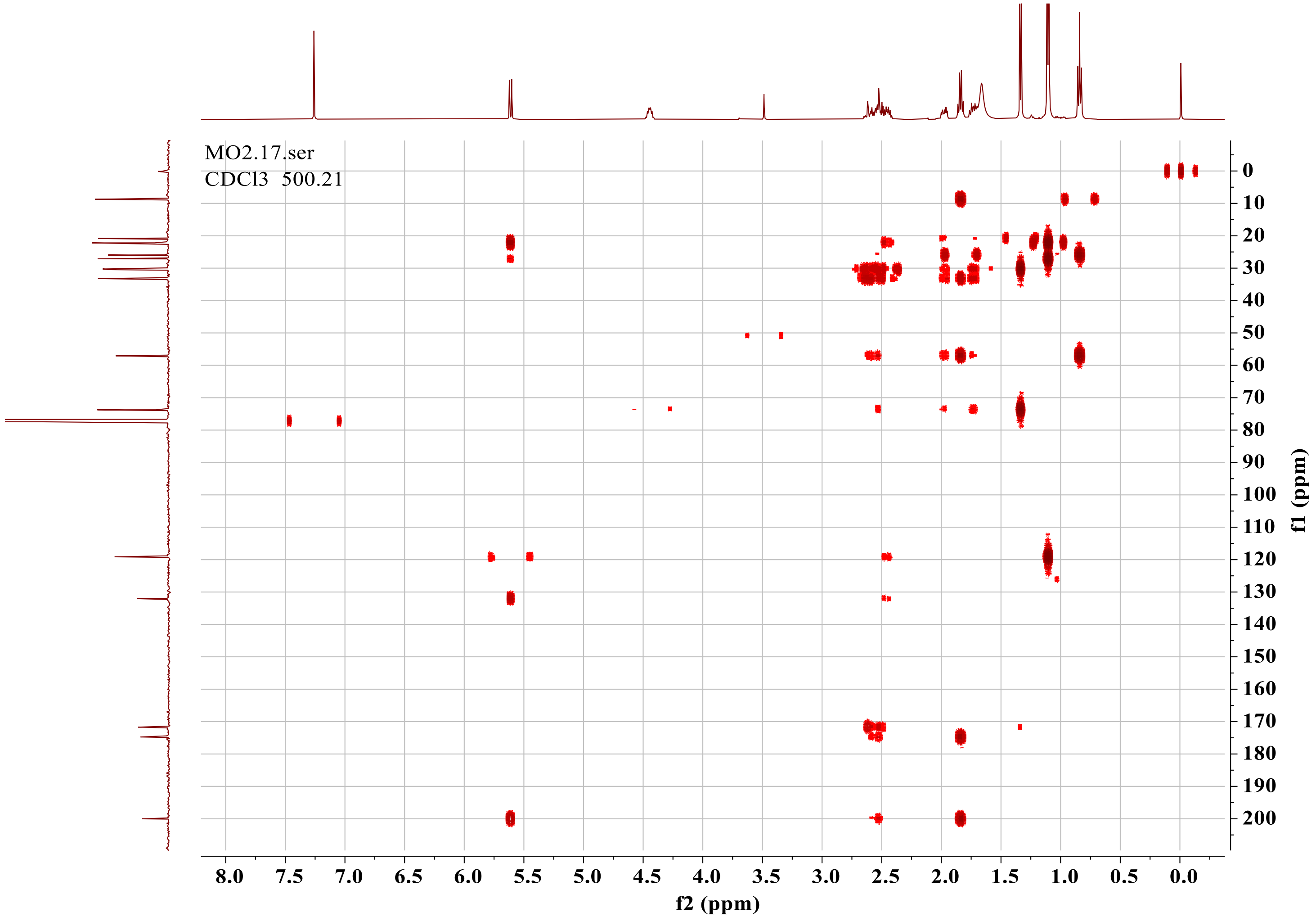

Figure S4. HMBC spectrum of montagnulan A (**1**) ($CDCl_3$).

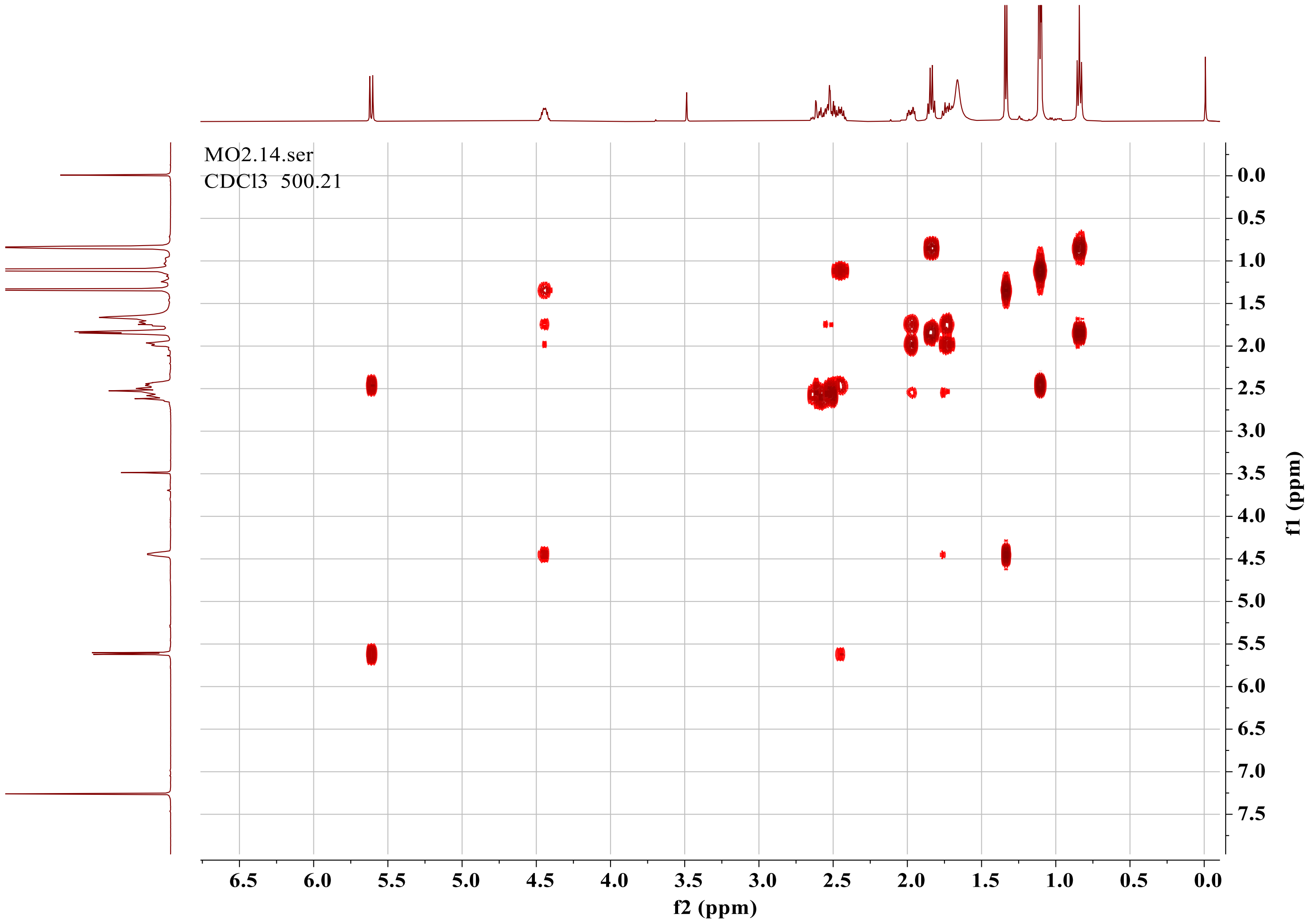
MO2.14.ser
CDCl3 500.21
f2 (ppm)
f1 (ppm)

Figure S5. ^{1}H-^{1}H COSY spectrum of montagnulan A (**1**) ($CDCl_3$).

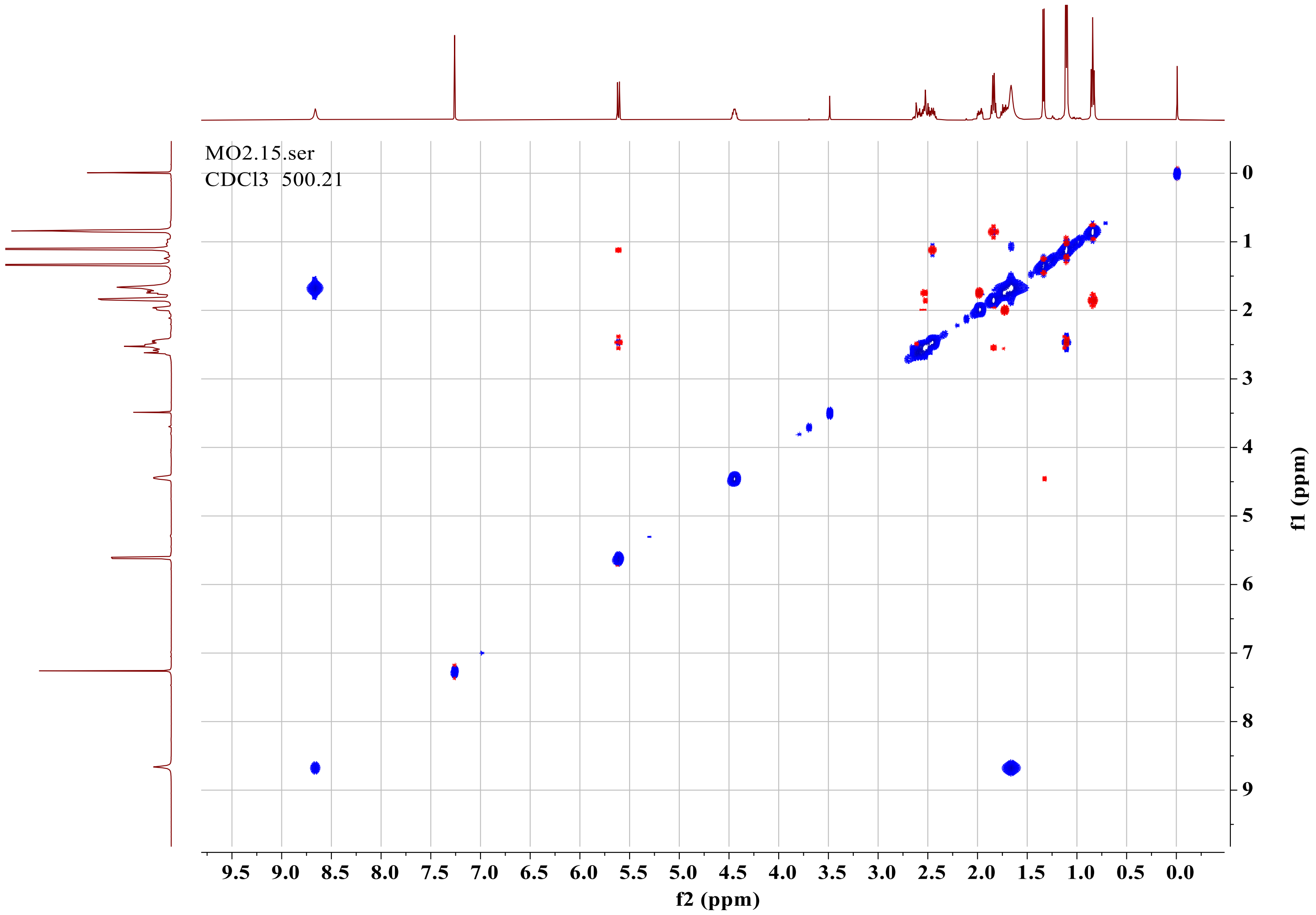

Figure S6. NOESY spectrum of montagnulan A (**1**) ($CDCl_3$).

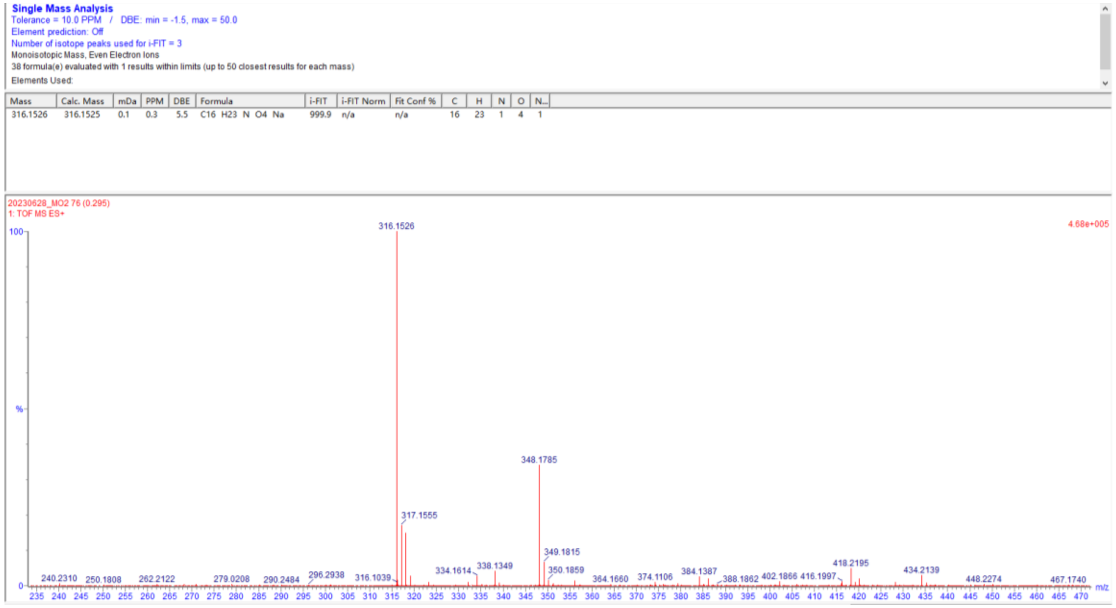

Figure S7. HR-ESIMS spectrum of montagnulan A (**1**).

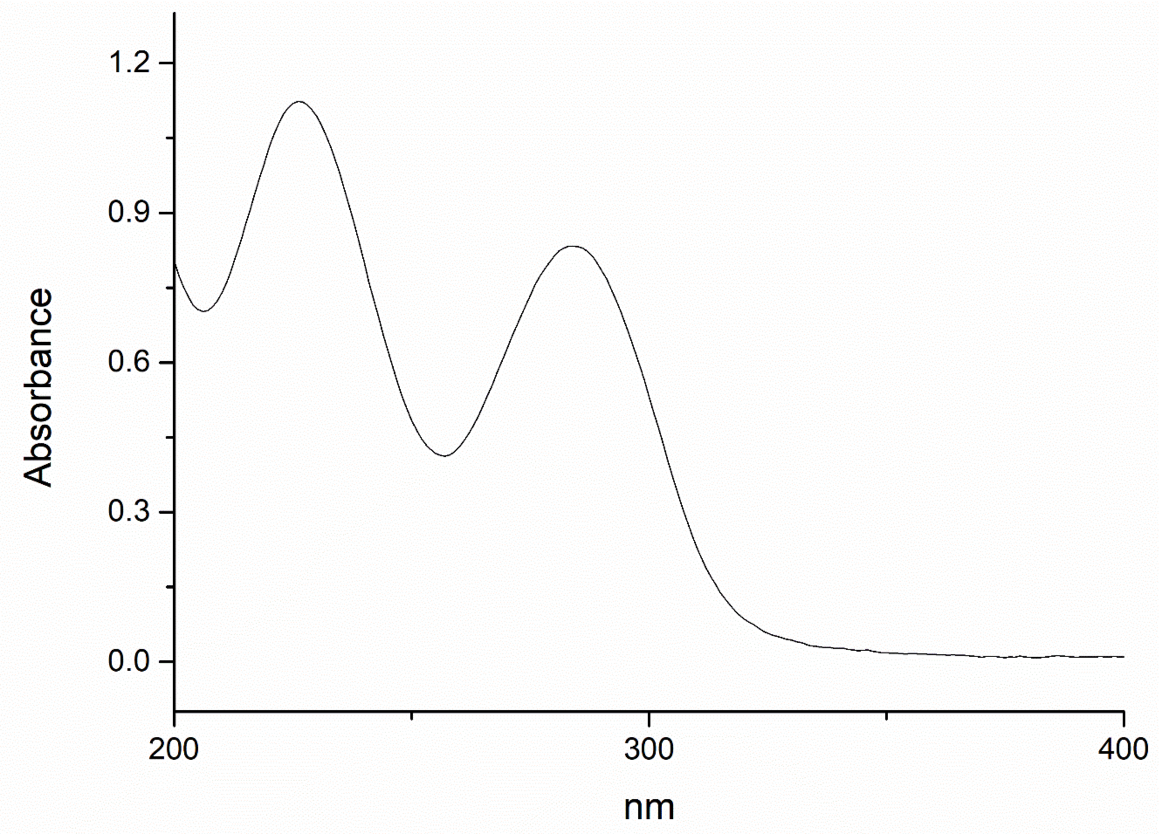

Figure S8. UV spectrum of montagnulan A (**1**).

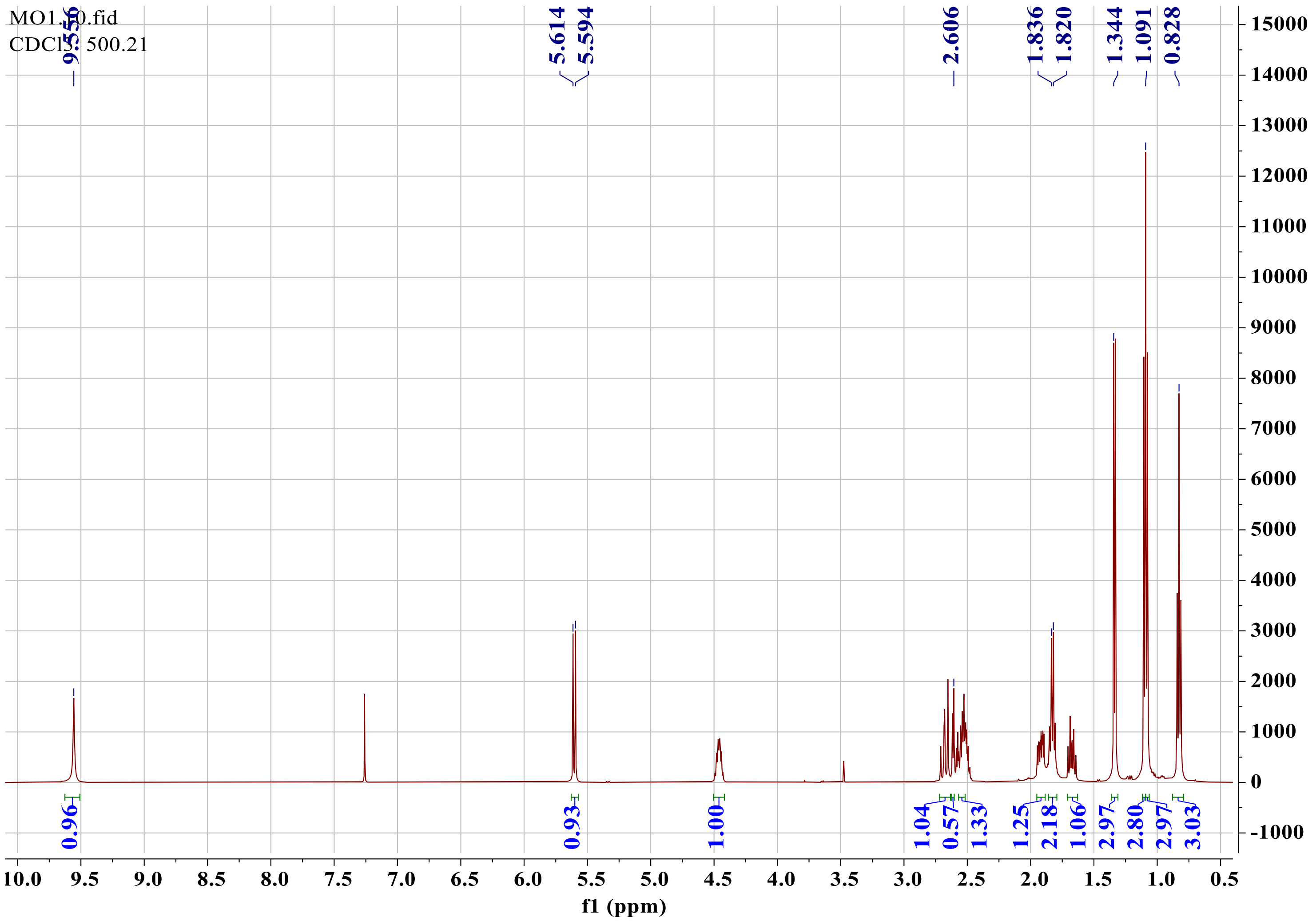

Figure S9. ^{1}H NMR spectrum of montagnulan B (**2**) ($CDCl_3$, 500 MHz).

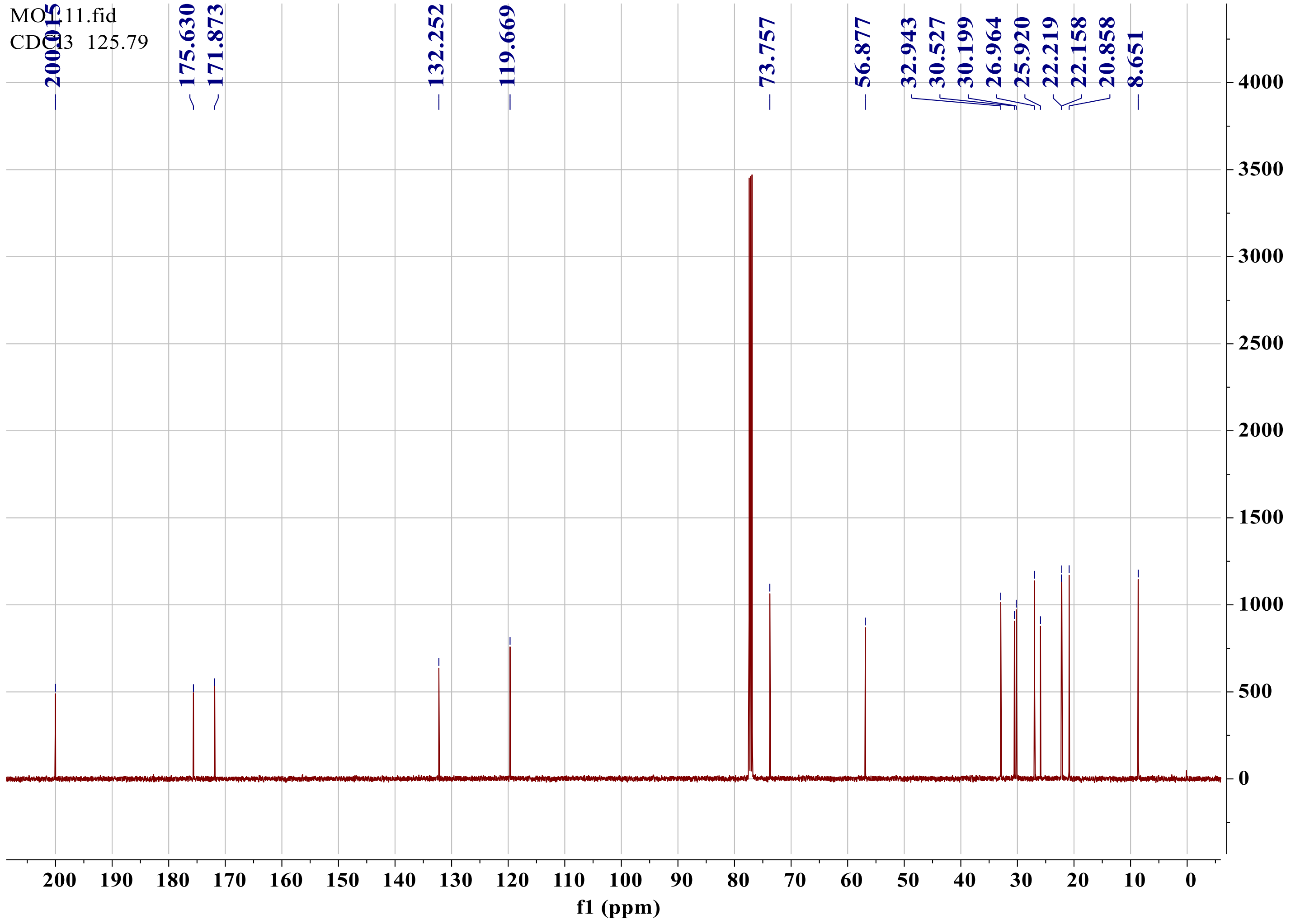

Figure S10. ^{13}C NMR spectrum of montagnulan B (**2**) ($CDCl_3$, 125 MHz).

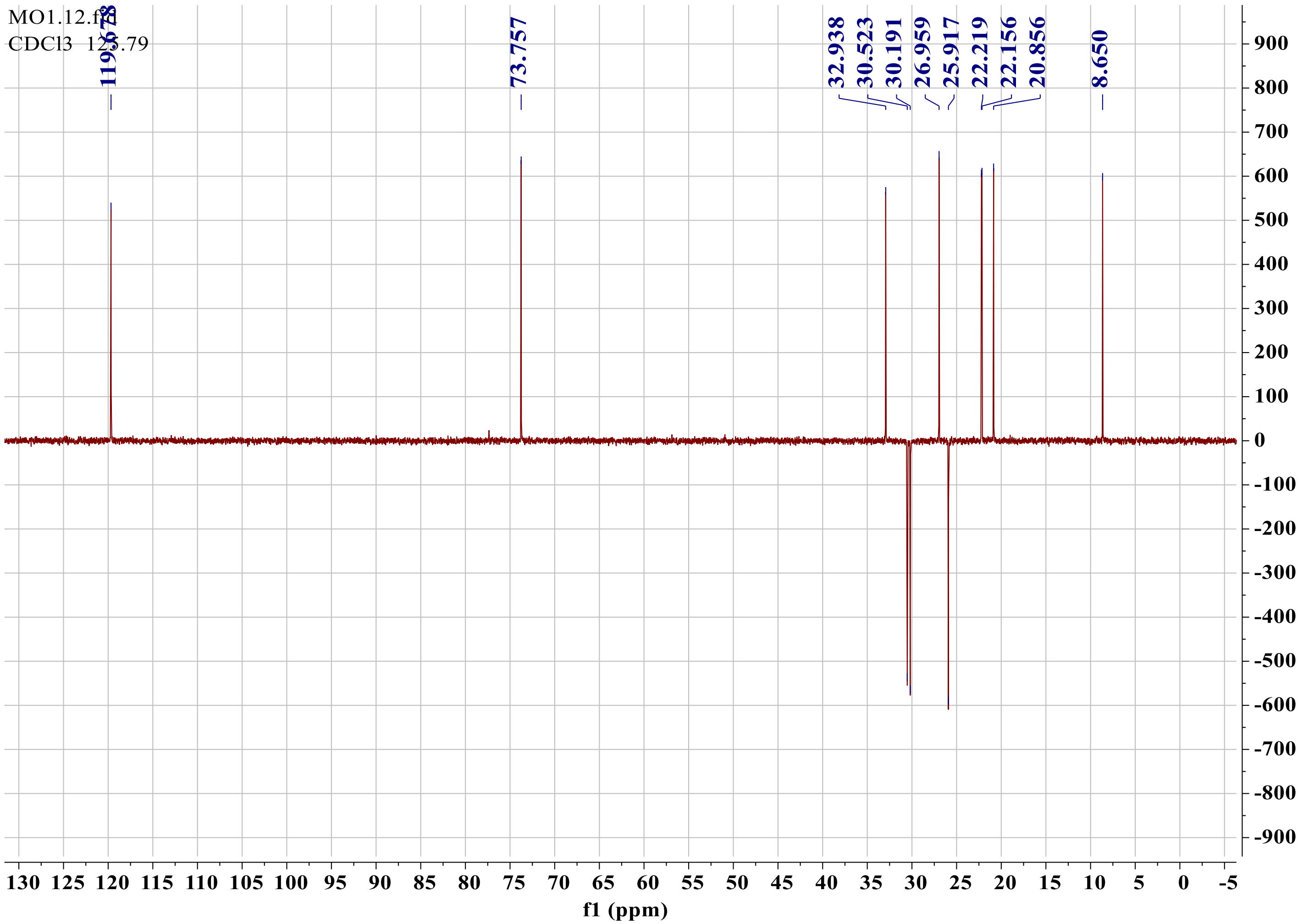

Figure S11. DEPT135 NMR spectrum of montagnulan B (**2**) (CDCl3).

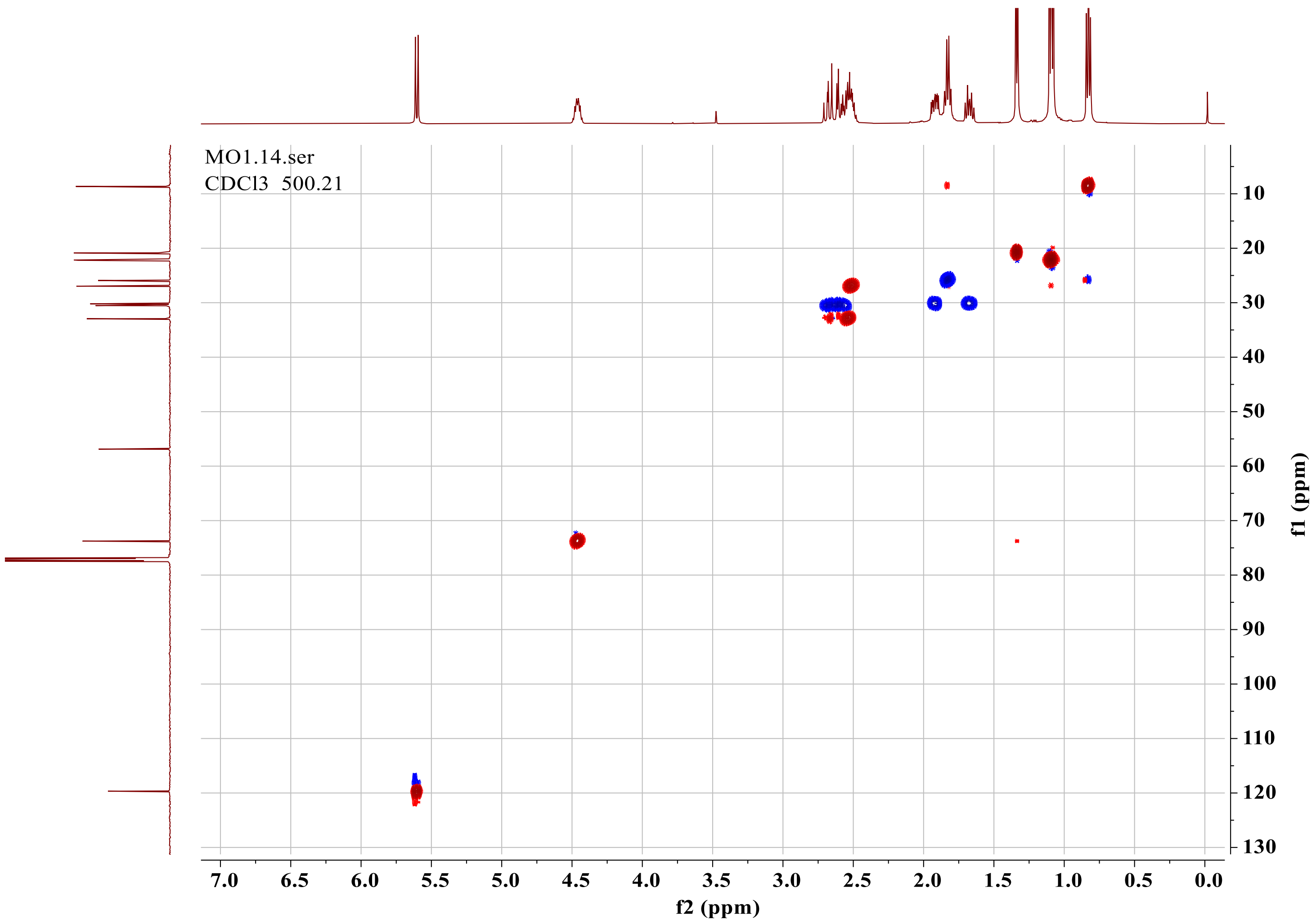

Figure S12. HSQC spectrum of montagnulan B (**2**) ($CDCl_3$).

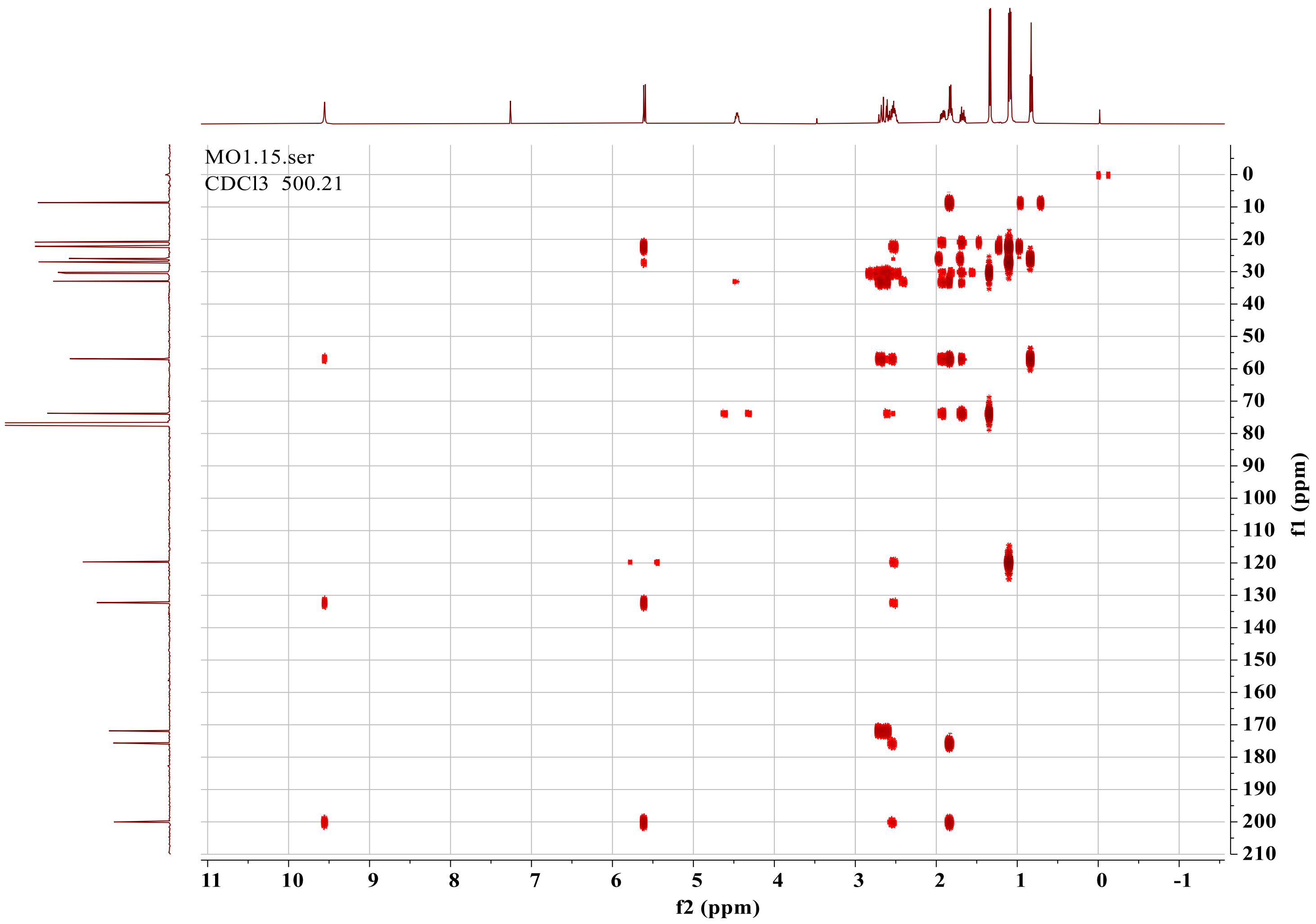

Figure S13. HMBC spectrum of montagnulan B (**2**) ($CDCl_3$).

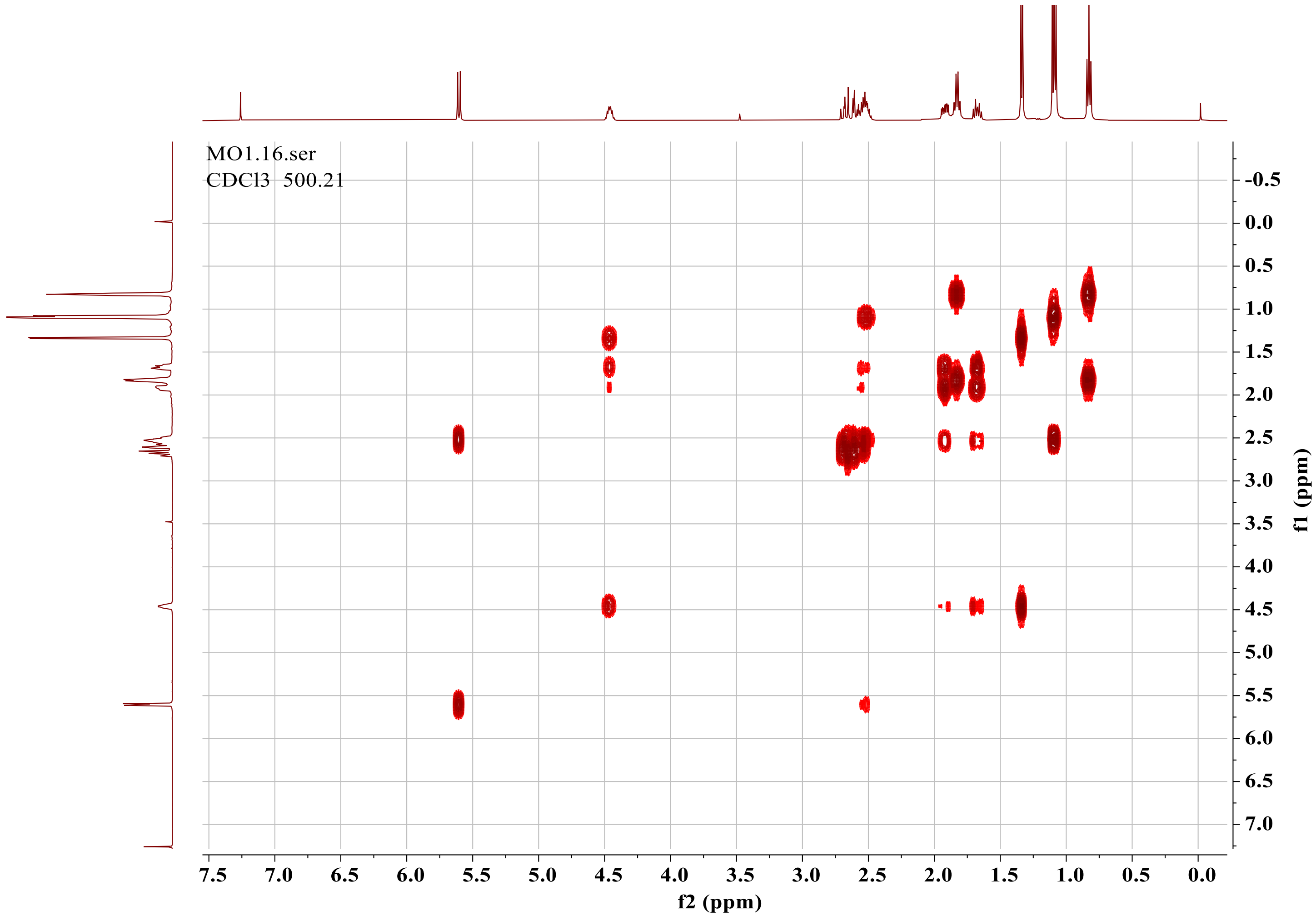

Figure S14. ^{1}H-^{1}H COSY spectrum of montagnulan B (**2**) ($CDCl_3$).

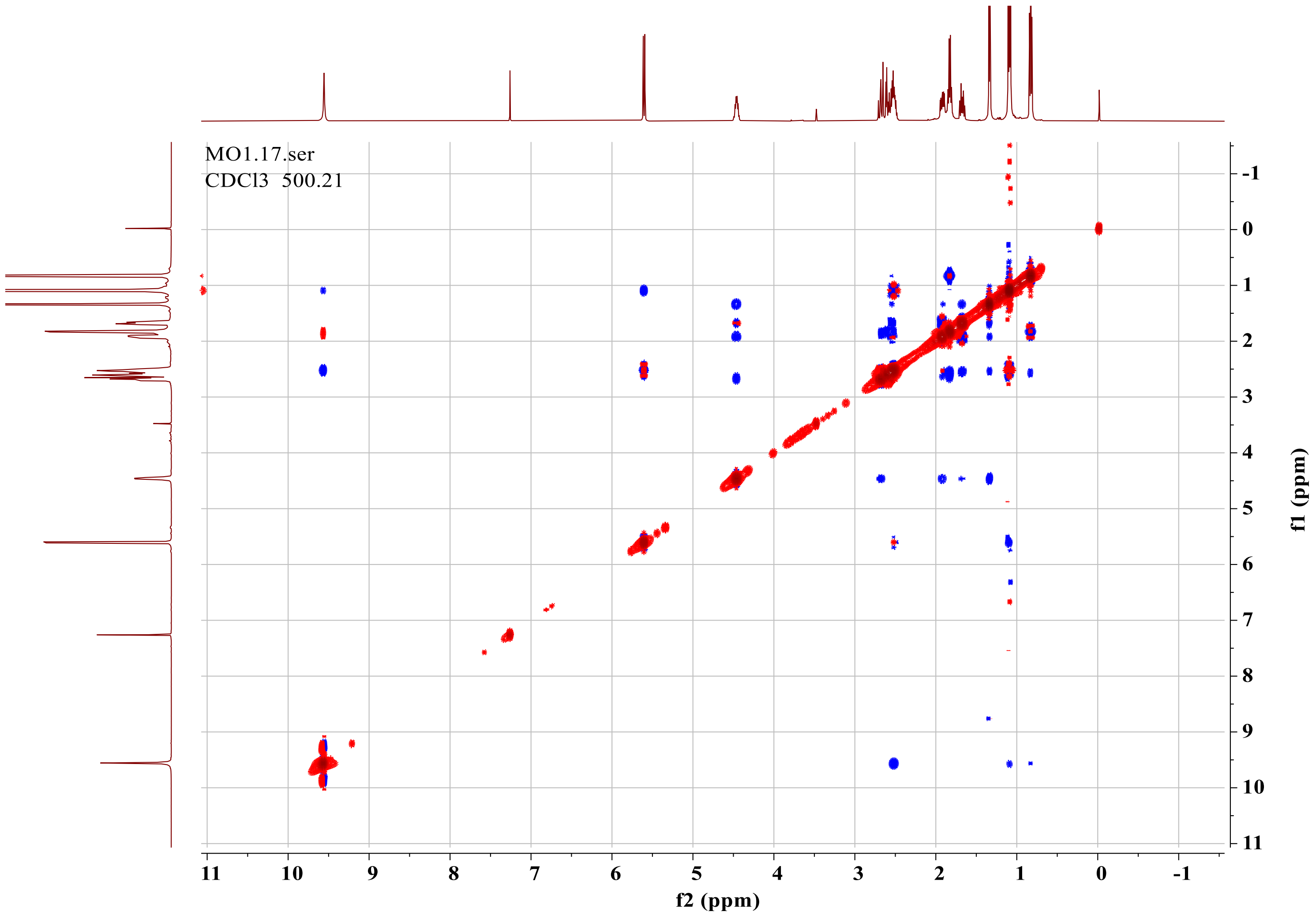

Figure S15. NOESY spectrum of montagnulan B (**2**) ($CDCl_3$).

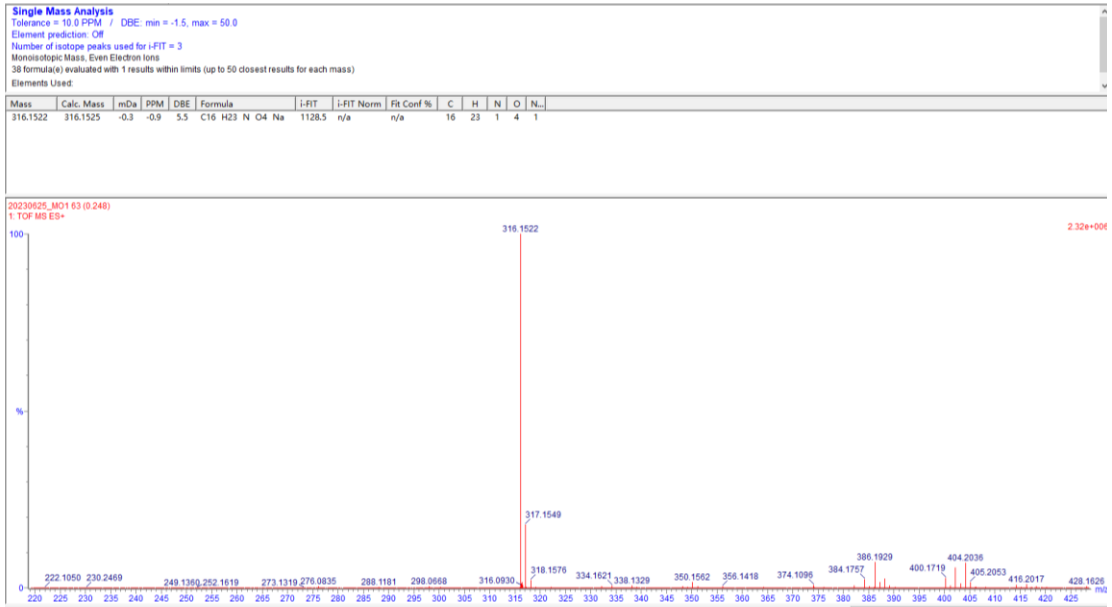

Figure S16. HR-ESIMS spectrum of montagnulan B (**2**).

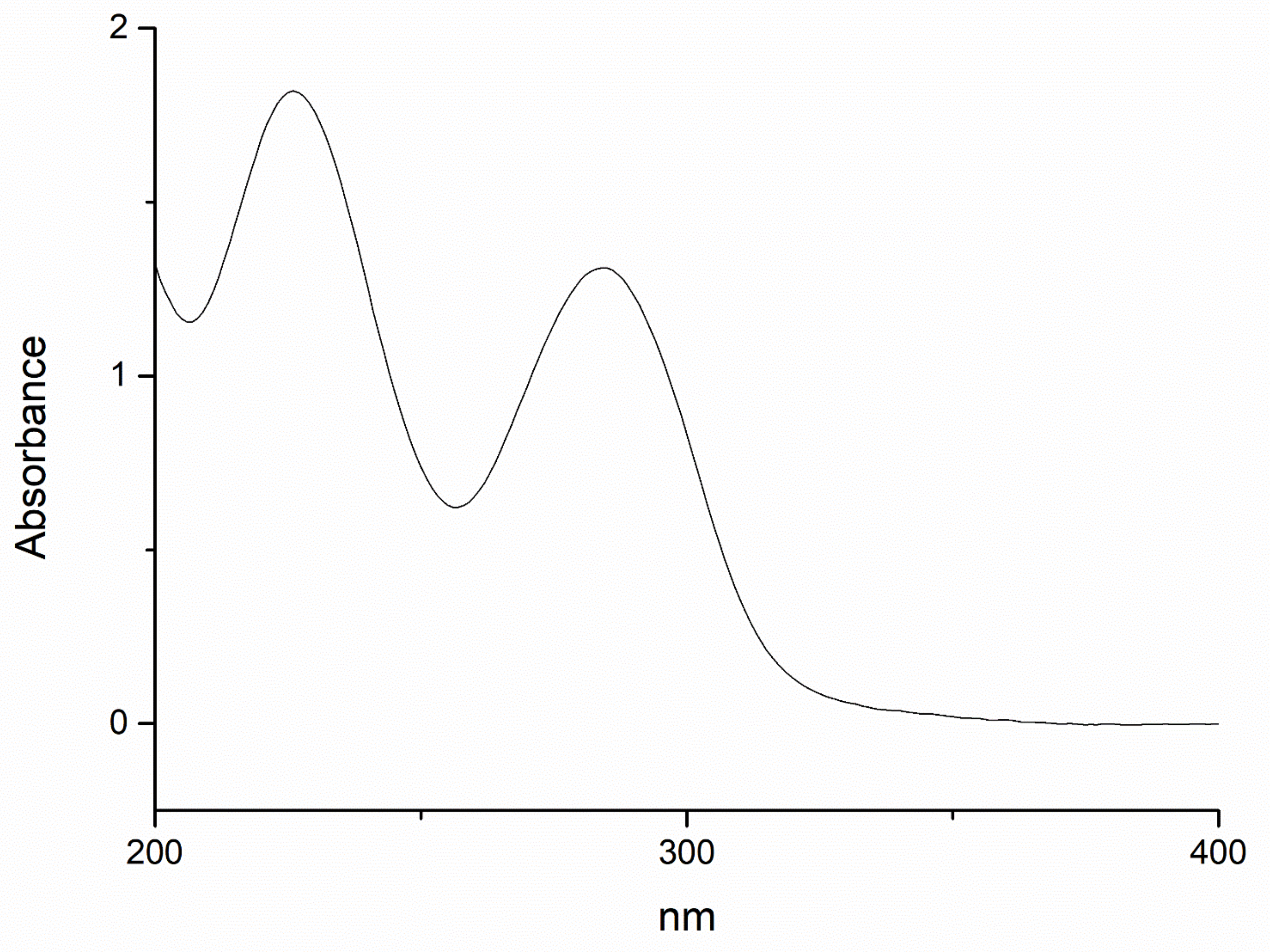

Figure S17. UV spectrum of montagnulan B (**2**).

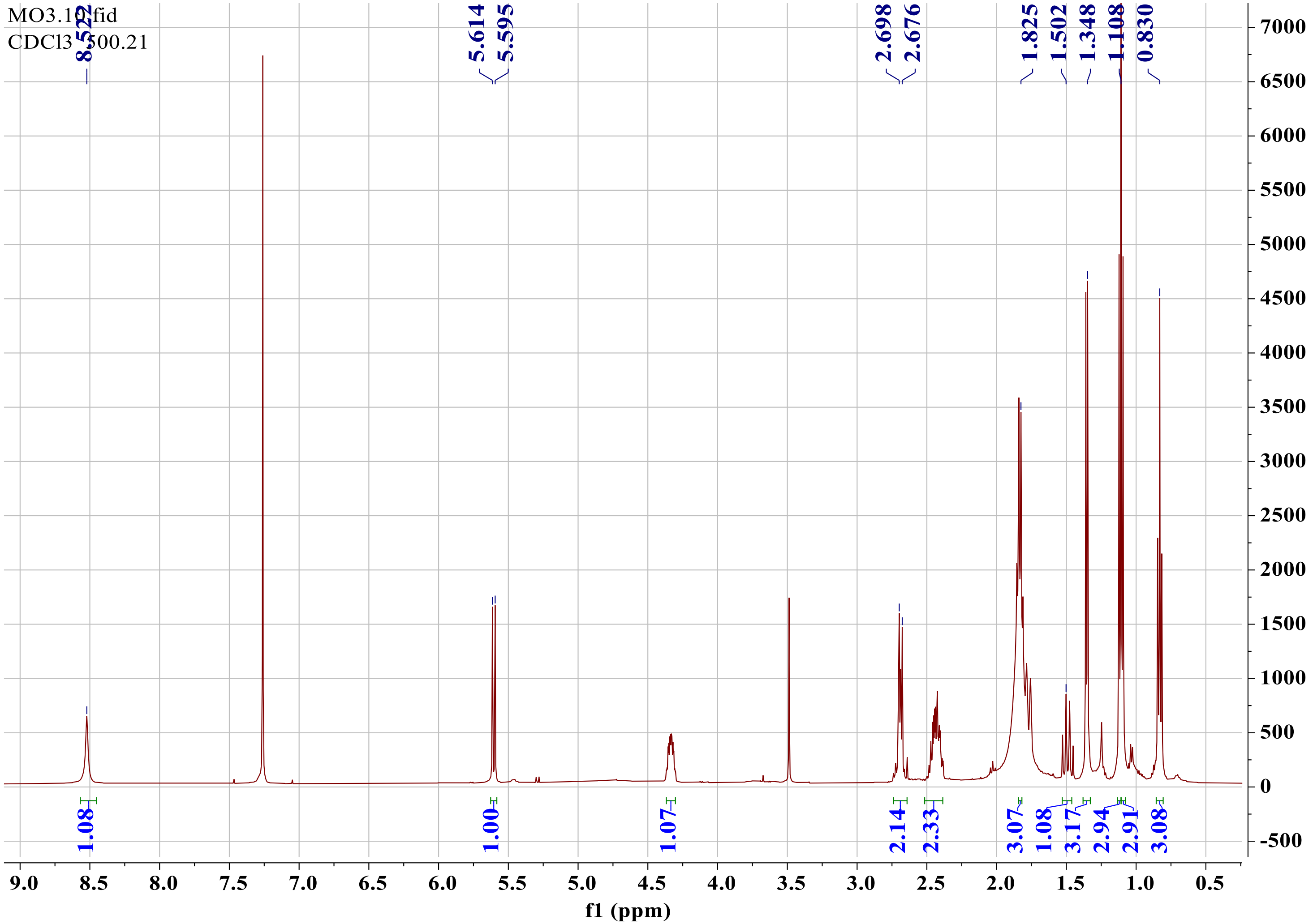

Figure S18. ^{1}H NMR spectrum of montagnulan C (**3**) ($CDCl_3$, 500 MHz).

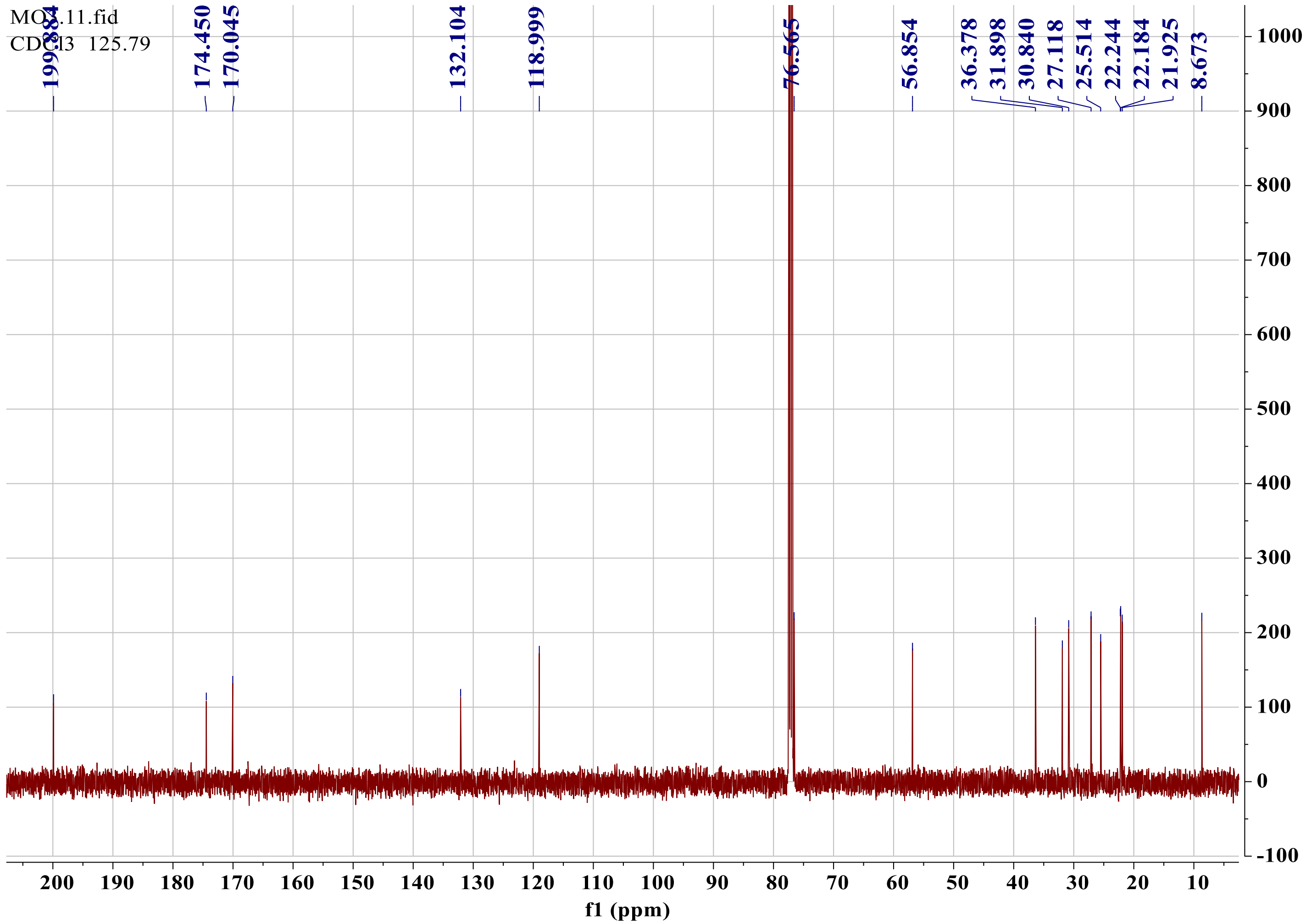

Figure S19. ^{13}C NMR spectrum of montagnulan C (**3**) ($CDCl_3$, 125 MHz).

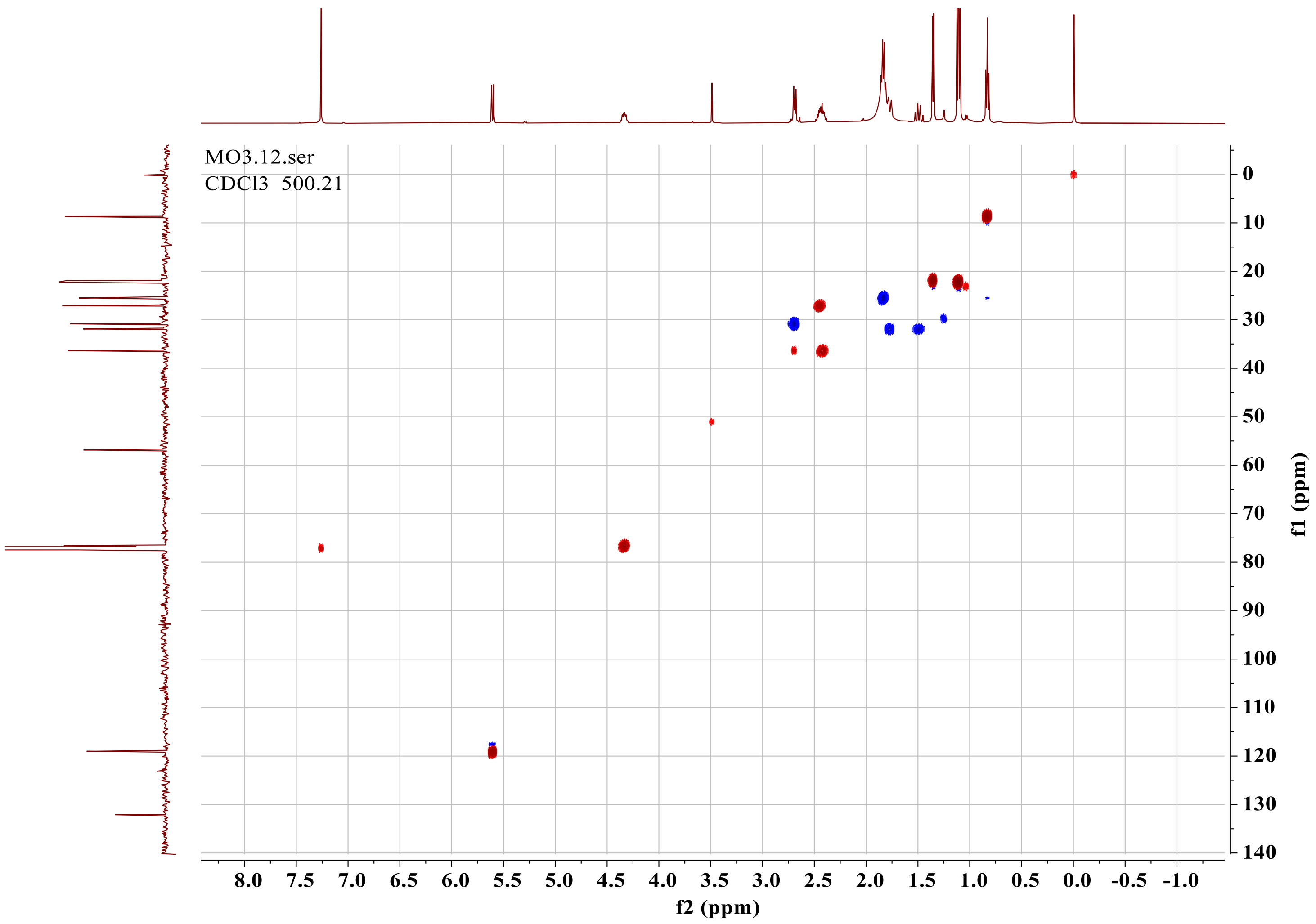

Figure S20. HSQC spectrum of montagnulan C (**3**) ($CDCl_3$).

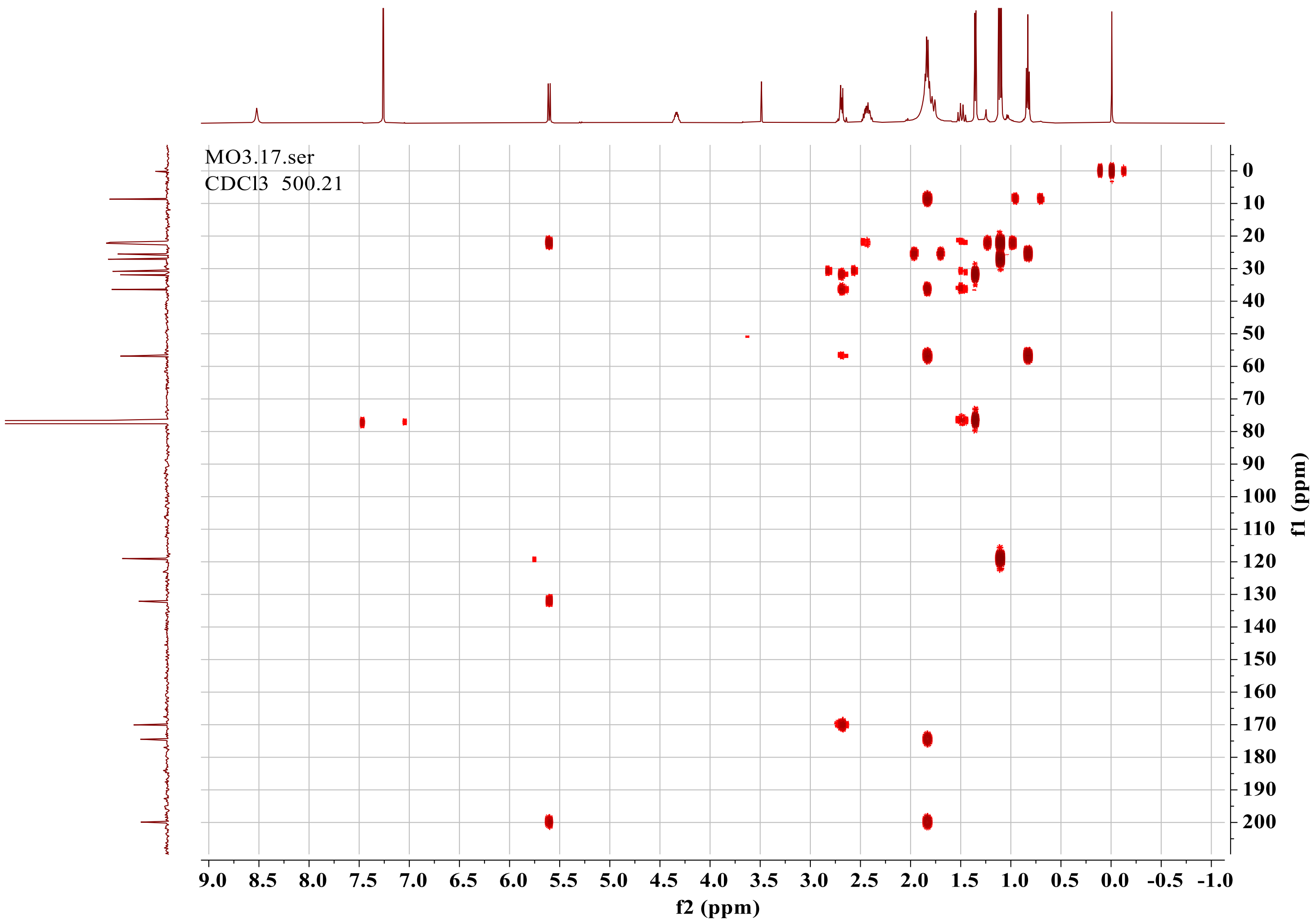

Figure S21. HMBC spectrum of montagnulan C (**3**) ($CDCl_3$).

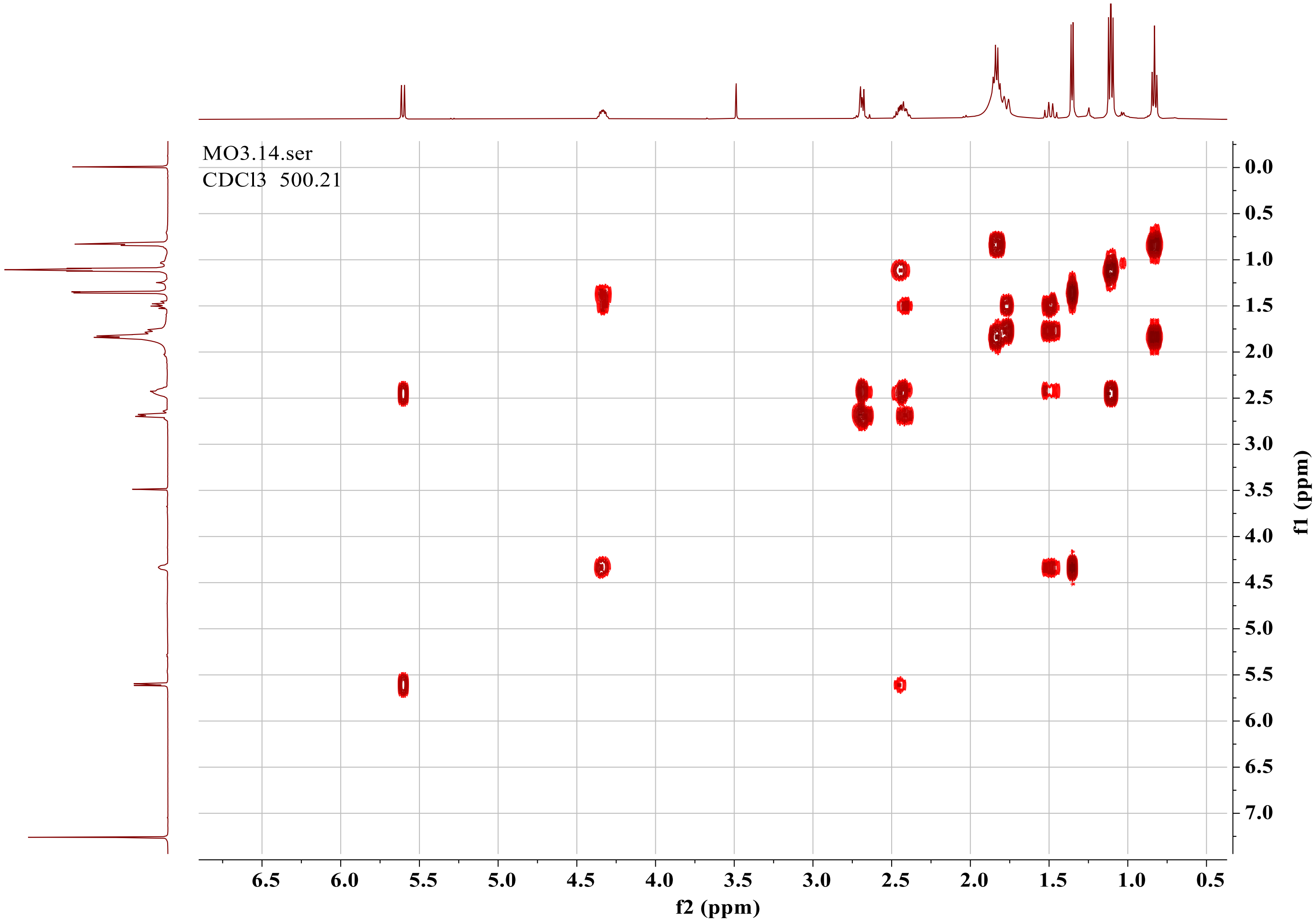

Figure S22. ^{1}H-^{1}H COSY spectrum of montagnulan C (**3**) ($CDCl_3$).

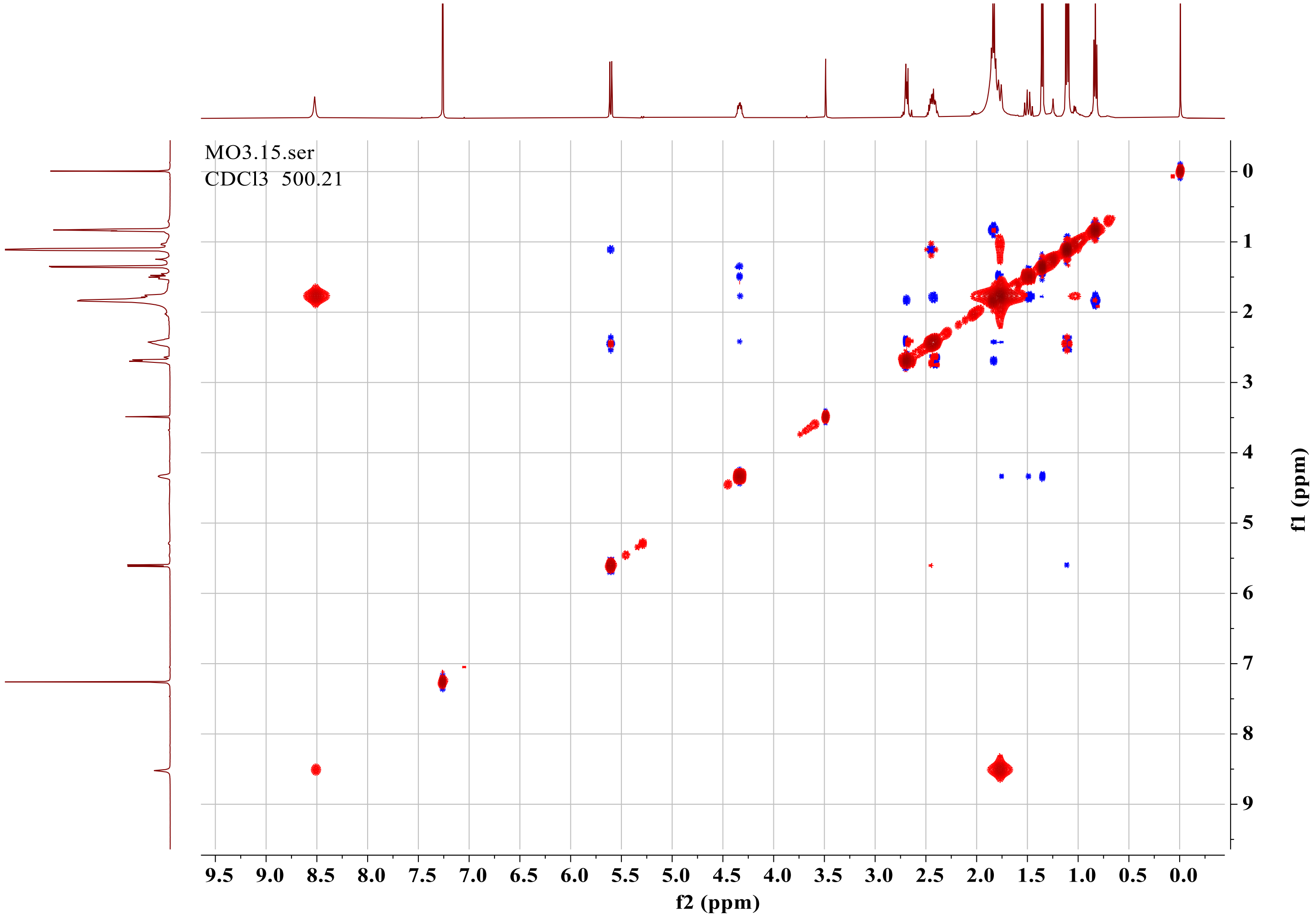

Figure S23. NOESY spectrum of montagnulan C (**3**) ($CDCl_3$).

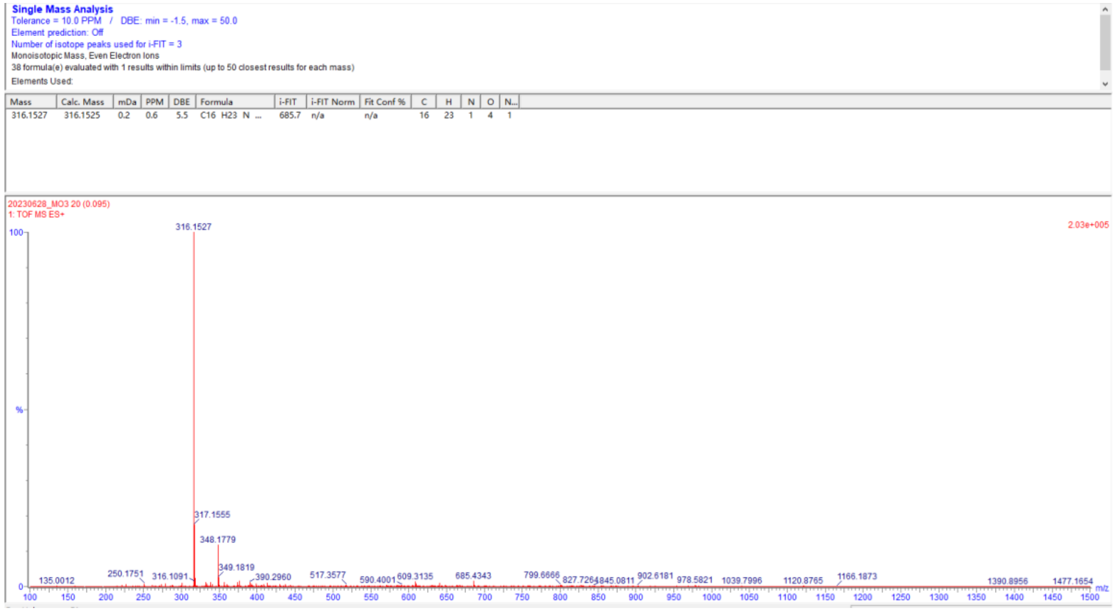

Figure S24. HR-ESIMS spectrum of montagnulan C (**3**).

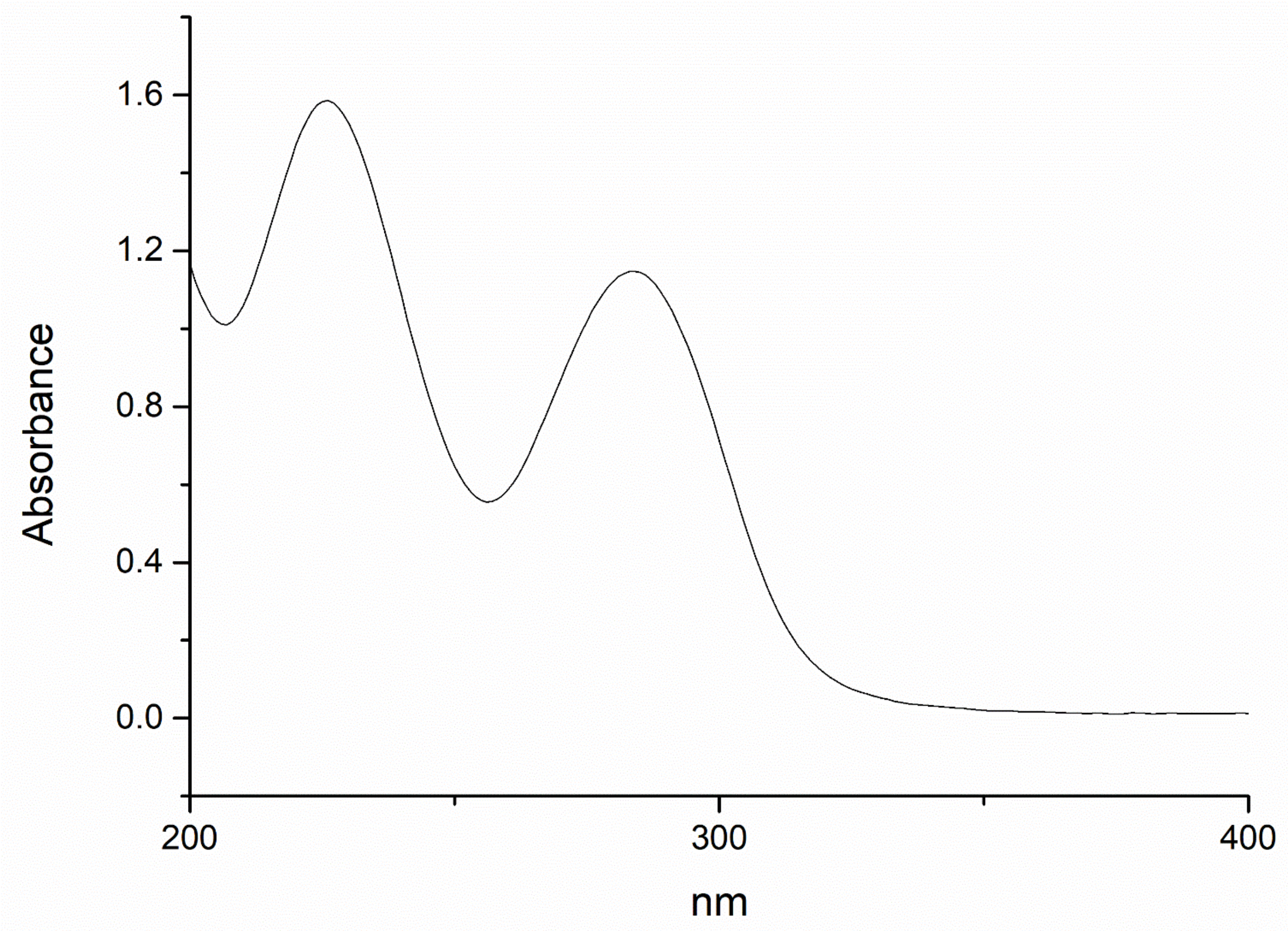

Figure S25. UV spectrum of montagnulan C (**3**).

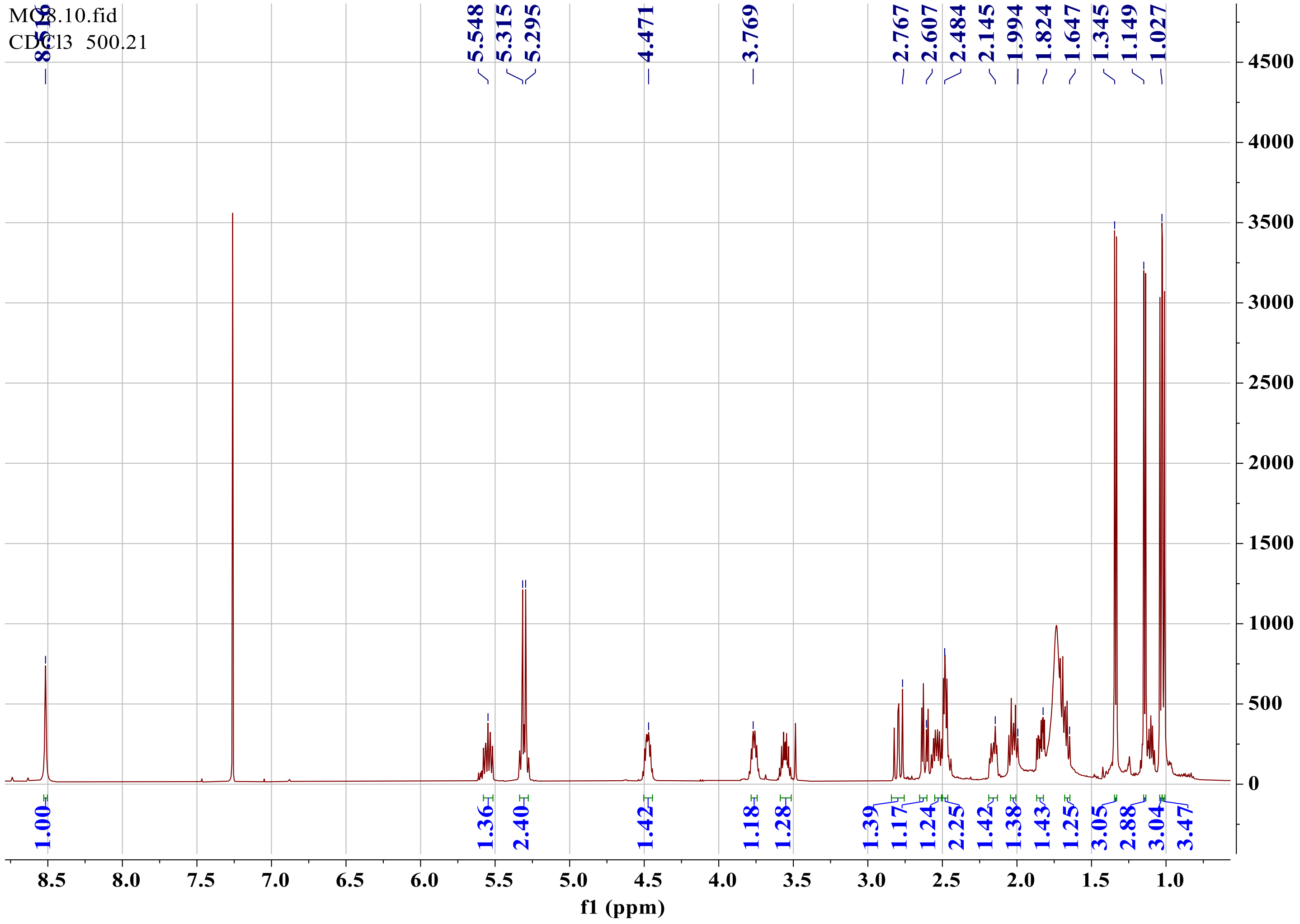

Figure S26. ^{1}H NMR spectrum of montagnulan D (**4**). ($CDCl_3$, 500 MHz).

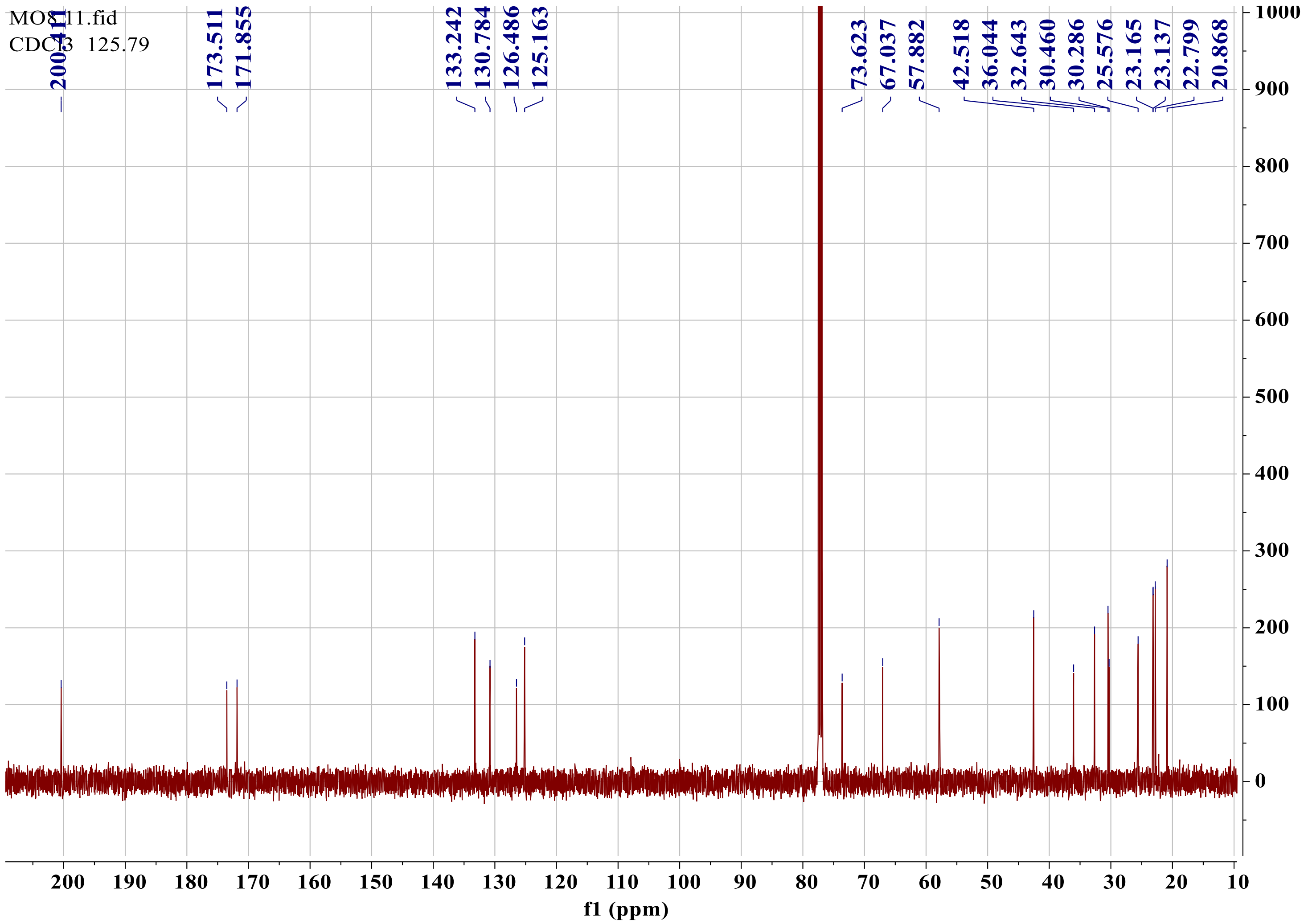

Figure S27. ^{13}C NMR spectrum of montagnulan D (**4**) ($CDCl_3$, 125 MHz).

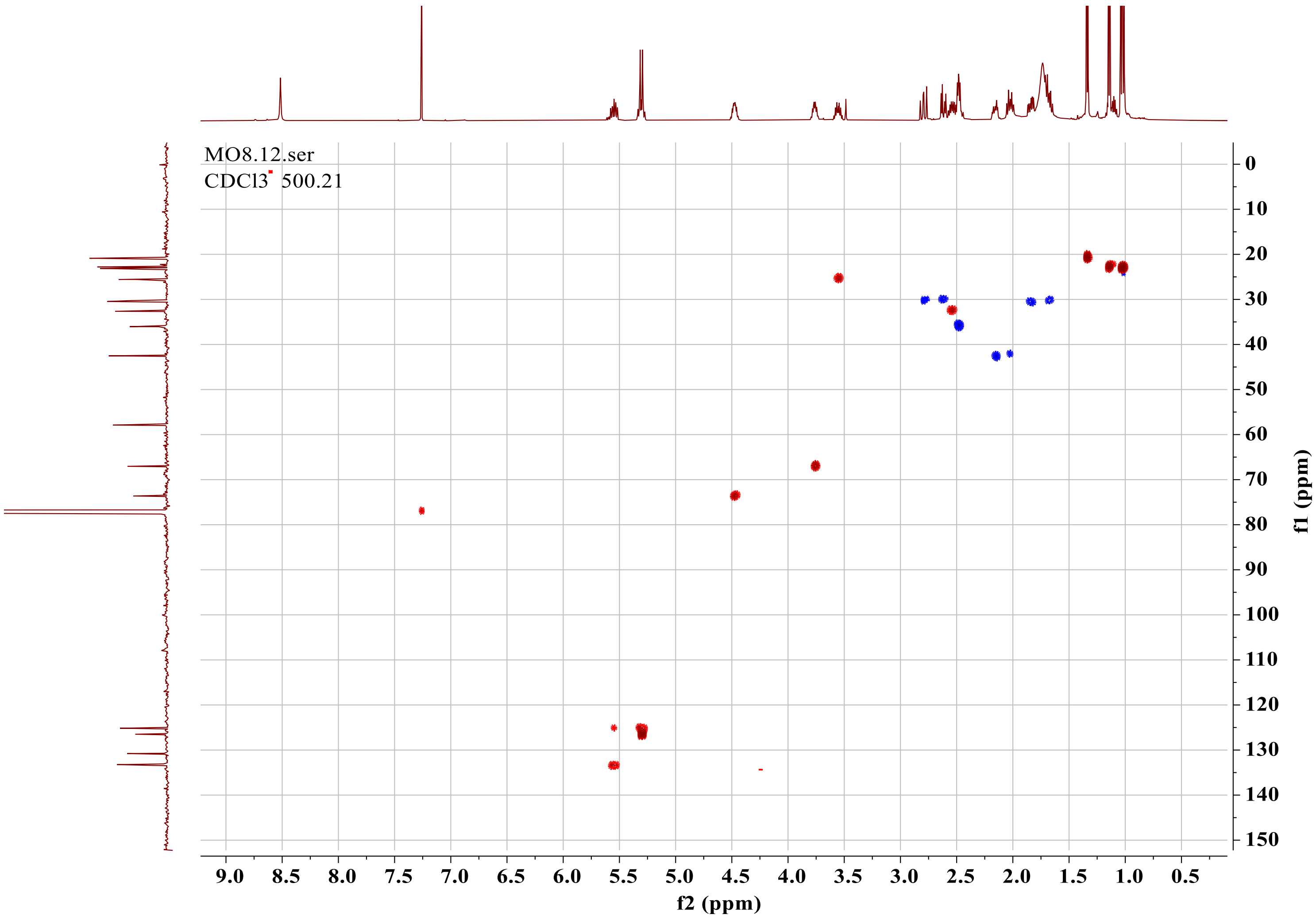

Figure S28. HSQC spectrum of montagnulan D (**4**) ($CDCl_3$).

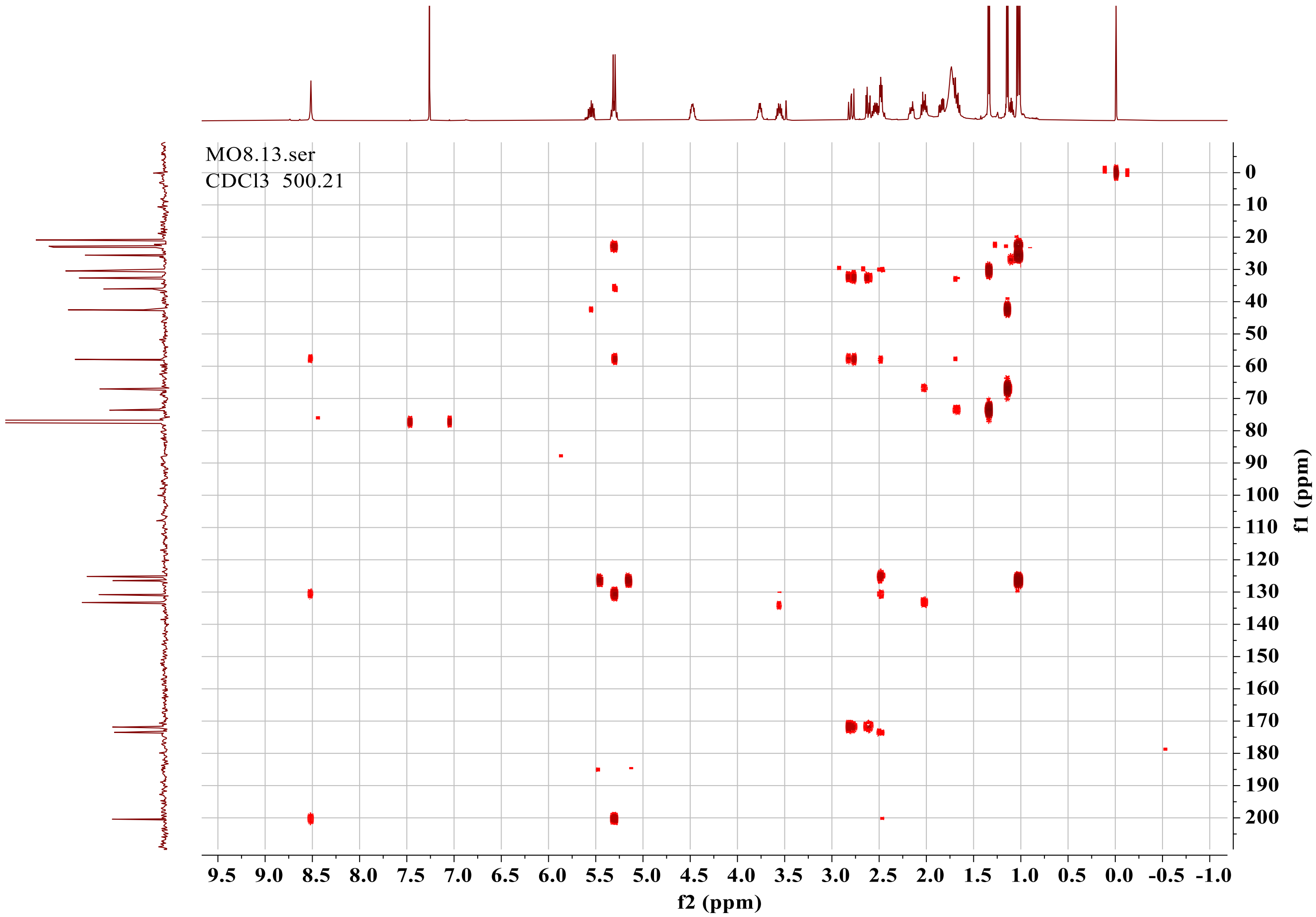

Figure S29. HMBC spectrum of montagnulan D (**4**) ($CDCl_3$).

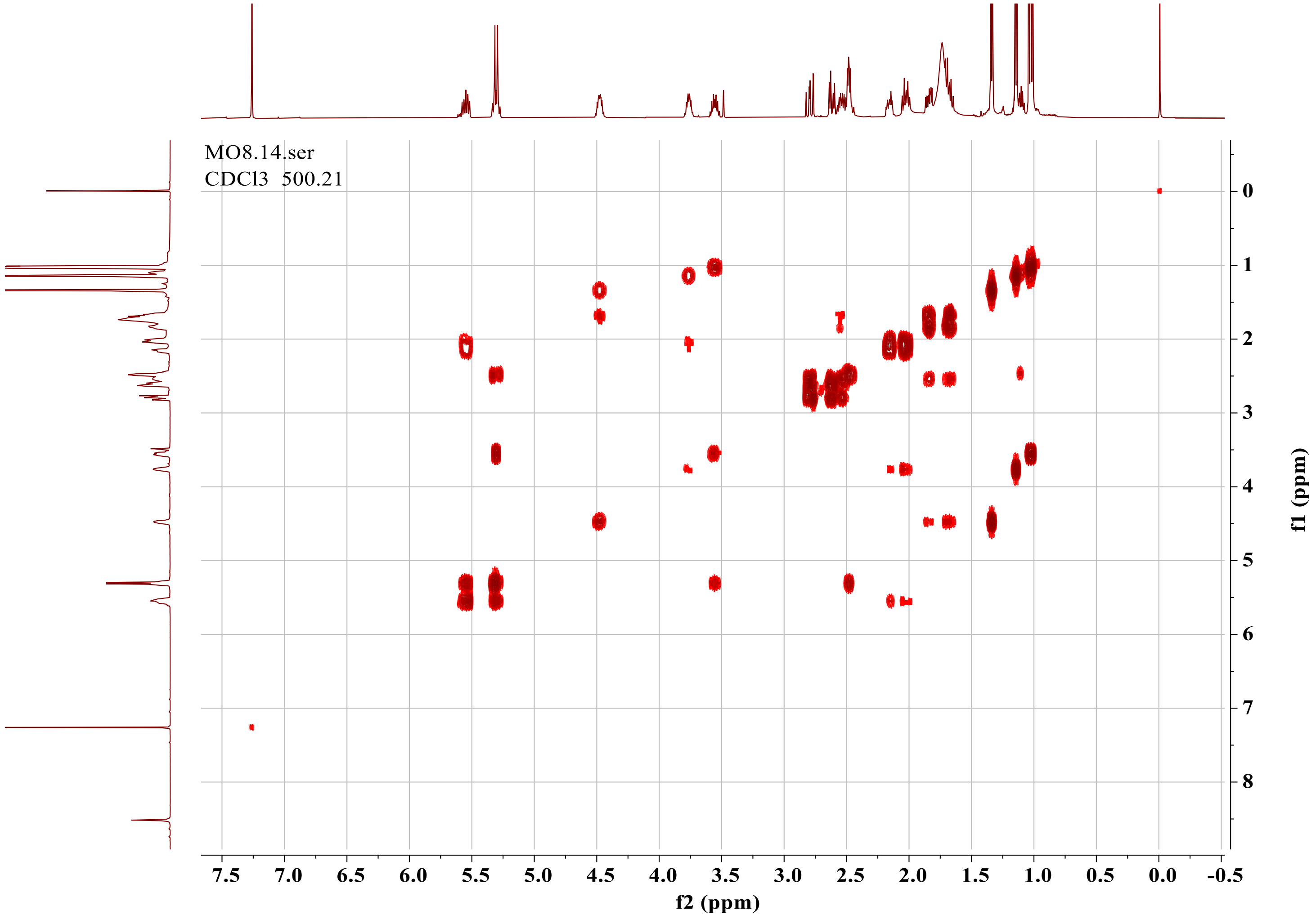

Figure S30. ^{1}H-^{1}H COSY spectrum of montagnulan D (**4**) ($CDCl_3$).

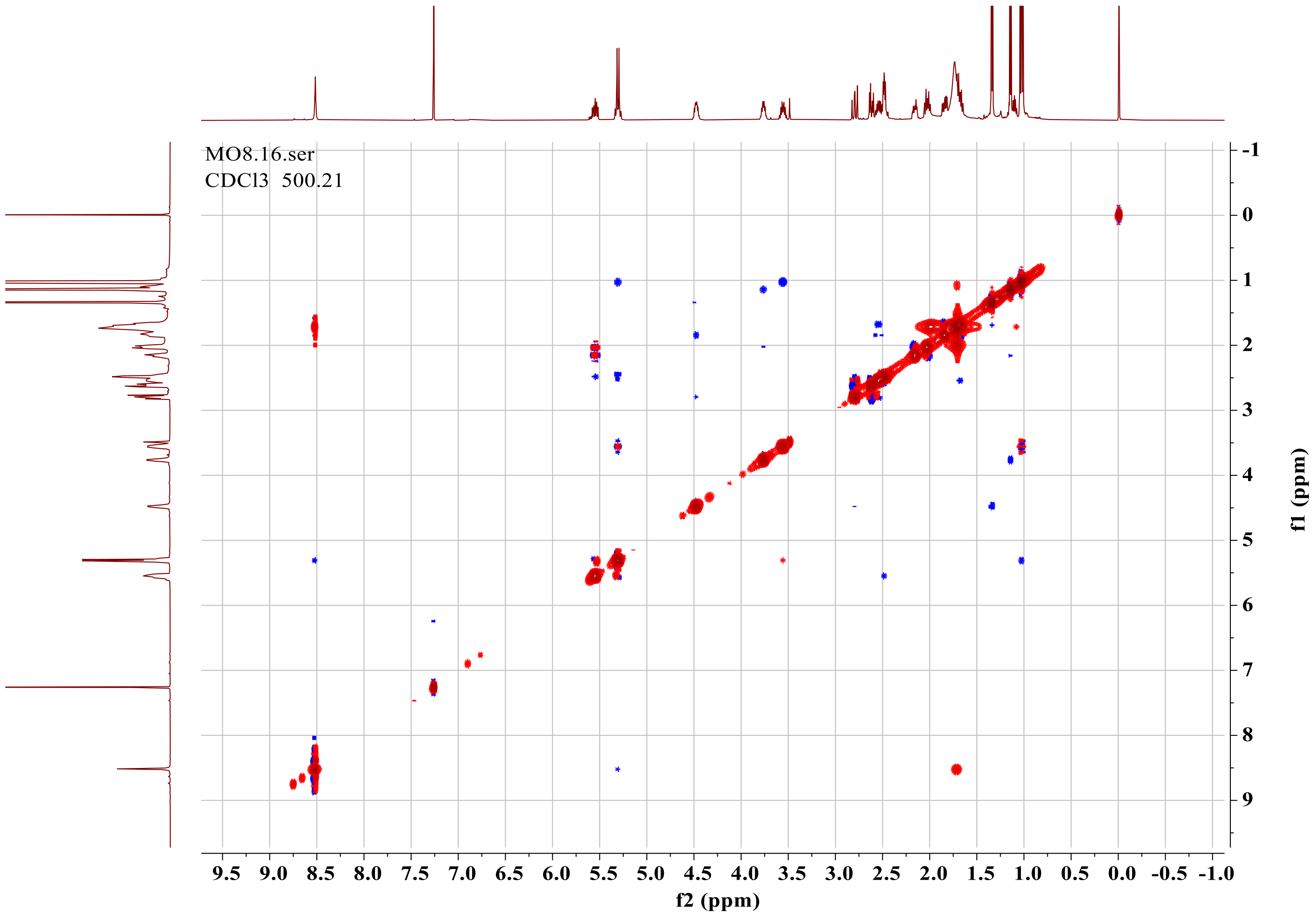

Figure S31. NOESY spectrum of montagnulan D (**4**) ($CDCl_3$).

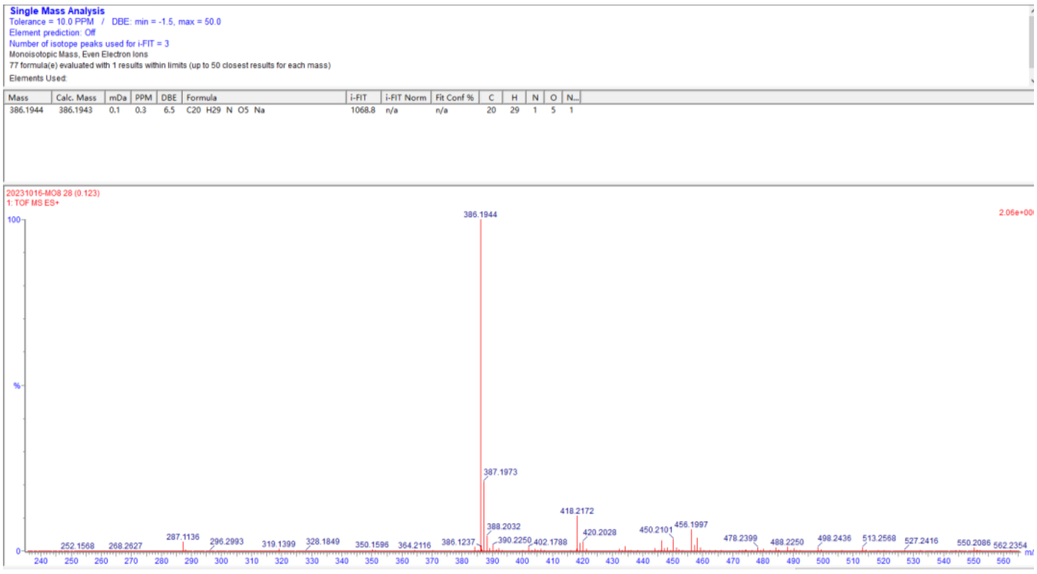

Figure S32. HR-ESIMS spectrum of montagnulan D (**4**).

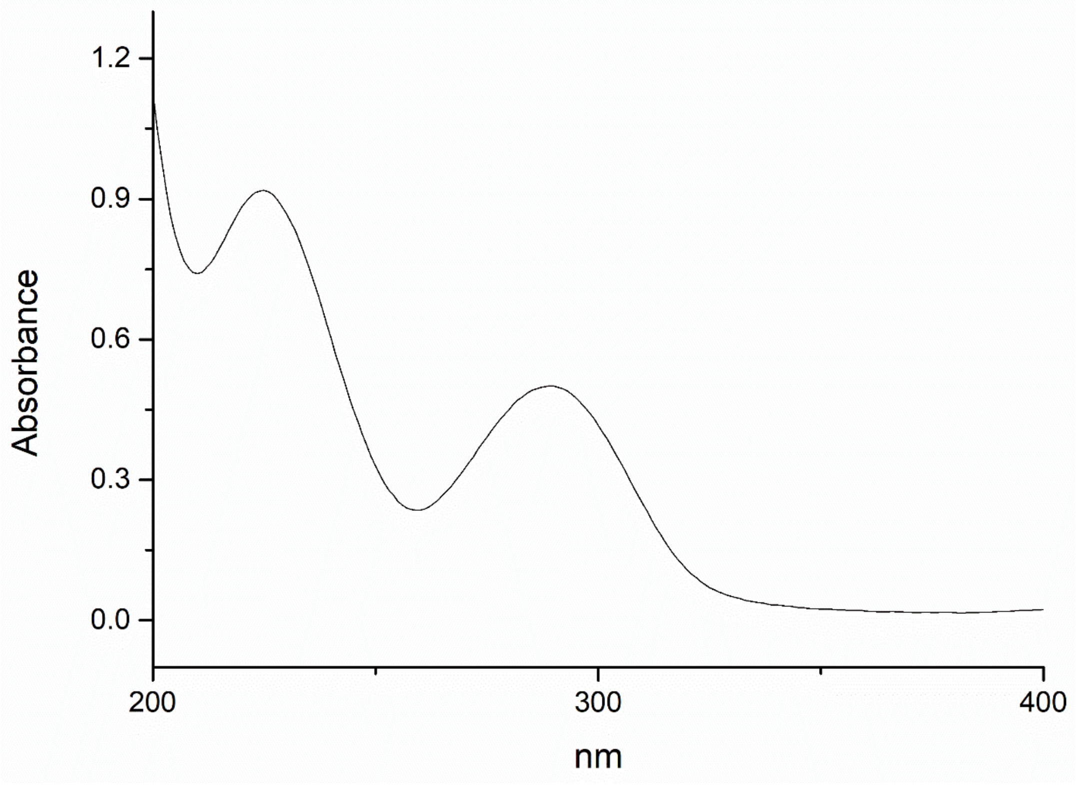

Figure S33. UV spectrum of montagnulan D (**4**).

Table S1. Energies of **1** at MMFF94 force field.

Configuration	Conformer	Energy (kcal/mol)	Population (%)
(3*S*, 10*R*, 12*R*)-**1**	1	66.53	27.2
(3*S*, 10*R*, 12*R*)-**1**	2	66.77	24.7
(3*S*, 10*R*, 12*R*)-**1**	3	66.98	22.7
(3*S*, 10*R*, 12*R*)-**1**	4	67.33	19.7
(3*S*, 10*R*, 12*R*)-**1**	5	73.31	1.8
(3*S*, 10*R*, 12*R*)-**1**	6	76.57	0.5
(3*R*, 10*S*, 12*S*)-**1**	1	66.53	27.2
(3*R*, 10*S*, 12*S*)-**1**	2	66.77	24.7
(3*R*, 10*S*, 12*S*)-**1**	3	66.98	22.7
(3*R*, 10*S*, 12*S*)-**1**	4	67.33	19.7
(3*R*, 10*S*, 12*S*)-**1**	5	73.31	1.8
(3*R*, 10*S*, 12*S*)-**1**	6	76.57	0.5
(3*R*, 10*R*, 12*R*)-**1**	1	66.47	28.4
(3*R*, 10*R*, 12*R*)-**1**	2	66.98	23.1
(3*R*, 10*R*, 12*R*)-**1**	3	67.15	21.6
(3*R*, 10*R*, 12*R*)-**1**	4	67.23	20.9
(3*R*, 10*R*, 12*R*)-**1**	5	73.08	2.0
(3*R*, 10*R*, 12*R*)-**1**	6	76.50	0.5
(3*S*, 10*S*, 12*S*)-**1**	1	66.47	28.5
(3*S*, 10*S*, 12*S*)-**1**	2	66.98	23.1
(3*S*, 10*S*, 12*S*)-**1**	3	67.15	21.6
(3*S*, 10*S*, 12*S*)-**1**	4	67.23	20.9
(3*S*, 10*S*, 12*S*)-**1**	5	73.08	2.0
(3*S*, 10*S*, 12*S*)-**1**	6	76.50	0.5

Table S2. Energies of **1** at B3LYP/6–31+g(d) level in methanol.

Configuration	Conformer	E (Hartree)	E (kcal/mol)	Population (%)

(3*S*, 10*R*, 12*R*)-**1**	1	–979.5804376	–614696.520398376	51.25
(3*S*, 10*R*, 12*R*)-**1**	2	–979.578559	–614695.34155809	6.99
(3*S*, 10*R*, 12*R*)-**1**	3	–979.5794648	–614695.909956648	18.27
(3*S*, 10*R*, 12*R*)-**1**	4	–979.5795245	–614695.947418995	19.47
(3*S*, 10*R*, 12*R*)-**1**	5	–979.5775189	–614694.688884939	2.32
(3*S*, 10*R*, 12*R*)-**1**	6	–979.5772228	–614694.503079228	1.70
(3*R*, 10*S*, 12*S*)-**1**	1	–979.5804376	–614696.520398376	51.25
(3*R*, 10*S*, 12*S*)-**1**	2	–979.578559	–614695.34155809	6.99
(3*R*, 10*S*, 12*S*)-**1**	3	–979.5794643	–614695.909642893	18.26
(3*R*, 10*S*, 12*S*)-**1**	4	–979.579525	–614695.94773275	19.48
(3*R*, 10*S*, 12*S*)-**1**	5	–979.5775188	–614694.688822188	2.32
(3*R*, 10*S*, 12*S*)-**1**	6	–979.5772228	–614694.503079228	1.70
(3*R*, 10*R*, 12*R*)-**1**	1	–979.5804224	–614696.510860224	52.85
(3*R*, 10*R*, 12*R*)-**1**	2	–979.5788242	–614695.507973742	9.71
(3*R*, 10*R*, 12*R*)-**1**	3	–979.5794076	–614695.874063076	18.02
(3*R*, 10*R*, 12*R*)-**1**	4	–979.5792206	–614695.756718706	14.78
(3*R*, 10*R*, 12*R*)-**1**	5	–979.5776577	–614694.775983327	2.82
(3*R*, 10*R*, 12*R*)-**1**	6	–979.5772397	–614694.513684147	1.81
(3*S*, 10*S*, 12*S*)-**1**	1	–979.5804224	–614696.510860224	52.85
(3*S*, 10*S*, 12*S*)-**1**	2	–979.5788243	–614695.508036493	9.71
(3*S*, 10*S*, 12*S*)-**1**	3	–979.5794069	–614695.873623819	18.02
(3*S*, 10*S*, 12*S*)-**1**	4	–979.5792206	–614695.756718706	14.78
(3*S*, 10*S*, 12*S*)-**1**	5	–979.5776576	–614694.775920576	2.82
(3*S*, 10*S*, 12*S*)-**1**	6	–979.5772397	–614694.513684147	1.81

(3*S*, 10*R*, 12*R*)-**1**

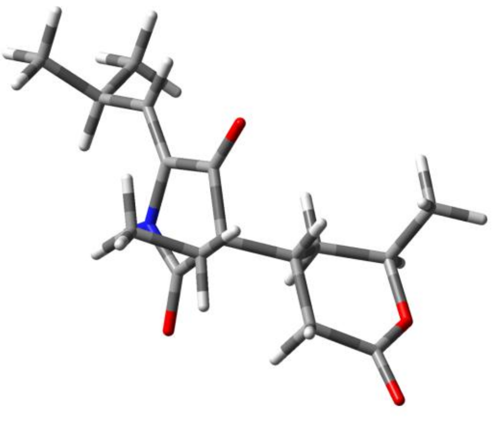	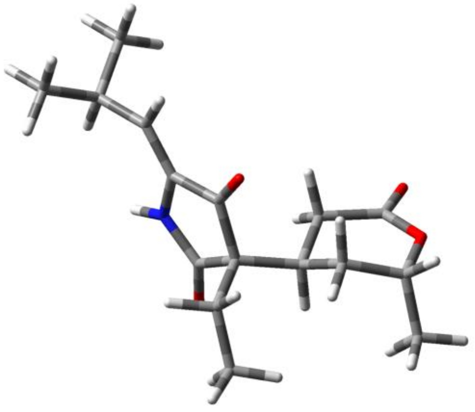
Conf.1 (51.25%)	Conf.2 (6.99%)
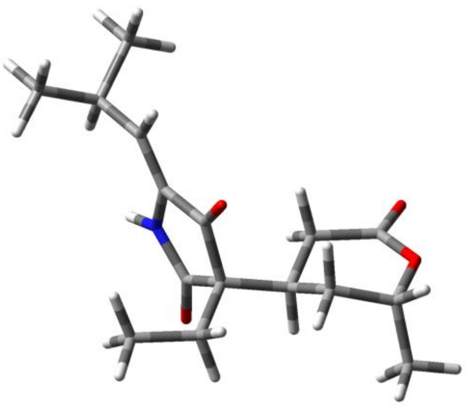	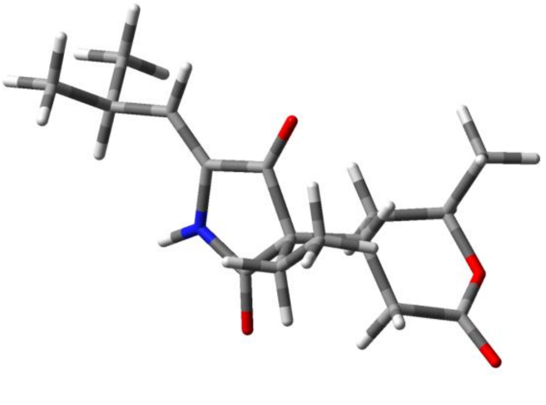
Conf.3 (18.27%)	Conf.4 (19.47%)
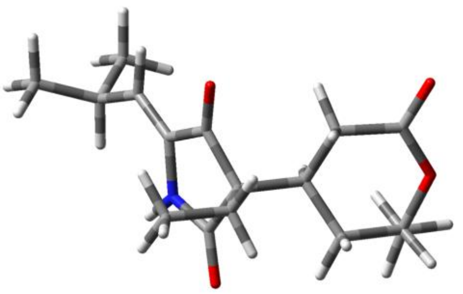	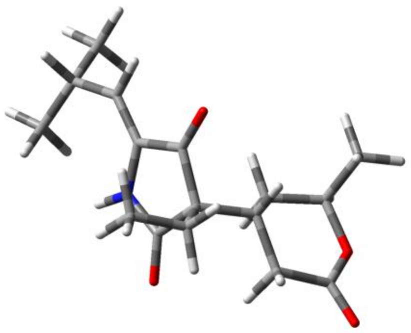
Conf.5 (2.32%)	Conf.6 (1.70%)

(3*R*, 10*S*, 12*S*)-**1**

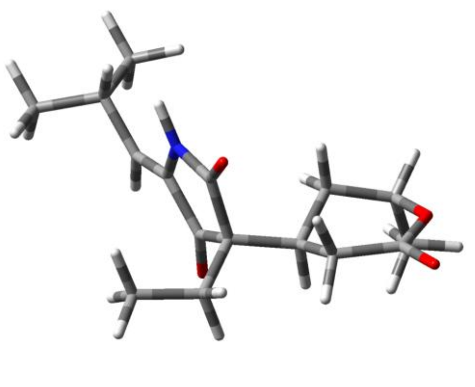	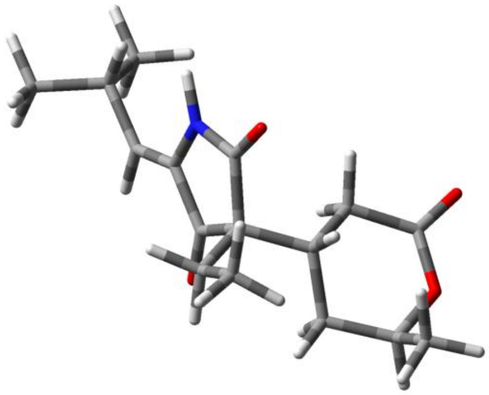
Conf.1 (51.25%)	Conf.2 (6.99%)
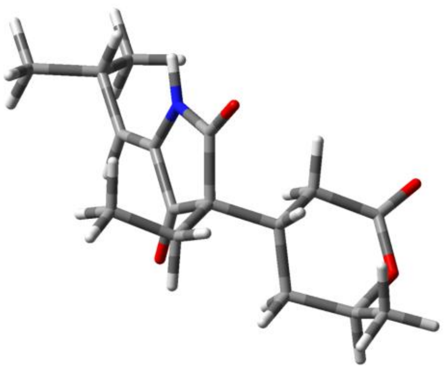	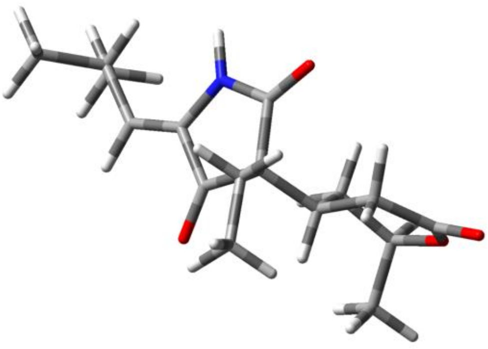
Conf.3 (18.27%)	Conf.4 (19.47%)
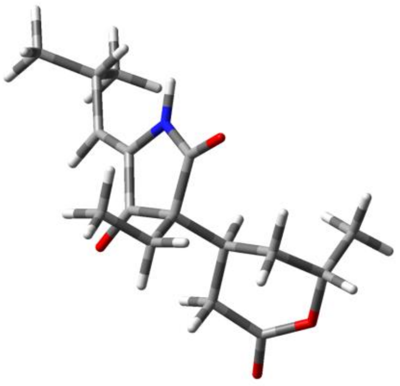	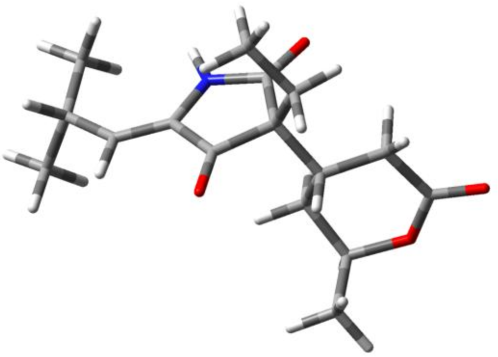
Conf.5 (2.32%)	Conf.6 (1.70%)

(3*R*, 10*R*, 12*R*)-**1**

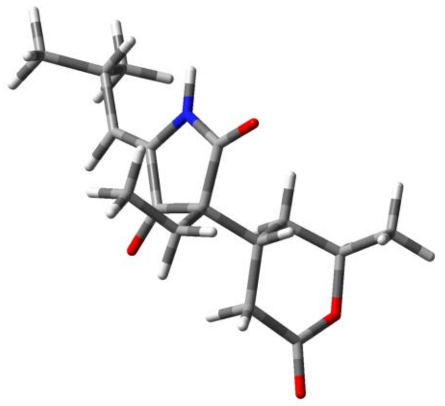	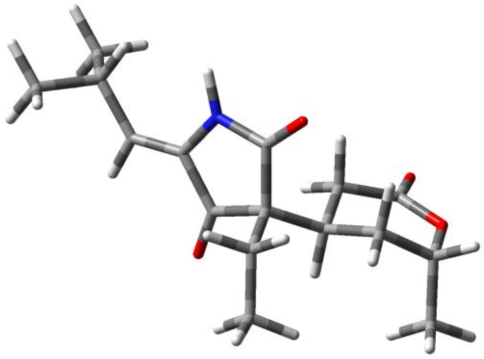
Conf.1 (52.85%)	Conf.2 (9.71%)
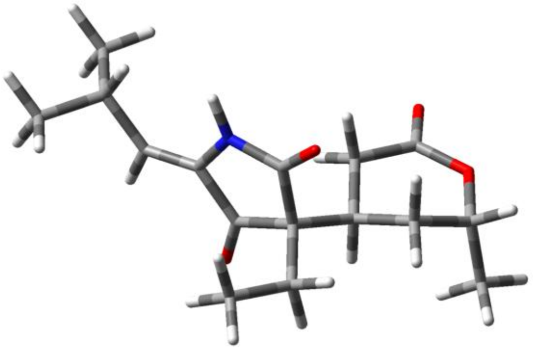	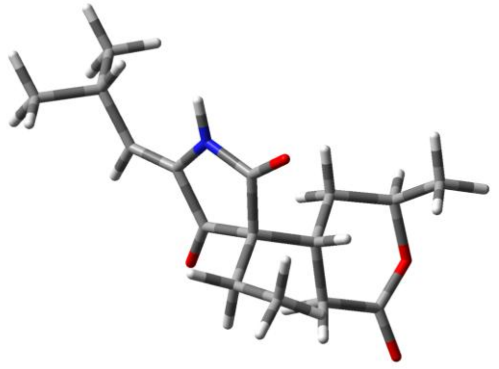
Conf.3 (18.02%)	Conf.4 (14.78%)
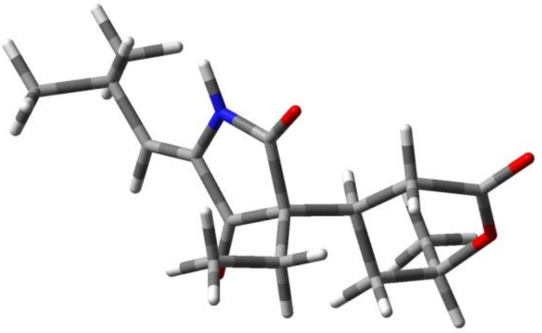	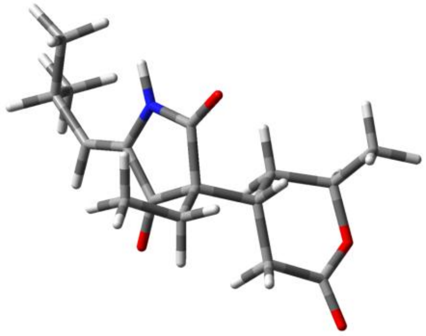
Conf.5 (2.82%)	Conf.6 (1.81%)

(3*S*, 10*S*, 12*S*)-**1**

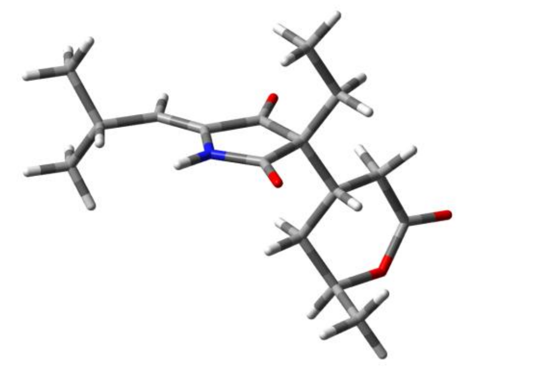	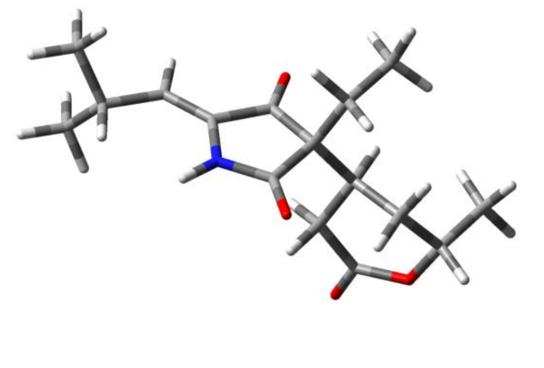
Conf.1 (52.85%)	Conf.2 (9.71%)
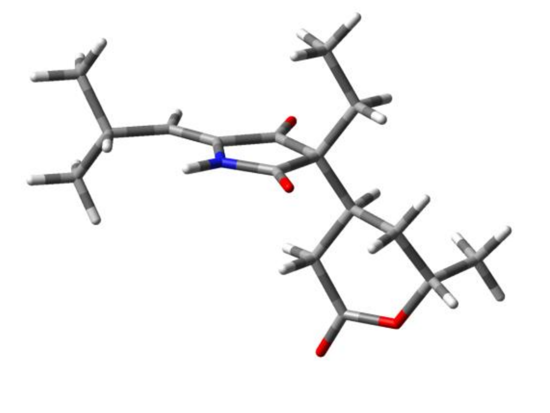	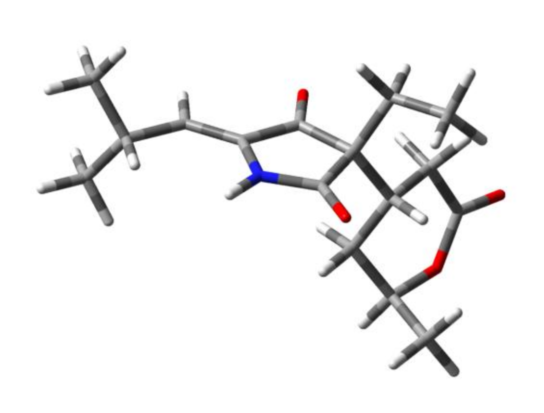
Conf.3 (18.02%)	Conf.4 (14.78%)
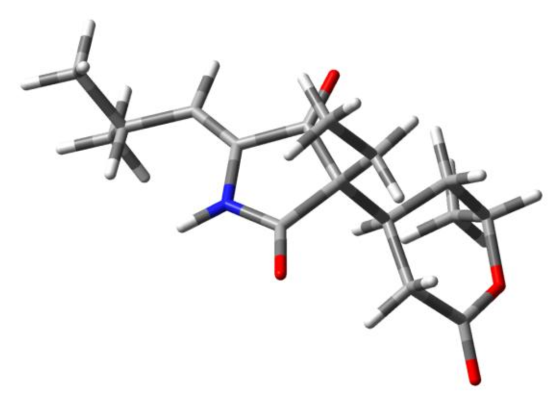	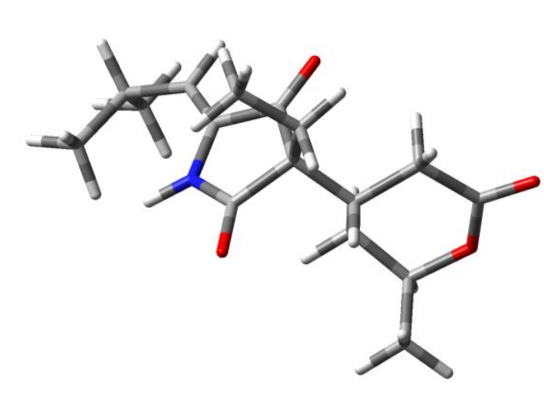
Conf.5 (2.82%)	Conf.6 (1.81%)

Figure S34. The optimized conformers and equilibrium populations of **1**.

Table S3. Energies of **2** at MMFF94 force field.

Configuration	Conformer	Energy (kcal/mol)	Population (%)
(3*S*, 10*S*, 12*S*)-**2**	1	66.47	28.5
(3*S*, 10*S*, 12*S*)-**2**	2	66.98	23.1
(3*S*, 10*S*, 12*S*)-**2**	3	67.15	21.6

(3*S*, 10*S*, 12*S*)-**2**	4	67.23	20.9
(3*S*, 10*S*, 12*S*)-**2**	5	73.08	2.0
(3*S*, 10*S*, 12*S*)-**2**	6	76.50	0.5

Table S4. Energies of **2** at B3LYP/6–31+g(d) level in methanol.

Configuration	Conformer	E (Hartree)	E (kcal/mol)	Population (%)
(3*S*, 10*S*, 12*S*)-**2**	1	–979.5804224	–614696.510860224	52.85
(3*S*, 10*S*, 12*S*)-**2**	2	–979.5788243	–614695.508036493	9.71
(3*S*, 10*S*, 12*S*)-**2**	3	–979.5794069	–614695.873623819	18.02
(3*S*, 10*S*, 12*S*)-**2**	4	–979.5792206	–614695.756718706	14.78
(3*S*, 10*S*, 12*S*)-**2**	5	–979.5776576	–614694.775920576	2.82
(3*S*, 10*S*, 12*S*)-**2**	6	–979.5772397	–614694.513684147	1.81

(3*S*, 10*S*, 12*S*)-**2**

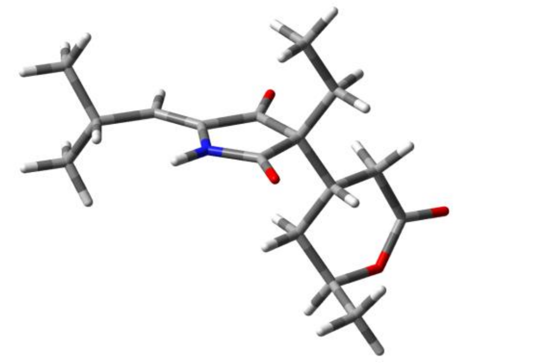	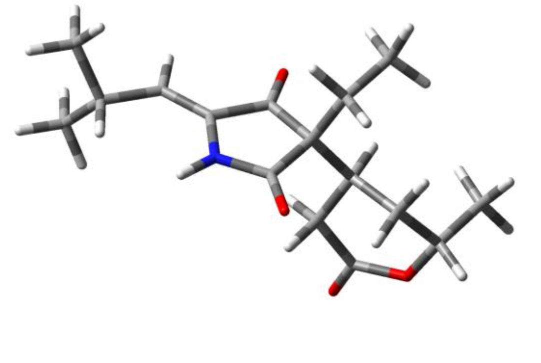
Conf.1 (52.85%)	Conf.2 (9.71%)
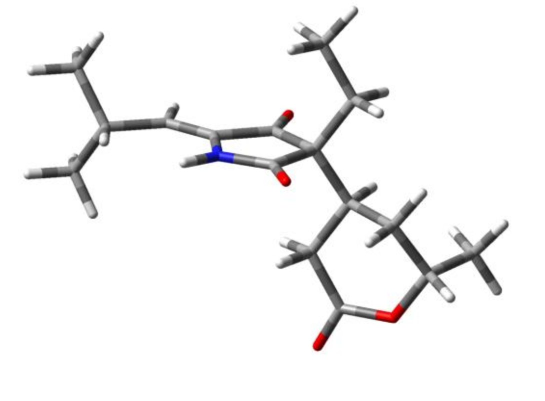	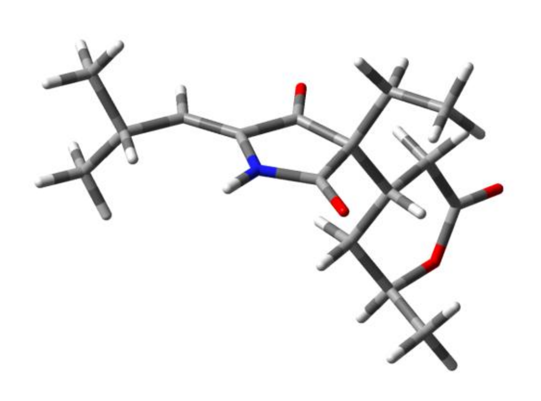
Conf.3 (18.02%)	Conf.4 (14.78%)

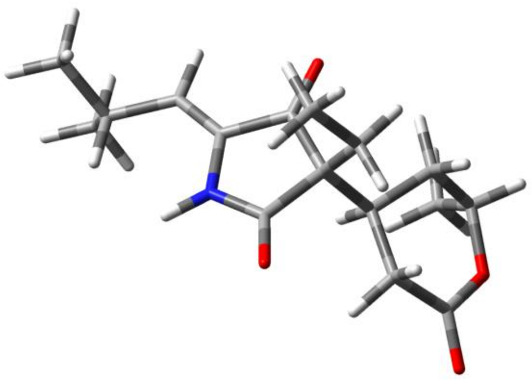	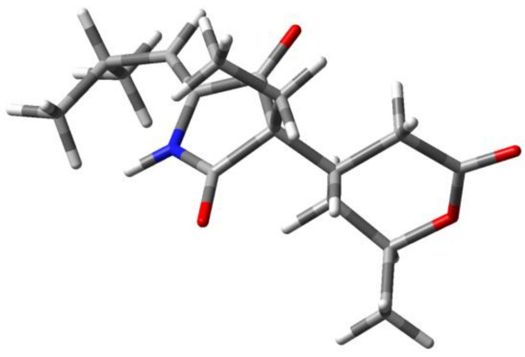
Conf.5 (2.82%)	Conf.6 (1.81%)

Figure S35. The optimized conformers and equilibrium populations of **2**.

Table S5. Energies of **3** at MMFF94 force field.

Configuration	Conformer	Energy (kcal/mol)	Population (%)
(3*S*, 10*R*, 12*S*)-**3**	1	62.95	24.7
(3*S*, 10*R*, 12*S*)-**3**	2	63.59	19.1
(3*S*, 10*R*, 12*S*)-**3**	3	63.65	18.6
(3*S*, 10*R*, 12*S*)-**3**	4	63.91	16.8
(3*S*, 10*R*, 12*S*)-**3**	5	65.16	10.2
(3*S*, 10*R*, 12*S*)-**3**	6	66.87	5.1
(3*S*, 10*R*, 12*S*)-**3**	7	69.79	1.6
(3*S*, 10*R*, 12*S*)-**3**	8	72.98	0.4
(3*R*, 10*R*, 12*S*)-**3**	1	62.85	25.7
(3*R*, 10*R*, 12*S*)-**3**	2	63.75	17.8
(3*R*, 10*R*, 12*S*)-**3**	3	63.79	17.6
(3*R*, 10*R*, 12*S*)-**3**	4	63.85	17.2
(3*R*, 10*R*, 12*S*)-**3**	5	65.20	9.9
(3*R*, 10*R*, 12*S*)-**3**	6	66.90	5.0
(3*R*, 10*R*, 12*S*)-**3**	7	69.60	1.7
(3*R*, 10*R*, 12*S*)-**3**	8	70.39	1.2
(3*R*, 10*R*, 12*S*)-**3**	9	72.88	0.4
(3*S*, 10*S*, 12*R*)-**3**	1	62.85	58.7

(3*S*, 10*S*, 12*R*)-**3**	2	65.20	22.7
(3*S*, 10*S*, 12*R*)-**3**	3	66.90	11.5
(3*S*, 10*S*, 12*R*)-**3**	4	70.39	2.8
(3*S*, 10*S*, 12*R*)-**3**	5	72.88	1.0
(3*S*, 10*S*, 12*R*)-**3**	6	72.92	1.0
(3*S*, 10*S*, 12*R*)-**3**	7	73.26	0.9
(3*S*, 10*S*, 12*R*)-**3**	8	75.27	0.4
(3*R*, 10*S*, 12*R*)-**3**	1	62.95	24.4
(3*R*, 10*S*, 12*R*)-**3**	2	63.59	18.9
(3*R*, 10*S*, 12*R*)-**3**	3	63.65	18.4
(3*R*, 10*S*, 12*R*)-**3**	4	63.91	16.6
(3*R*, 10*S*, 12*R*)-**3**	5	65.16	10.0
(3*R*, 10*S*, 12*R*)-**3**	6	66.87	5.0
(3*R*, 10*S*, 12*R*)-**3**	7	69.79	1.6
(3*R*, 10*S*, 12*R*)-**3**	8	70.52	1.2
(3*R*, 10*S*, 12*R*)-**3**	9	72.98	0.4

Table S6. Energies of **3** at B3LYP/6–31+g(d) level in methanol.

Configuration	Conformer	E (Hartree)	E (kcal/mol)	Population (%)
(3*S*, 10*R*, 12*S*)-**3**	1	–979.5822314	–614697.646025814	47.58
(3*S*, 10*R*, 12*S*)-**3**	2	–979.5812674	–614697.041106174	17.12
(3*S*, 10*R*, 12*S*)-**3**	3	–979.5802292	–614696.389625292	5.70
(3*S*, 10*R*, 12*S*)-**3**	4	–979.5812284	–614697.016633284	16.43
(3*S*, 10*R*, 12*S*)-**3**	5	–979.5805469	–614696.588985219	7.98
(3*S*, 10*R*, 12*S*)-**3**	6	–979.5790386	–614695.642511886	1.61
(3*S*, 10*R*, 12*S*)-**3**	7	–979.5792555	–614695.778618805	2.03
(3*S*, 10*R*, 12*S*)-**3**	8	–979.5790067	–614695.622494317	1.56
(3*R*, 10*R*, 12*S*)-**3**	1	–979.5822252	–614697.642135252	48.26
(3*R*, 10*R*, 12*S*)-**3**	2	–979.5812503	–614697.030375753	17.17

(3*R*, 10*R*, 12*S*)-**3**	3	–979.5809696	–614696.854233696	12.75
(3*R*, 10*R*, 12*S*)-**3**	4	–979.5805472	–614696.589173472	8.15
(3*R*, 10*R*, 12*S*)-**3**	5	–979.5803613	–614696.472519363	6.69
(3*R*, 10*R*, 12*S*)-**3**	6	–979.57918	–614695.7312418	1.91
(3*R*, 10*R*, 12*S*)-**3**	7	–979.5794275	–614695.886550525	2.49
(3*R*, 10*R*, 12*S*)-**3**	8	–979.5785036	–614695.306794036	0.93
(3*R*, 10*R*, 12*S*)-**3**	9	–979.5790435	–614695.645586685	1.65
(3*S*, 10*S*, 12*R*)-**3**	1	–979.5822252	–614697.642135252	77.99
(3*S*, 10*S*, 12*R*)-**3**	2	–979.5803613	–614696.472519363	10.81
(3*S*, 10*S*, 12*R*)-**3**	3	–979.5791798	–614695.731116298	3.09
(3*S*, 10*S*, 12*R*)-**3**	4	–979.5785037	–614695.306856787	1.51
(3*S*, 10*S*, 12*R*)-**3**	5	–979.5790434	–614695.645523934	2.67
(3*S*, 10*S*, 12*R*)-**3**	6	–979.5789748	–614695.602476748	2.49
(3*S*, 10*S*, 12*R*)-**3**	7	–979.5781675	–614695.095887925	1.06
(3*S*, 10*S*, 12*R*)-**3**	8	–979.5771958	–614694.486136458	0.38
(3*R*, 10*S*, 12*R*)-**3**	1	–979.5822315	–614697.646088565	47.13
(3*R*, 10*S*, 12*R*)-**3**	2	–979.5812676	–614697.041231676	16.96
(3*R*, 10*S*, 12*R*)-**3**	3	–979.5802292	–614696.389625292	5.64
(3*R*, 10*S*, 12*R*)-**3**	4	–979.5812284	–614697.016633284	16.27
(3*R*, 10*S*, 12*R*)-**3**	5	–979.5805469	–614696.588985219	7.90
(3*R*, 10*S*, 12*R*)-**3**	6	–979.5790384	–614695.642386384	1.60
(3*R*, 10*S*, 12*R*)-**3**	7	–979.5792555	–614695.778618805	2.01
(3*R*, 10*S*, 12*R*)-**3**	8	–979.5785423	–614695.331078673	0.94
(3*R*, 10*S*, 12*R*)-**3**	9	–979.5790065	–614695.622368815	1.54

(3*S*, 10*R*, 12*S*)-**3**

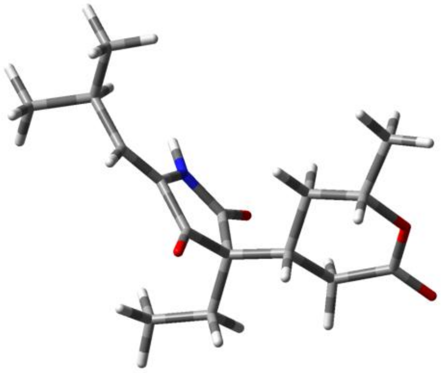	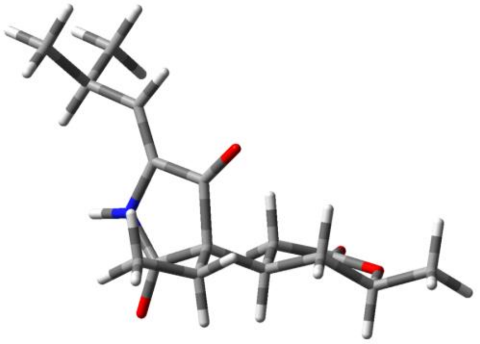
Conf.1 (47.58%)	Conf.2 (17.12%)
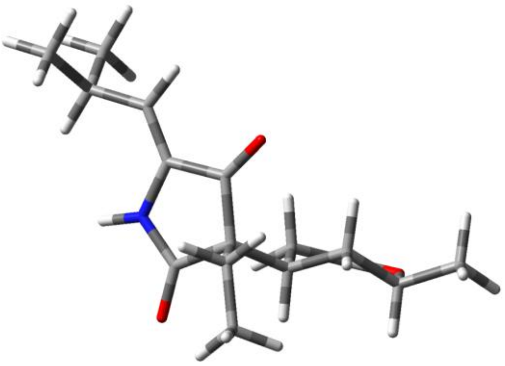	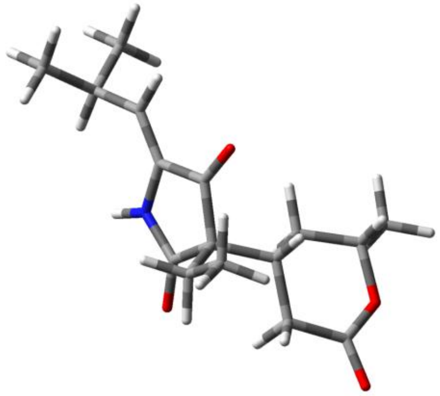
Conf.3 (5.70%)	Conf.4 (16.43%)
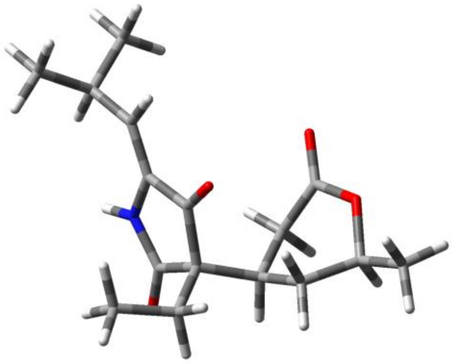	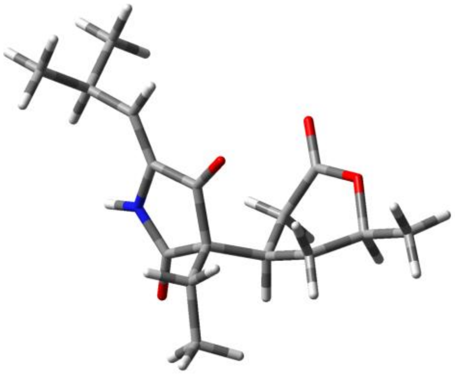
Conf.5 (7.98%)	Conf.6 (1.61%)
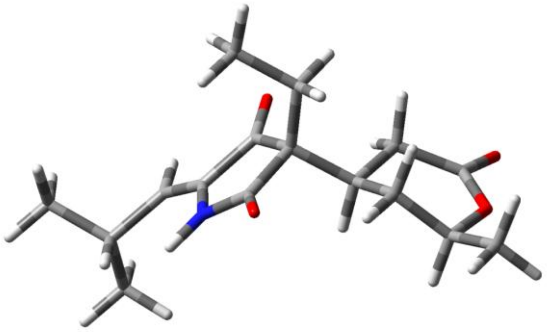	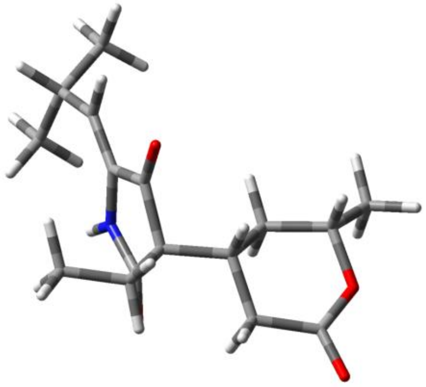
Conf.7 (2.03%)	Conf.8 (1.56%)

(3*R*, 10*R*, 12*S*)-**3**

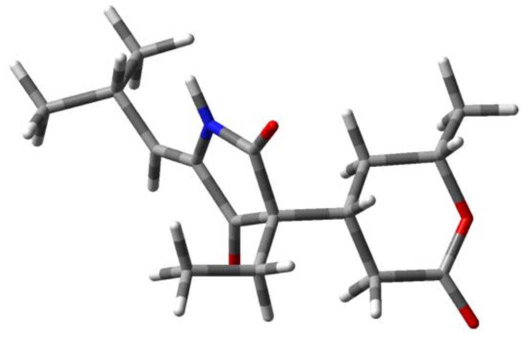	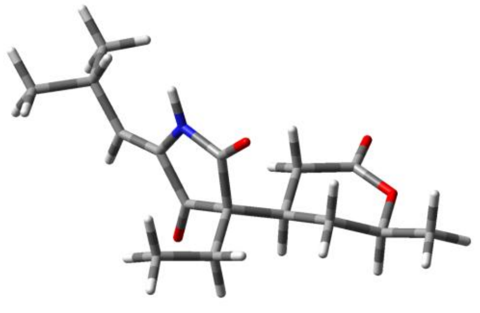
Conf.1 (48.26%)	Conf.2 (17.17%)
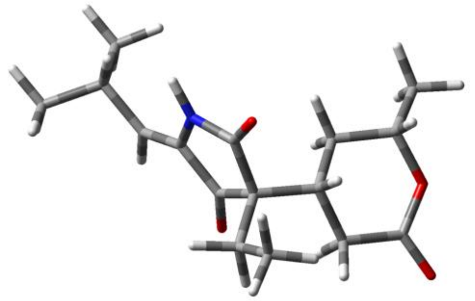	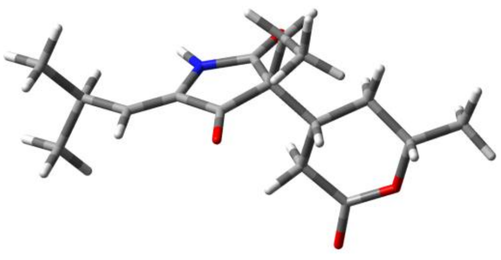
Conf.3 (12.75%)	Conf.4 (8.15%)
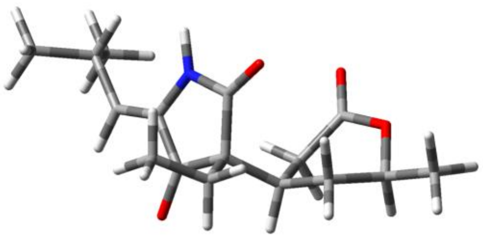	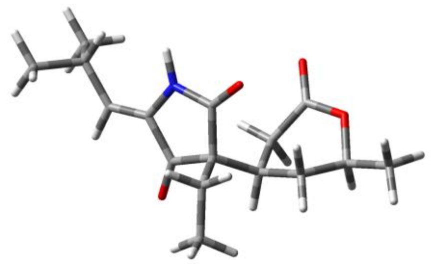
Conf.5 (6.69%)	Conf.6 (1.91%)
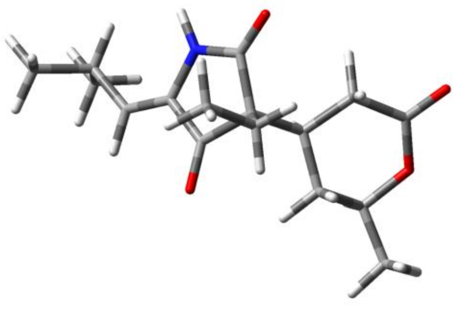	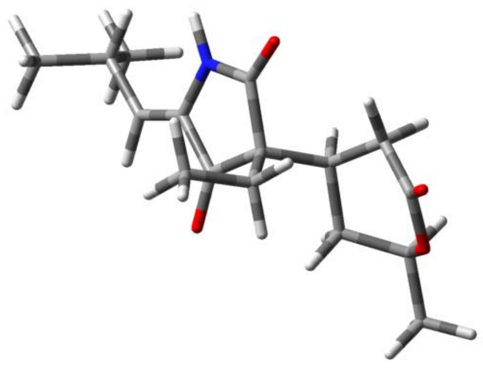
Conf.7 (2.49%)	Conf.8 (0.93%)

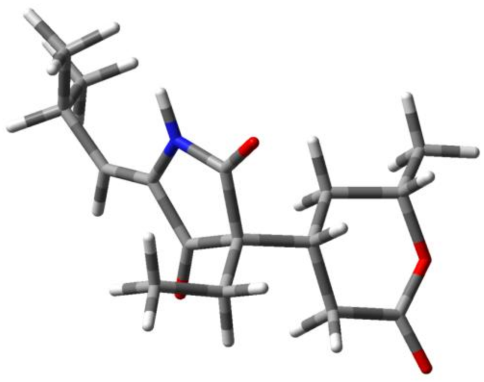	
Conf.9 (1.65%)	

(3*S*, 10*S*, 12*R*)-**3**

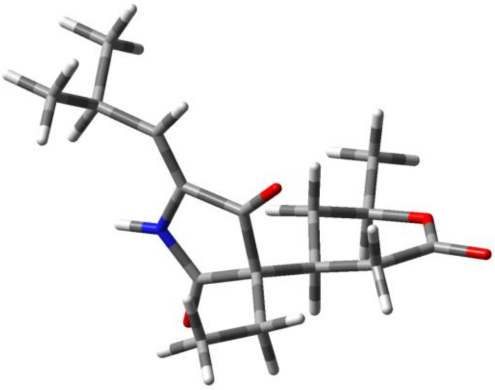	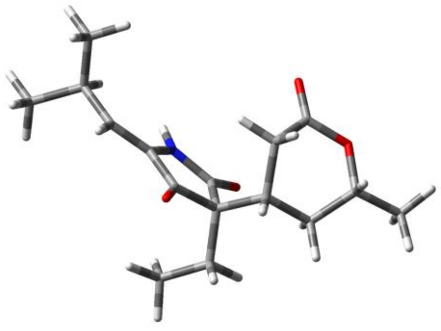
Conf.1 (77.99%)	Conf.2 (10.81%)
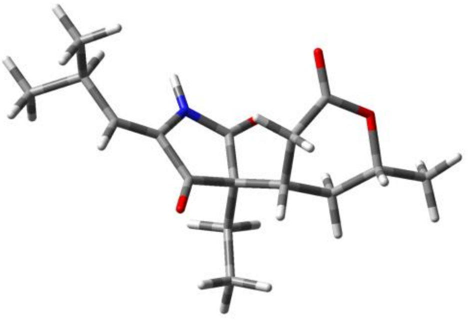	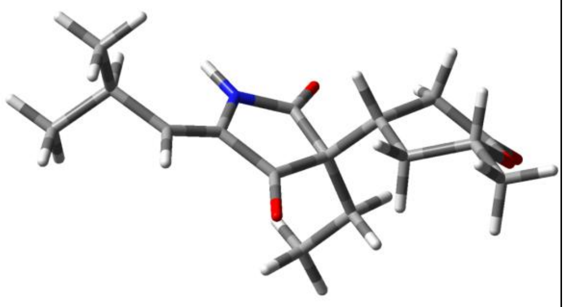
Conf.3 (3.09%)	Conf.4 (1.51%)

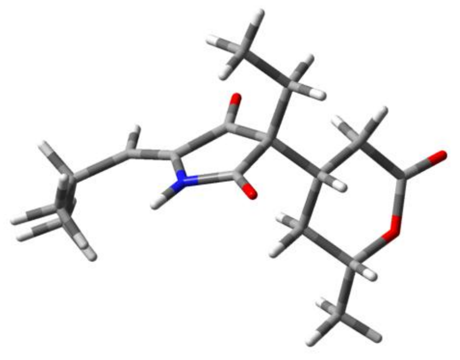	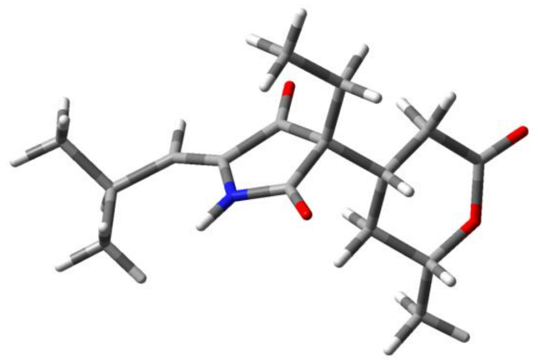
Conf.5 (2.67%)	Conf.6 (2.49%)
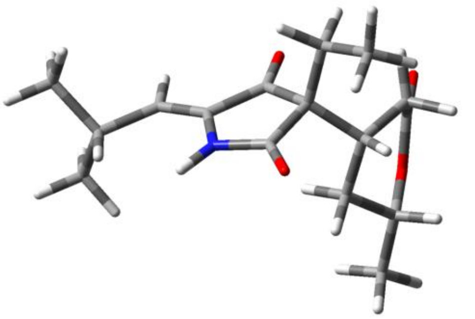	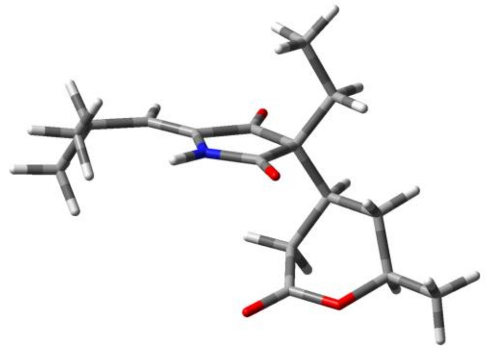
Conf.7 (1.06%)	Conf.8 (0.38%)

(3*R*, 10*S*, 12*R*)-**3**

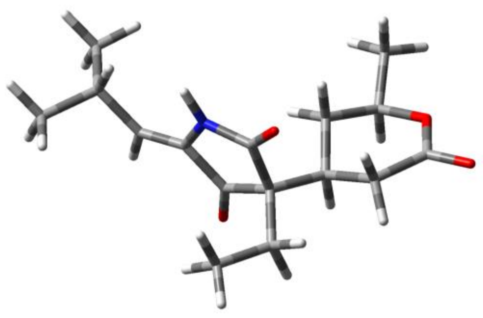	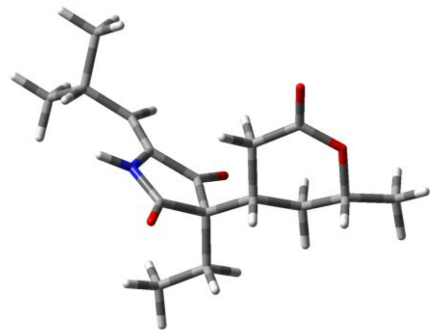
Conf.1 (47.13%)	Conf.2 (16.96%)
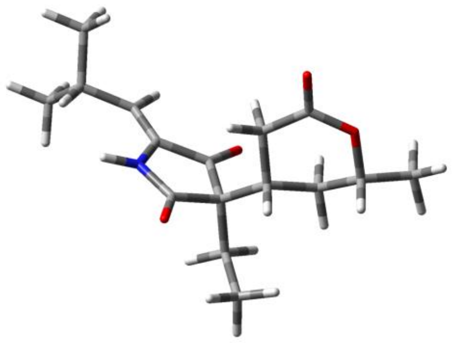	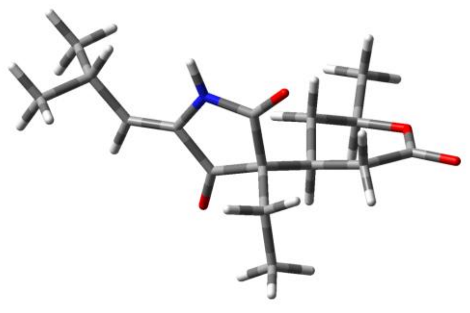

Conf.3 (5.64%)	Conf.4 (16.27%)
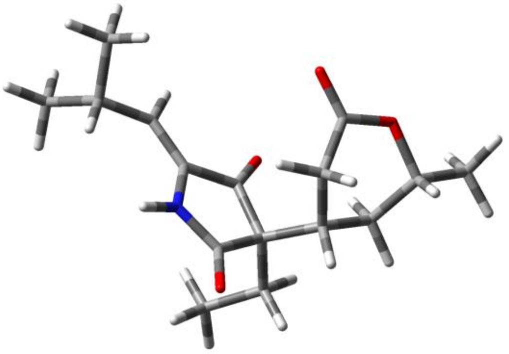	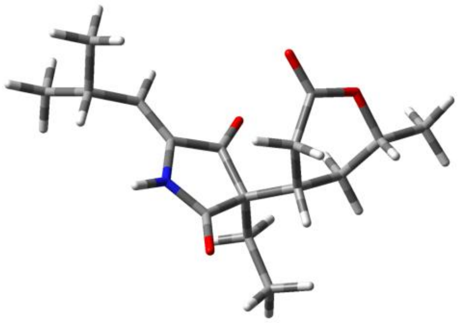
Conf.5 (7.90%)	Conf.6 (1.60%)
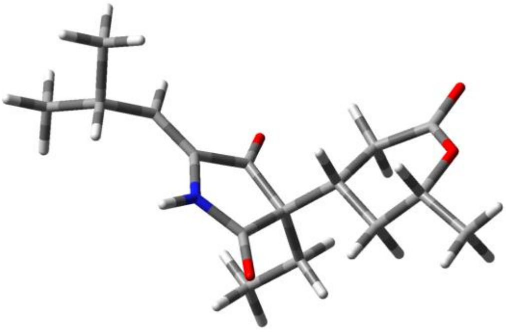	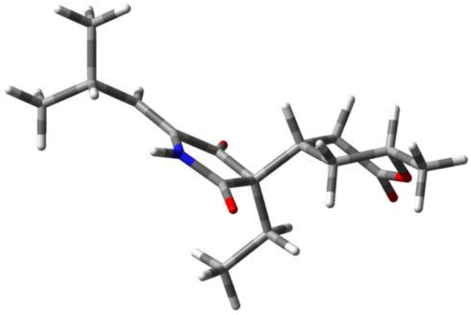
Conf.7 (2.01%)	Conf.8 (0.94%)
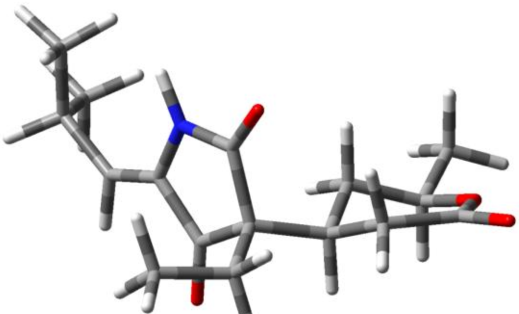	
Conf.9 (1.54%)	

Figure S36. The optimized conformers and equilibrium populations of **3**.

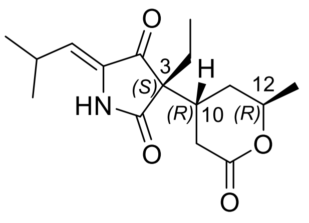

(3*S*, 10*R*, 12*R*)-**1**

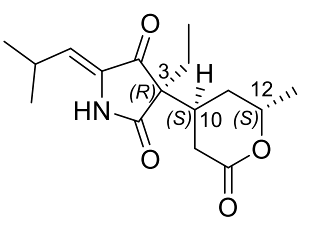

(3*R*, 10*S*, 12*S*)-**1**

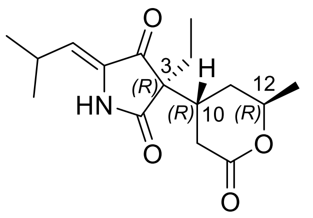

(3*R*, 10*R*, 12*R*)-**1**

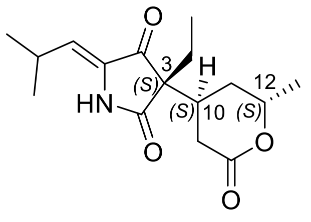

(3*S*, 10*S*, 12*S*)-**1**

Figure S37. Structures of four possible isomers of **1** for ECD calculations.

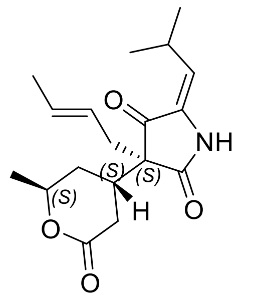

M-(3*S*, 10*S*, 12*S*)-**4**

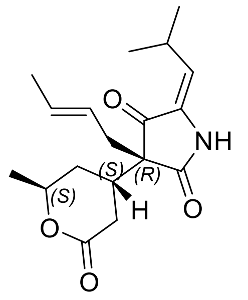

M-(3*R*, 10*S*, 12*S*)-**4**

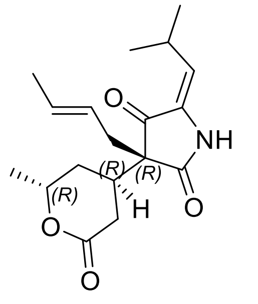

M-(3*R*, 10*R*, 12*R*)-**4**

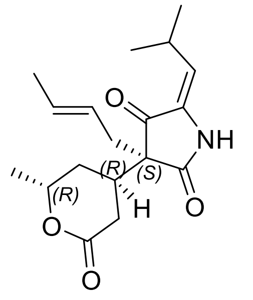

M-(3*S*, 10*R*, 12*R*)-**4**

Figure S38. Truncated structures of four possible isomers of **4** for ECD calculations.

Table S7. Energies of model-**4** at MMFF94 force field.

Configuration	Conformer	Energy (kcal/mol)	Population (%)
M-(3*S*, 10*S*, 12*S*)-**4**	1	86.94	48.1
M-(3*S*, 10*S*, 12*S*)-**4**	2	88.55	25.1

M-(3*S*, 10*S*, 12*S*)-**4**	3	91.12	8.9
M-(3*S*, 10*S*, 12*S*)-**4**	4	92.28	5.6
M-(3*S*, 10*S*, 12*S*)-**4**	5	92.44	5.2
M-(3*S*, 10*S*, 12*S*)-**4**	6	94.22	2.5
M-(3*S*, 10*S*, 12*S*)-**4**	7	94.32	2.5
M-(3*S*, 10*S*, 12*S*)-**4**	8	98.87	0.4
M-(3*R*, 10*S*, 12*S*)-**4**	1	87.07	42.4
M-(3*R*, 10*S*, 12*S*)-**4**	2	88.21	26.8
M-(3*R*, 10*S*, 12*S*)-**4**	3	91.31	7.7
M-(3*R*, 10*S*, 12*S*)-**4**	4	91.74	6.5
M-(3*R*, 10*S*, 12*S*)-**4**	5	91.78	6.3
M-(3*R*, 10*S*, 12*S*)-**4**	6	93.13	3.7
M-(3*R*, 10*S*, 12*S*)-**4**	7	93.95	2.6
M-(3*R*, 10*S*, 12*S*)-**4**	8	94.30	2.3
M-(3*R*, 10*S*, 12*S*)-**4**	9	99.38	0.3
M-(3*R*, 10*R*, 12*R*)-**4**	1	86.94	45.7
M-(3*R*, 10*R*, 12*R*)-**4**	2	88.55	23.8
M-(3*R*, 10*R*, 12*R*)-**4**	3	91.12	8.4
M-(3*R*, 10*R*, 12*R*)-**4**	4	92.28	5.3
M-(3*R*, 10*R*, 12*R*)-**4**	5	92.37	5.1
M-(3*R*, 10*R*, 12*R*)-**4**	6	92.44	5.0
M-(3*R*, 10*R*, 12*R*)-**4**	7	94.22	2.4
M-(3*R*, 10*R*, 12*R*)-**4**	8	94.32	2.3
M-(3*R*, 10*R*, 12*R*)-**4**	9	98.87	0.4
M-(3*S*, 10*R*, 12*R*)-**4**	1	87.07	42.3
M-(3*S*, 10*R*, 12*R*)-**4**	2	88.21	26.7
M-(3*S*, 10*R*, 12*R*)-**4**	3	91.31	7.6
M-(3*S*, 10*R*, 12*R*)-**4**	4	91.74	6.4
M-(3*S*, 10*R*, 12*R*)-**4**	5	91.78	6.3

M-(3*S*, 10*R*, 12*R*)-**4**	6	93.13	3.7
M-(3*S*, 10*R*, 12*R*)-**4**	7	93.95	2.6
M-(3*S*, 10*R*, 12*R*)-**4**	8	94.30	2.3
M-(3*S*, 10*R*, 12*R*)-**4**	9	99.38	0.3

Table S8. Energies of model-**4** at B3LYP/6–31+g(d) level in methanol.

Configuration	Conf.	E (Hartree)	E (kcal/mol)	Population (%)
M-(3*S*, 10*S*, 12*S*)-**4**	1	–1056.9990371	–663277.465770621	15.28
M-(3*S*, 10*S*, 12*S*)-**4**	2	–1056.998941	–663277.40546691	13.80
M-(3*S*, 10*S*, 12*S*)-**4**	3	–1056.9998929	–663278.002793679	37.86
M-(3*S*, 10*S*, 12*S*)-**4**	4	–1056.9990143	–663277.451463393	14.92
M-(3*S*, 10*S*, 12*S*)-**4**	5	–1056.9986821	–663277.243004571	10.49
M-(3*S*, 10*S*, 12*S*)-**4**	6	–1056.9976815	–663276.615118065	3.63
M-(3*S*, 10*S*, 12*S*)-**4**	7	–1056.9973831	–663276.427869081	2.65
M-(3*S*, 10*S*, 12*S*)-**4**	8	–1056.9967595	–663276.036553845	1.37
M-(3*R*, 10*S*, 12*S*)-**4**	1	–1056.998769	–663277.29753519	8.70
M-(3*R*, 10*S*, 12*S*)-**4**	2	–1056.9992563	–663277.603320813	14.58
M-(3*R*, 10*S*, 12*S*)-**4**	3	–1056.9999991	–663278.069435241	32.04
M-(3*R*, 10*S*, 12*S*)-**4**	4	–1056.9996399	–663277.844033649	21.89
M-(3*R*, 10*S*, 12*S*)-**4**	5	–1056.9989287	–663277.397748537	10.30
M-(3*R*, 10*S*, 12*S*)-**4**	6	–1056.9983179	–663277.014465429	5.39
M-(3*R*, 10*S*, 12*S*)-**4**	7	–1056.9983012	–663277.003986012	5.30
M-(3*R*, 10*S*, 12*S*)-**4**	8	–1056.9967716	–663276.044146716	1.05
M-(3*R*, 10*S*, 12*S*)-**4**	9	–1056.996466	–663275.85237966	0.76
M-(3*R*, 10*R*,12*R*)-**4**	1	–1056.999037	–663277.46570787	12.76
M-(3*R*, 10*R*,12*R*)-**4**	2	–1056.9989411	–663277.405529661	11.53
M-(3*R*, 10*R*,12*R*)-**4**	3	–1056.9998934	–663278.003107434	31.63
M-(3*R*, 10*R*,12*R*)-**4**	4	–1056.9990144	–663277.451526144	12.46
M-(3*R*, 10*R*,12*R*)-**4**	5	–1056.9992783	–663277.617126033	16.48

M-(3*R*, 10*R*,12*R*)-**4**	6	–1056.9986821	–663277.243004571	8.76
M-(3*R*, 10*R*,12*R*)-**4**	7	–1056.997682	–663276.61543182	3.03
M-(3*R*, 10*R*,12*R*)-**4**	8	–1056.9973829	–663276.427743579	2.21
M-(3*R*, 10*R*,12*R*)-**4**	9	–1056.9967594	–663276.036491094	1.14
M-(3*S*, 10*R*,12*R*)-**4**	1	–1056.998769	–663277.29753519	12.76
M-(3*S*, 10*R*,12*R*)-**4**	2	–1056.999257	–663277.60376007	11.53
M-(3*S*, 10*R*,12*R*)-**4**	3	–1056.999999	–663278.06937249	31.63
M-(3*S*, 10*R*,12*R*)-**4**	4	–1056.9996398	–663277.843970898	12.46
M-(3*S*, 10*R*,12*R*)-**4**	5	–1056.9989288	–663277.397811288	16.48
M-(3*S*, 10*R*,12*R*)-**4**	6	–1056.9983179	–663277.014465429	8.76
M-(3*S*, 10*R*,12*R*)-**4**	7	–1056.9983013	–663277.004048763	3.03
M-(3*S*, 10*R*,12*R*)-**4**	8	–1056.9967716	–663276.044146716	2.21
M-(3*S*, 10*R*,12*R*)-**4**	9	–1056.996466	–663275.85237966	1.14

M-(3*S*, 10*S*, 12*S*)-**4**

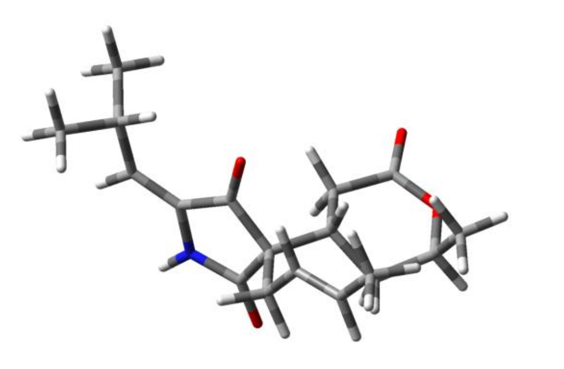

Conf.1 (15.28%)

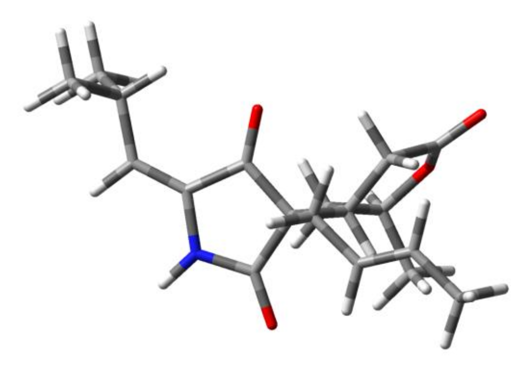

Conf.2 (13.80%)

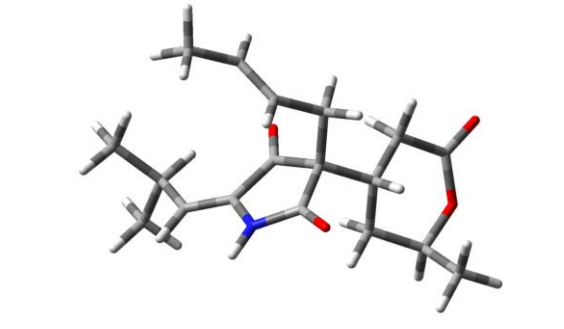

Conf.3 (37.86%)

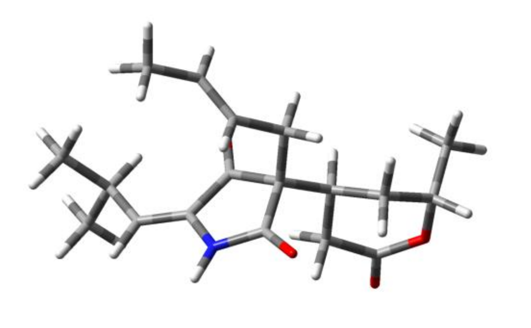

Conf.4 (14.92%)

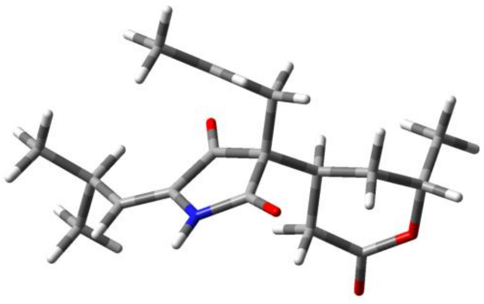	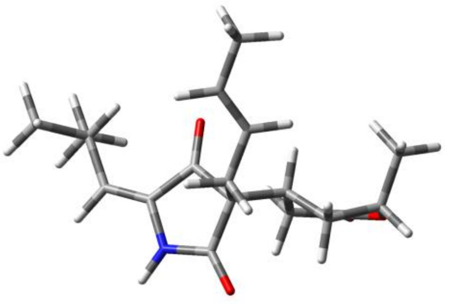
Conf.5 (10.49%)	Conf.6 (3.63%)
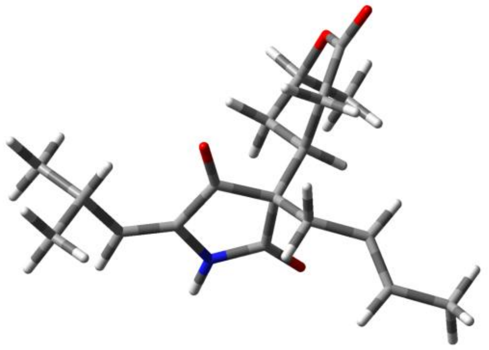	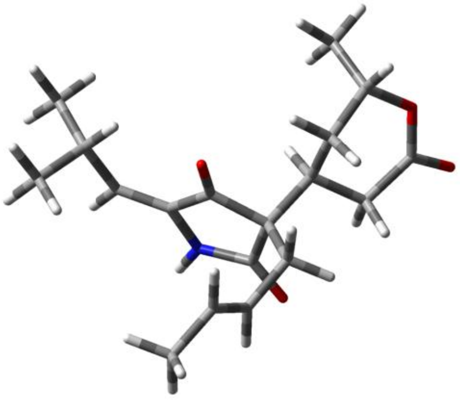
Conf.7 (2.65%)	Conf.8 (1.37%)

M-(3*R*, 10*S*, 12*S*)-**4**

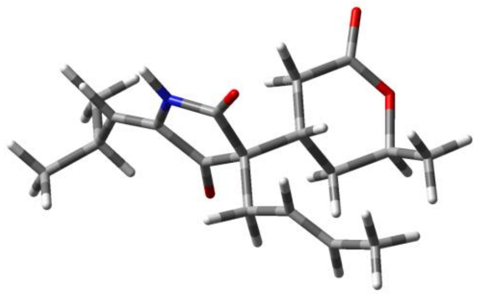	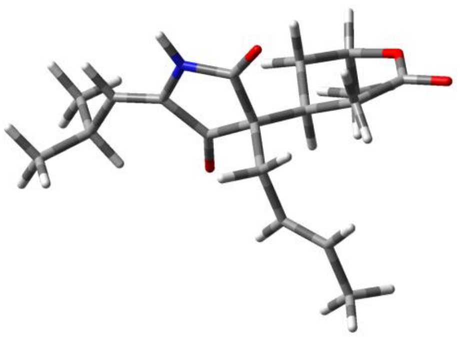
Conf.1 (8.70%)	Conf.2 (14.58%)
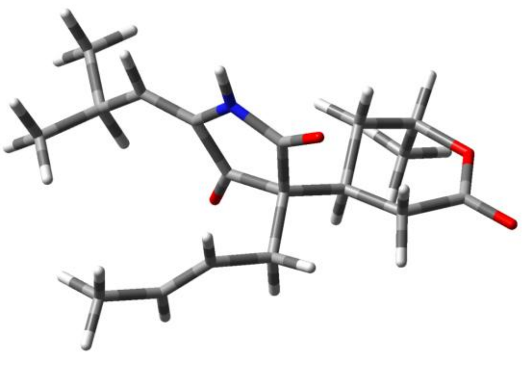	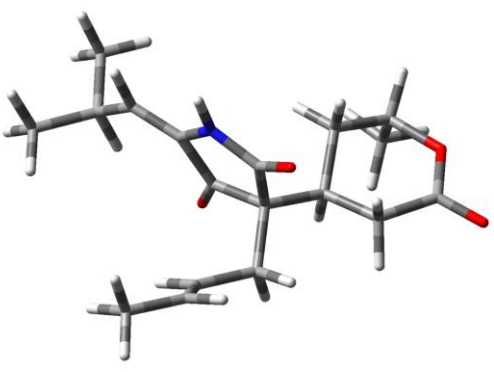

Conf.3 (32.04%)	Conf.4 (21.89%)
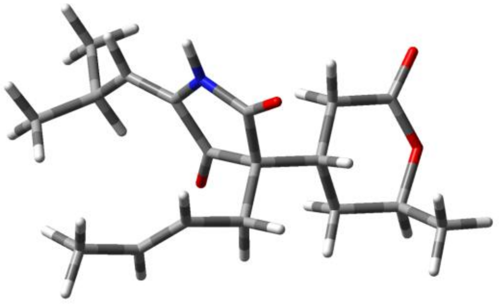	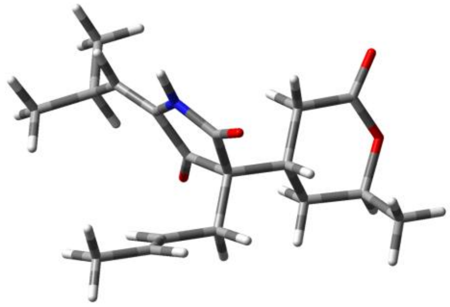
Conf.5 (10.30%)	Conf.6 (5.39%)
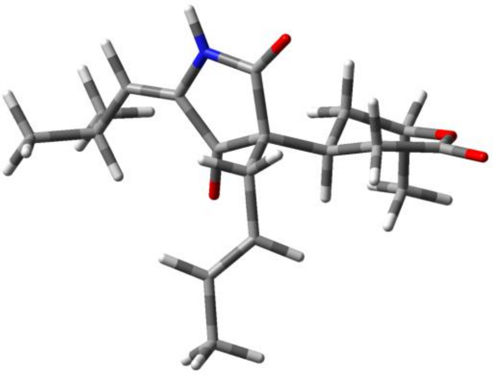	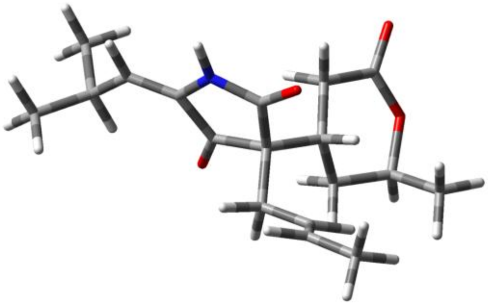
Conf.7 (5.30%)	Conf.8 (1.05%)
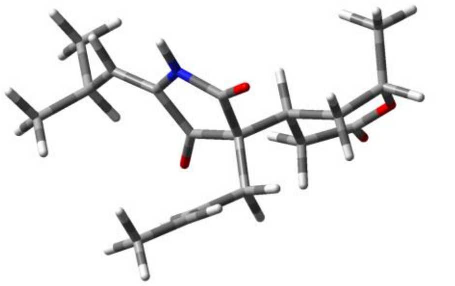	
Conf.9 (0.76%)	

M-(3*R*, 10*R*, 12*R*)-**4**

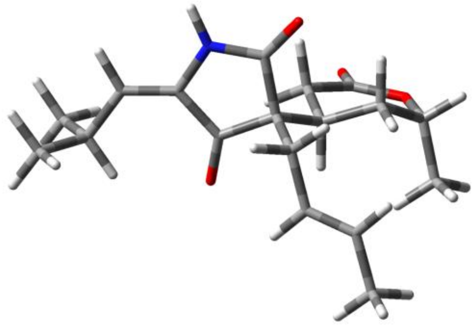	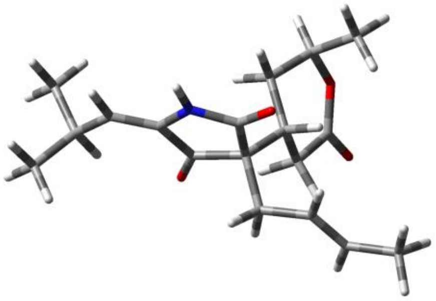

Conf.1 (12.76%)	Conf.2 (11.53%)
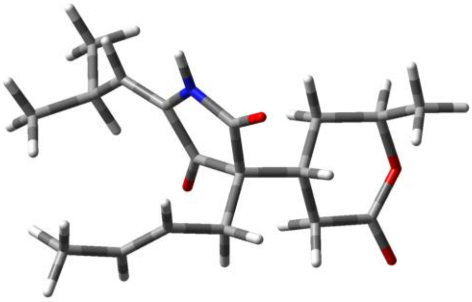	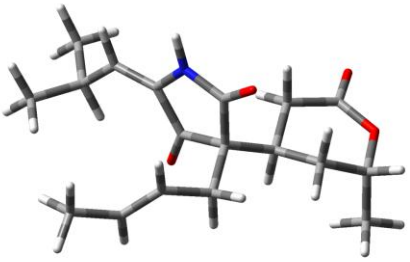
Conf.3 (31.63%)	Conf.4 (12.46%)
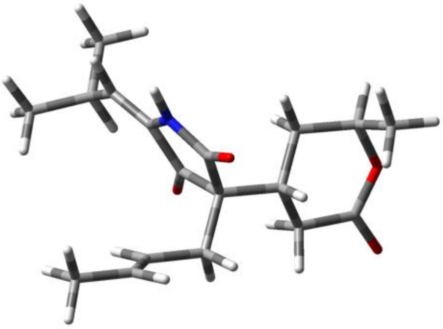	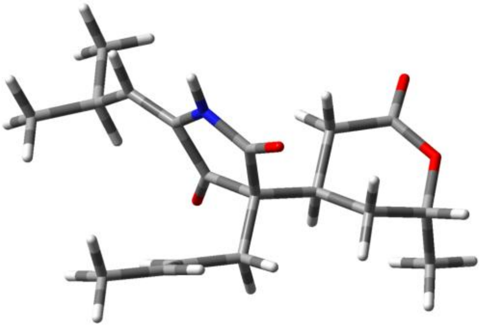
Conf.5 (16.48%)	Conf.6 (8.76%)
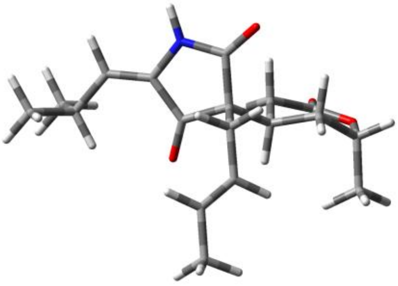	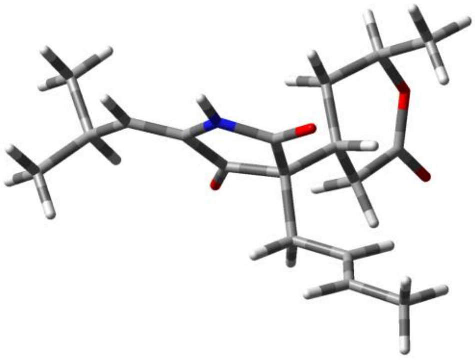
Conf.7 (3.03%)	Conf.8 (2.21%)
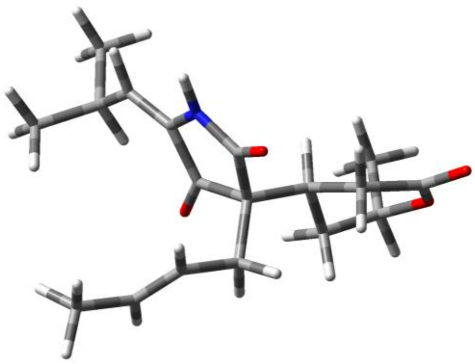	

Conf.9 (1.14%)	

M-(3*S*, 10*R*, 12*R*)-**4**

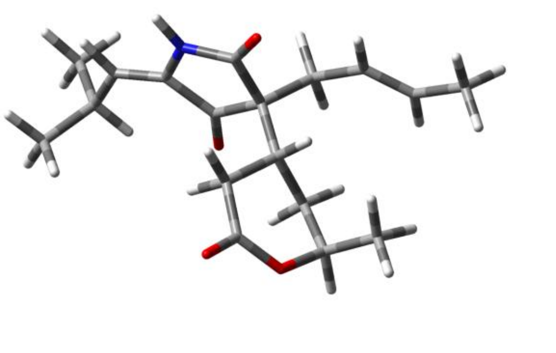	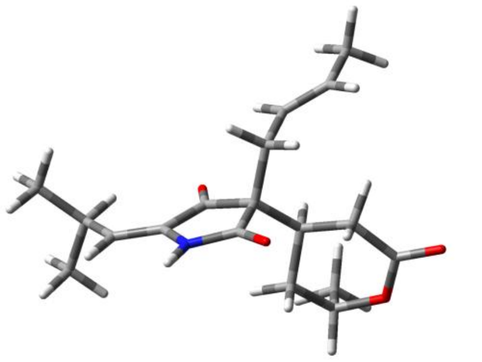
Conf.1 (12.76%)	Conf.2 (11.53%)
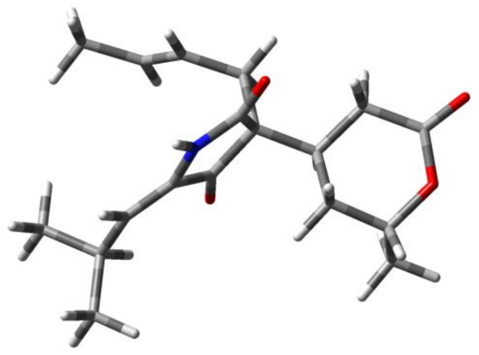	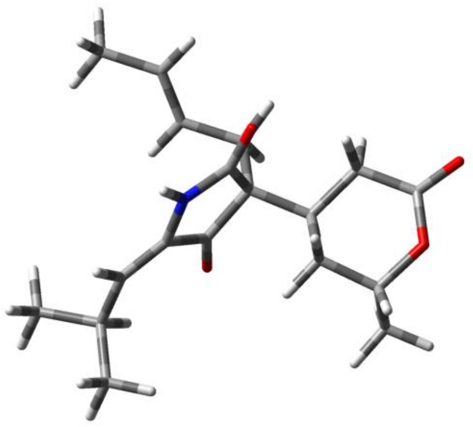
Conf.3 (31.63%)	Conf.4 (12.46%)
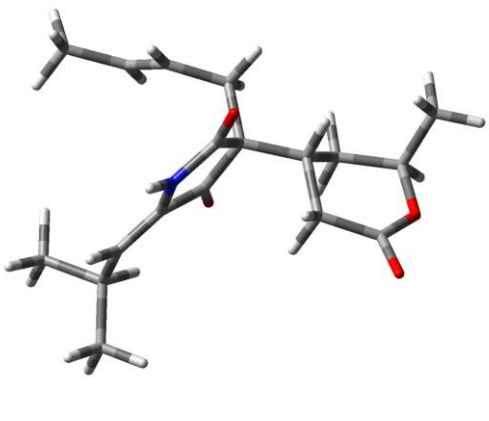	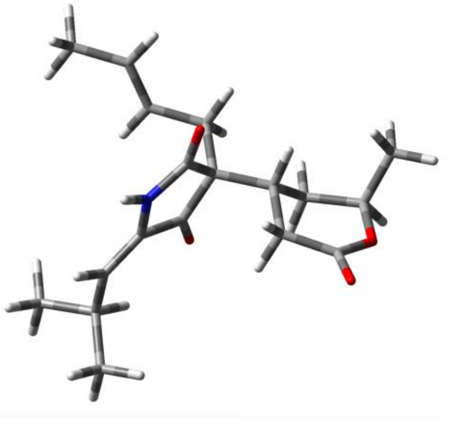
Conf.5 (16.48%)	Conf.6 (8.76%)

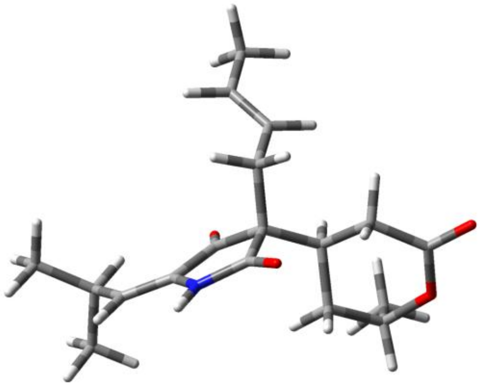	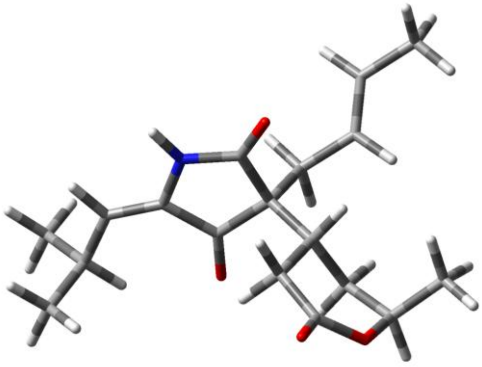
Conf.7 (3.03%)	Conf.8 (2.21%)
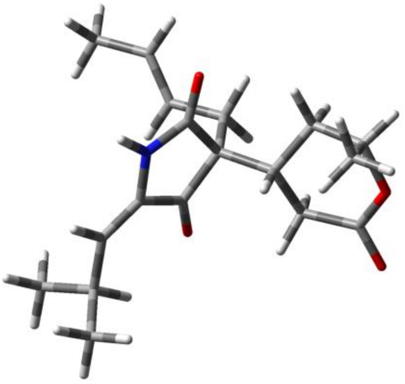	
Conf.9 (1.14%)	

Figure S39. The optimized conformers and equilibrium populations of model-**4**.

The ITS sequence of *Montagnula* sp. GXIMD 02514

CGTACAGCCTTAACCTTATCTATGCGTACCTTCACATTCTCCTTCGGCGGGGTAACGCCC

GCCGTCGGAACAACAAACCCATTTGCATCTAGTATTCTACCAGTTCTGATAAAAATTCA

ATCGTTACAACTTTCAACAATGGATCTCTTGGCTCTGGCATCGATGAAGAACGCAGCGA

AATGCGATAAGTAGTGTGAATTGCAGAATTCAGTGAATCATCGAATCTTTGAACGCACA

TTGCGCCCCTTGGTATTCCATGGGGCATGCCTGTTCGAGCGTCATCTACACCCTCAAGC

TCTGCTTGGTGTTGGGCGTCTGTCCCGCCTCCGCGCGGACTCGCCCCAAATCCATTG

GCAGCGGTCCTTGCCCCCTCTCGCGCAGCACATTGCGCTCCTCGAGGGCGGCTCCGGG

CCTGCGACCCACGAAGATGACCCGTCTTTTGACCTCGGATCAGGTAGGGATACCCGCT

GAACTTAAGCATATCA
